# Effect of a Daily Text Messaging and Directly Supervised Therapy Intervention on Oral Mercaptopurine Adherence in Children With Acute Lymphoblastic Leukemia: A Randomized Clinical Trial

**DOI:** 10.1001/jamanetworkopen.2020.14205

**Published:** 2020-08-27

**Authors:** Smita Bhatia, Lindsey Hageman, Yanjun Chen, F. Lennie Wong, Elizabeth L. McQuaid, Christina Duncan, Leo Mascarenhas, David Freyer, Nkechi Mba, Paula Aristizabal, David Walterhouse, Glen Lew, Pamela Helen-Heilge Kempert, Thomas Bennett Russell, Rene Y. McNall-Knapp, Shana Jacobs, Ha Dang, Elizabeth Raetz, Mary V. Relling, Wendy Landier

**Affiliations:** 1 Institute for Cancer Outcomes and Survivorship, University of Alabama at Birmingham, Birmingham; 2 Department of Population Sciences, City of Hope, Duarte, California; 3 Department of Psychiatryand Human Behavior, Brown University, Providence, Rhode Island; 4 Department of Psychology,West Virginia University, Morgantown; 5 Cancer and Blood Disease Institute, Division of Hematology/Oncology, Children’s Hospital Los Angeles, Los Angeles, California; 6 Department of Pediatrics, Keck School of Medicine, University of Southern California, Los Angeles; 7 Department of Pediatric Hematology/Oncology, Driscoll Children’s Hospital, Corpus Christi, Texas; 8 Department of Pediatrics, University of California, San Diego, San Diego; 9 Department of Pediatrics, Division of Hematology, Oncology, and Stem Cell Transplantation, Ann & Robert H Lurie Children’s Hospital of Chicago, Chicago, Illinois; 10 Aflac Cancer and Blood Disorders Center, Children’s Healthcare of Atlanta, Emory University, Atlanta, Georgia; 11 Department of Hematology/Oncology, Miller Children’s and Women’s Hospital, Long Beach, California; 12 Wake Forest Baptist Comprehensive Cancer Center, Wake Forest University Health Sciences, Winston-Salem, North Carolina; 13 Department of Pediatrics, University of Oklahoma Health Sciences Center, Oklahoma City; 14 Department of Oncology, Children’s National Medical Center, Washington, DC; 15 Department of Preventive Medicine, Keck School of Medicine, University of Southern California, Los Angeles; 16 Department of Pediatrics, NYU Langone Medical Center, New York, New York; 17 Department of Pharmaceutical Sciences, St Jude Children’s Research Hospital, Memphis, Tennessee

## Abstract

**Importance:**

Suboptimal adherence to oral mercaptopurine treatment in children with acute lymphoblastic leukemia (ALL) increases the risk of relapse. A frequently expressed barrier to adherence is forgetfulness, which is often overcome by parental vigilance.

**Objective:**

To determine whether a multicomponent intervention, compared with education alone, will result in a higher proportion of patients with ALL who have mercaptopurine adherence rates 95% or higher, for all study participants and among patients younger than 12 years and vs those aged 12 years and older.

**Design, Setting, and Participants:**

The adherence intervention trial was an investigator-initiated, multi-institutional, parallel-group, unblinded, randomized clinical trial conducted between July 16, 2012, and August 8, 2018, at 59 Children’s Oncology Group institutions in the US, enrolling patients with ALL diagnosed through age 21 years and receiving mercaptopurine for maintenance. The date of final follow-up was January 2, 2019. Data analysis was performed from February to October 2019.

**Interventions:**

Patients were randomized 1:1 to education alone or the intervention package, which consisted of education and personalized text message reminders daily to prompt directly supervised therapy. Four weeks of baseline adherence monitoring were followed with a 16-week intervention.

**Main Outcomes and Measures:**

The primary end point was the proportion of patients with adherence rates 95% or higher over the duration of the intervention for all study participants, and for those younger than 12 years vs those aged 12 years and older.

**Results:**

There were 444 evaluable patients (median age, 8.1 years; interquartile range, 5.3-14.3 years), including 230 in the intervention group and 214 in the education group. Three hundred two patients (68.0%) were boys, 180 (40.5%) were non-Hispanic White, 170 (38.3%) were Hispanic, 43 (9.7%) were African American, and 51 (11.5%) were Asian or of mixed race/ethnicity. The proportion of patients with adherence rates 95% or higher did not differ between the intervention vs education groups (65% vs 59%; odds ratio, 1.33; 95% CI, 1.0-2.0; *P* = .08). Exploratory analyses showed that among patients aged 12 years and older, those in the intervention group had higher mean (SE) adherence rates than those in the education group (93.1% [1.1%] vs 90.0% [1.3%]; difference, 3.1%; 95% CI, 0.1%-6.0%; *P* = .04). In particular, among patients aged 12 years and older with baseline adherence less than 90%, those in the intervention group had higher mean (SE) adherence rates than those in the education group (83.4% [2.5%] vs 74.6% [3.4%]; difference, 8.8%; 95% CI, 2.2%-15.4%; *P* = .008). No safety concerns were identified.

**Conclusions and Relevance:**

Although this multicomponent intervention did not result in an increase in the proportion of patients with ALL who had mercaptopurine adherence rates 95% or higher, it did identify a high-risk subpopulation to target for future adherence intervention strategies: adolescents with low baseline adherence.

**Trial Registration:**

ClinicalTrials.gov Identifier: NCT01503632

## Key Points

**Question:**

In children with acute lymphoblastic leukemia receiving oral mercaptopurine, can an intervention consisting of education and daily text message reminders to prompt directly supervised therapy result in a higher proportion of patients with mercaptopurine adherence 95% or higher, compared with education alone?

**Findings:**

In this parallel-group, unblinded, randomized clinical trial including 444 children with acute lymphoblastic leukemia, the proportion of patients with mercaptopurine adherence rates 95% or higher did not differ between the intervention and education groups. In exploratory analyses, children aged 12 years and older with baseline adherence less than 90% had higher mean adherence in the intervention group.

**Meaning:**

This study provides evidence for limiting future trials to older children with acute lymphoblastic leukemia who have low baseline adherence to oral mercaptopurine.

## Teaser

This randomized clinical trial examines whether a multicomponent intervention results in a higher proportion of pediatric patients with acute lymphoblastic leukemia maintaining mercaptopurine adherence rates of 95% or higher compared with education alone.

## Introduction

Acute lymphoblastic leukemia (ALL) is the most common childhood cancer.^1^ Although more than 95% of children with ALL enter remission after a 4-week induction, approximately 20% relapse within 5 years.^1^ Second-line therapies are toxic, and salvage is poor.^2^ Durable first clinical remission is desirable and requires a prolonged maintenance phase with daily self-administered oral mercaptopurine. Low mercaptopurine systemic exposure increases relapse risk.^3,4,5^ Patient adherence to mercaptopurine is a primary determinant of mercaptopurine systemic exposure.^6^ Adherence to medication is a process by which patients take their medication as prescribed and typically consists of 3 phases: initiation, implementation, and discontinuation.^7,8^ In the present study and our prior studies,^8^ we focus on the implementation phase of mercaptopurine adherence, in which patients have initiated but have not yet discontinued mercaptopurine. In a previous Children’s Oncology Group (COG) study (AALL03N1), we measured mercaptopurine adherence electronically in children with ALL entering maintenance during the first clinical remission, using the Medication Event Monitoring System (MEMS) TrackCap device (Aprex Corp).^6,9,10^ Poor mercaptopurine adherence (mean adherence rates, <90% to 95%) was associated with a 2.5- to 3.9-fold higher relapse risk; up to 59% of ALL relapses were attributable to poor mercaptopurine adherence.^6,9,10^ The most common reason for missing mercaptopurine was forgetfulness^9^; adherent patients and parents endorsed parental vigilance as a strategy to overcome forgetfulness.^11^ Given the critical need to improve mercaptopurine adherence in children with ALL, we conducted a randomized clinical trial (COG ACCL1033) in which children with ALL receiving mercaptopurine maintenance were randomly assigned to receive either education alone or a comprehensive adherence-enhancing intervention in addition to education. We wished to test the hypothesis that the adherence-enhancing intervention consisting of education and daily text message reminders to prompt directly supervised therapy (DST) would result in a higher proportion of patients with mercaptopurine adherence rates 95% or higher compared with education alone.

## Methods

### Trial Oversight

**Figure 1.**
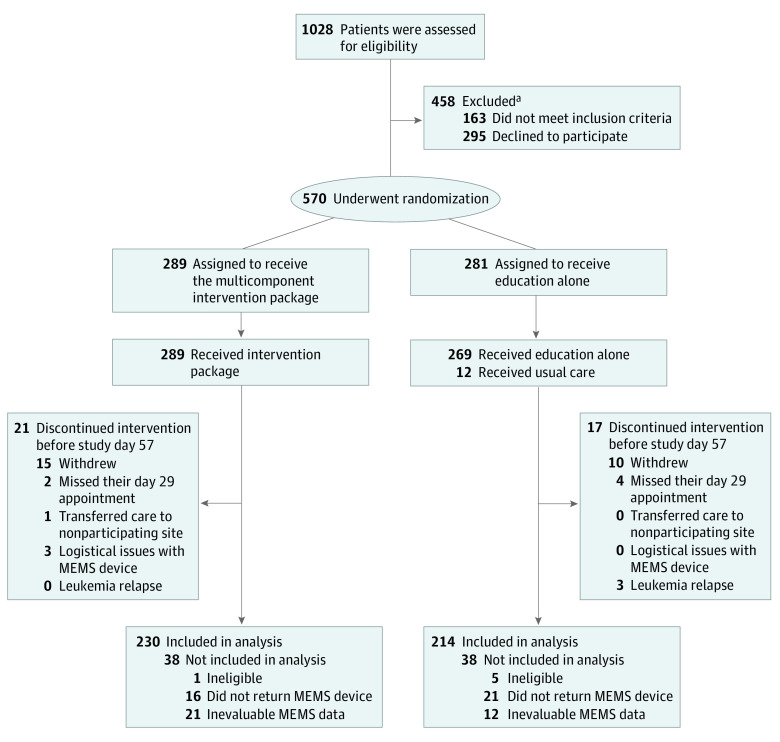
Enrollment and Retention of Patients by Study Group Day 29 and day 57 represent days during the trial when the patient returned for a scheduled clinic visit. ^a^Among the 163 patients who did not meet the inclusion criteria, 111 were using a pillbox, 20 did not want to receive text message reminders, 12 did not want to use the Medication Event Monitoring System (MEMS) device, 5 did not have a designated caregiver, and 15 had other reasons. Among the 295 patients who declined participation, 114 were not interested, 62 had another reminder system, 24 said they already remembered, 23 said they were too busy or the timing was bad, 3 had physician refusal, and the other 69 had other or no reasons. Lack of access to a text-capable device was not a confounding feature, because for patients who did not have access to a cellular telephone with texting capabilities, we issued a study telephone with unlimited data for the entire study period.

The National Cancer Institute’s pediatric central institutional review board and institutional review boards at participating sites approved the trial protocol (Supplement 1). ACCL1033 was an investigator-initiated, unblinded, parallel-group, randomized clinical trial with 1:1 randomization, conducted at 59 COG sites in the US (eAppendix 1 in Supplement 2), in accordance with the principles of Guidelines for Good Clinical Practice, the Declaration of Helsinki, and all applicable local regulations. A data monitoring committee at the University of Alabama at Birmingham oversaw the safety of this trial. Written informed consent or assent was obtained from participating patients and/or parents. This study follows the Consolidated Standards of Reporting Trials (CONSORT) reporting guideline for parallel group randomized trials^12^ (Figure 1) and the European Society for Patient Adherence, Compliance, and Persistence Medication Adherence reporting guidelines.^7^

### Study Participants

Eligibility criteria included diagnosis of ALL at age 1 year or later and up through age 21 years; in first clinical remission; receiving mercaptopurine during the maintenance phase of therapy for at least 24 weeks and scheduled to receive at least 24 more weeks; having a designated parent or caregiver willing to participate in a daily supervised medication administration; able and willing to use the MEMS TrackCap device; parent or caregiver and patient (if aged ≥12 years) willing to receive medication reminders via text messaging; and English- or Spanish-speaking. We excluded patients with Down syndrome and those who previously participated or were currently participating in another adherence-enhancing intervention. Adherence rate at study entry was not an eligibility criterion.

### Random Assignment and Study Interventions

#### Randomization

Eligible participants were randomly assigned 1:1 to the intervention group or education alone using blocked stratified randomization with age at study (<12 vs ≥12 years) and race/ethnicity (non-Hispanic White, Hispanic, African American, and Asian or mixed race/ethnicity) as stratification factors. Randomization occurred centrally at the COG Statistics and Data Center. Participants, investigators, and study personnel were informed of the randomization assignment following collection of baseline data.

#### Intervention

The intervention was based on the Extended Health Belief Model^13^ (eAppendix 2 in Supplement 2), was directed at the patient and parent or caregiver, and addressed the implementation phase of adherence (ie, patients taking mercaptopurine during the period after initiation and before discontinuation). The intervention consisted of education and daily personalized text message reminders from the treating oncologist to the patient and parent to prompt DST. We summarize the individual intervention components here and provide additional details in eAppendix 2 in Supplement 2.

#### Education Program

The interactive multimedia educational program used video vignettes (created by W.L.) of patients and parents drawn from diverse sociodemographic backgrounds to address health beliefs, including perceived susceptibility to or severity of ALL, purpose of mercaptopurine, perceived benefits or barriers to mercaptopurine ingestion, and examples of how patients or parents overcame such barriers. Study participants self-tailored their viewing to fit their learning needs and linguistic preferences (English or Spanish).

#### Text Message Reminders

A secure, HIPAA (Health Insurance Portability and Accountability Act)–compliant web-based application developed by MedActionPlan/CT was created for patients participating in the ACCL1033 study. It allows the treating oncologist to activate automated personalized text messaging reminders delivered daily via cellular telephone to patients and parents (if the patient is aged ≥12 years) or to parents alone (if the patient is aged <12 years).

#### Directly Supervised Therapy

The patients aged 12 years and older and their parents, and only the parents of the patients younger than 12 years, used the text message as a cue that mercaptopurine was due, prompting mercaptopurine ingestion by the patient and supervision or administration by the parent. Once the patient had taken the mercaptopurine, the patient aged 12 years and older and their parent, or only the parent of the patient younger than 12 years, responded to the text message with a reply function on the cellular telephone, indicating DST execution.

### Timeline

For the first 28 days, all participants in both the intervention and education groups received mercaptopurine from a MEMS device (eAppendix 2 in Supplement 2) without intervention to calculate baseline adherence rates. The intervention began on day 29 and lasted for 16 weeks. Patients or parents in both groups viewed the educational video during scheduled clinic visits on day 29. For patients in the intervention group, the treating oncologist activated automated daily text message reminders on day 29 and renewed these reminders every 28 days until the study’s end. Research staff at participating sites provided details regarding prescribed mercaptopurine dose for each day of the trial and dates when mercaptopurine was withheld for toxicity or illness (eAppendix 2 in Supplement 2).

### Study End Points

The primary end points were defined as the proportion of patients with mercaptopurine adherence rate 95% or higher in both groups for the entire cohort, for patients aged 12 years and older, and for patients younger than 12 years. We assessed adherence using the MEMS adherence-monitoring device, which uses microelectronic technology to record the date and time of mercaptopurine bottle openings. We informed the patients and parents about the purpose of the MEMS device. The participating sites returned the MEMS device to the coordinating center (University of Alabama at Birmingham) upon study completion, where we downloaded the data. The MEMS-based adherence rate was defined as the ratio of days with MEMS device openings (*N*) to the number of days mercaptopurine was prescribed (*D*) for each patient, reported as a percentage (*N */* D *×* *100). We removed the days when the prescriber withheld the mercaptopurine dose from the denominator. We calculated monthly mean adherence rates for each patient for the entire cohort and by age at study entry (<12 years vs ≥12 years) and used these to derive the proportion of patients with adherence rates 95% or higher. We chose to examine the effect of interventions in patients aged 12 years and older and those younger than 12 years, on the basis of our prior work,^10^ which showed that mean mercaptopurine adherence was substantially lower in patients aged 12 years and older compared with those younger than 12 years. End points examined in the exploratory analyses included longitudinal mean monthly adherence rates in the intervention vs education groups for the entire cohort, patients younger than 12 years, and patients aged 12 years and older, and by baseline mercaptopurine adherence rates (<90% and ≥90%).

### Statistical Analysis

Using an intention-to-treat analysis, we tested the efficacy of the intervention by comparing group differences in the proportion of adherent patients at each month after the intervention using logistic regression with generalized estimating equations. The proportions of patients with adherence rates 95% or higher in both groups were determined for the entire cohort, for patients aged 12 years and older, and for those younger than 12 years. To account for the 3 independent tests, a 2-sided Bonferroni-adjusted type I error of .017 was used for each test. To calculate sample sizes projected to have 80% power to detect meaningful differences, we assumed values for the proportion of patients with adherence 95% or higher in the education group according to our previous study,^6^ as well as 2 additional values in increments of 0.5 (entire cohort, 0.56, 0.61, and 0.66; patients <12 years old, 0.60, 0.65, and 0.70; patients ≥12 years old, 0.42, 0.47, and 0.52). We projected the following sample sizes: for the entire cohort, we would need 228 patients per group to detect a difference between the proportion of patients with adherence 95% or higher in the education group of 0.56 to 0.66 vs 0.70 to 0.79 in the intervention group, or an odds ratio (OR) of 1.9 to 2.0; for patients younger than 12 years, we would need 133 patients per group to detect a difference between the proportion of patients with adherence 95% or higher in the education group of 0.60 to 0.70 vs 0.78 to 0.86 in the intervention group, or an OR of 2.4 to 2.7; and for patients aged 12 years and older, we would need 95 patients per group to detect a difference between the proportion of patients with adherence 95% or higher in the education group of 0.42 to 0.52 vs 0.65 to 0.74 in the intervention group, or an OR of 1.9 to 2.0. Using generalized estimating equations, we also conducted exploratory analyses where we compared the longitudinal mean monthly adherence rates between the intervention and education groups among all patients, those younger than 12 years, those aged 12 years and older, and within subgroups of patients according to baseline mercaptopurine adherence rates (<90% and ≥90%).

Baseline patient characteristics that varied between treatment groups were included as covariates to obtain unbiased estimates of the intervention effect. We handled missing data by multiple imputation, assuming data missing at random, by generating 20 sets of complete data using the predictive mean matching method for continuous variables, and logistic and polytomous regression imputation for binary and categorical variables. We performed sensitivity analyses to assess the missing at random assumption.^14^ We performed the analyses with SAS statistical software version 9.4 (SAS Institute) and R statistical software version 3.6.1 (R Project for Statistical Computing). The complete statistical analysis plan is included in the trial protocol (Supplement 1). Data analysis was performed from February to October 2019.

## Results

Of the 570 patients enrolled at 59 participating sites between July 16, 2012, and August 8, 2018, 444 (78.0%) were evaluable, including 230 in the intervention group and 214 in the education group (Figure 1). The median age at study enrollment was 8.1 years (interquartile range [IQR], 5.3-14.3 years) overall, 8.6 years (IQR, 5.6-14.3 years) for the intervention group, and 7.5 years (IQR, 5.3-14.0 years) for the education group. Three hundred two patients (68.0%) were boys, 180 (40.5%) were non-Hispanic White, 170 (38.3%) were Hispanic, 43 (9.7%) were African American, and 51 (11.5%) were Asian or of mixed race/ethnicity. Of these, 378 (85%) had precursor B-cell disease and 66 (15%) had T-cell disease. All patients had received treatment per COG therapeutic protocols (AALL0932, 133 patients; AALL1131, 129 patients; AALL0232, 58 patients; AALL0434, 46 patients; AALL0331, 14 patients; AALL1231, 6 patients; and other, 58 patients). Overall, baseline characteristics were comparable between the 2 groups (Table), with the exception of lower paternal education in the intervention group. Adherence monitoring occurred for 12 197 person-days before the start of the intervention and for 45 949 person-days over the course of the intervention. The date of final follow-up was January 2, 2019. At baseline, the mean (SE) adherence rates were comparable between the 2 groups for the entire cohort (intervention, 92.2% [0.9%] vs education, 93.5% [0.8%]), for the patients younger than 12 years (intervention, 93.3% [1.1%] vs education, 94.8% [0.9%]), and for the patients aged 12 years and older (intervention, 90.3% [1.5%] vs education, 91.0% [1.6%]). The trial ended when we completed our contract with MedActionPlan/CT and we had almost reached the prespecified target enrollment (444 / 456 = 97%).

**Table.** Demographic and Clinical Characteristics of Patients With Acute Lymphoblastic Leukemia at Baseline by Treatment Group^a^

Characteristic	Patients, No. (%)					
	Entire cohort		Age <12 y at study		Age ≥12 y at study	
	Intervention (n = 230)	Education (n = 214)	Intervention (n = 147)	Education (n = 143)	Intervention (n = 83)	Education (n = 71)
Age at diagnosis, median (IQR), y	6.8 (4.0-12.6)	5.7 (3.6-12.4)	4.5 (3.1-6.3)	3.96 (3.2-5.8)	14.1 (12.1-15.3)	14.5 (12.4-16.5)
Age at study start, median (IQR), y	8.6 (5.6-14.3)	7.5 (5.3-14)	6.12 (4.8-8.1)	5.63 (4.9-7.5)	15.7 (13.9-17.5)	16.4 (14-18.1)
Patients aged ≥12 y at study start	83 (36.1)	71 (33.2)	0	0	83 (100)	71 (100)
Male	154 (67.0)	148 (69.2)	98 (66.7)	93 (65.0)	56 (67.5)	55 (77.5)
Race/ethnicity^b^						
Non-Hispanic white	91 (39.6)	89 (41.6)	61 (41.5)	60 (41.9)	30 (36.1)	29 (40.9)
Hispanic	91 (39.6)	79 (36.9)	51 (34.7)	49 (34.3)	40 (48.2)	30 (42.3)
African American	20 (8.7)	23 (10.8)	13 (8.8)	16 (11.2)	7 (8.4)	7 (9.9)
Asian or mixed race/ethnicity	28 (12.2)	23 (10.8)	22 (15.0)	18 (12.6)	6 (7.2)	5 (7.0)
Parental education						
Paternal education less than or equal to high school	113 (49.1)	82 (38.3)	67 (45.6)	50 (35.0)	46 (55.4)	32 (45.1)
Maternal education less than or equal to high school	85 (37.0)	66 (30.8)	51 (34.7)	40 (28.0)	34 (40.9)	26 (36.6)
Annual household income, $						
<20 000	59 (25.6)	53 (24.8)	36 (24.5)	33 (23.1)	23 (27.7)	20 (28.2)
20 000-50 000	59 (25.7)	54 (25.2)	41 (27.9)	31 (21.7)	18 (21.7)	23 (32.4)
>50 000	98 (42.6)	88 (41.1)	61 (41.5)	70 (48.9)	37 (44.6)	18 (25.4)
Missing	14 (6.1)	19 (8.9)	9 (6.1)	9 (6.3)	5 (6.0)	10 (14.1)
Household structure						
Nuclear family	167 (72.6)	152 (71.0)	111 (75.5)	108 (75.5)	56 (67.5)	44 (61.9)
Single parent with multiple children	26 (11.3)	21 (9.8)	13 (8.8)	10 (7.0)	13 (15.7)	11 (15.5)
Single parent with single child	8 (3.5)	8 (3.7)	4 (2.7)	4 (2.8)	4 (4.8)	4 (5.6)
Other	29 (12.6)	33 (15.4)	19 (12.9)	21 (14.7)	10 (12.1)	11 (16.9)
Time between start of maintenance to study entry, median (IQR), y	0.9 (0.7-1.3)	0.9 (0.7-1.2)	0.91 (0.7-1.2)	0.90 (0.7-1.2)	0.90 (0.7-1.4)	0.88 (0.7-1.4)
Mercaptopurine dose intensity ratio at baseline, median (IQR)	0.89 (0.7-1)	0.90 (0.7-1)	0.89 (0.8-1)	0.89 (0.7-1)	0.85 (0.7-1)	0.93 (0.7-1)
Absolute neutrophil count, median (IQR), cells/μL	1980 (1500-2600)	1860 (1500-2500)	1940 (1400-2500)	1830 (1400-2500)	1990 (1600-2800)	1930 (1500-2400)
Baseline adherence rate, %						
Mean (SE)	92.2 (0.9)	93.5 (0.8)	93.3 (1.1)	94.8 (0.9)	90.3 (1.5)	91.0 (1.6)
Proportion with adherence <95%	32.2	29.5	26.2	26.5	42.7	35.2
Proportion with adherence <90%	24.2	19.8	17.9	14.7	35.4	29.6

Abbreviation: IQR, interquartile range.

SI conversion factor: To convert absolute neutrophil count to ×10^9^/L, multiply by 0.001.

^a^ The intervention package included education and daily personalized text message reminders prompting directly supervised therapy.

^b^ Race/ethnicity was self- or parent-reported, using options defined by the investigator, to ensure an adequate representation of African American and Hispanic patients, given our previous findings that indicated racial/ethnic differences in adherence.

### Primary End Point

Overall, 65% of the patients in the intervention group vs 59% of the patients in the education group achieved mercaptopurine adherence rates 95% or higher. After adjusting for baseline adherence, time in the study, and paternal education, the difference in proportion of patients with mercaptopurine adherence rate 95% or higher in the intervention and education groups did not reach statistical significance at the prespecified *P* value of 0.017 (entire cohort, OR, 1.33 [95% CI, 1.0-2.0]; *P* = .08; patients <12 years old, OR, 1.53 [95% CI, 1.0-2.2]; *P* = .04; patients ≥12 years old, OR, 0.8 [95% CI, 0.6-1.8]; *P* = .80).

### Exploratory Analyses

#### Adherence Rates in the Intervention vs Education Groups

**Figure 2.**
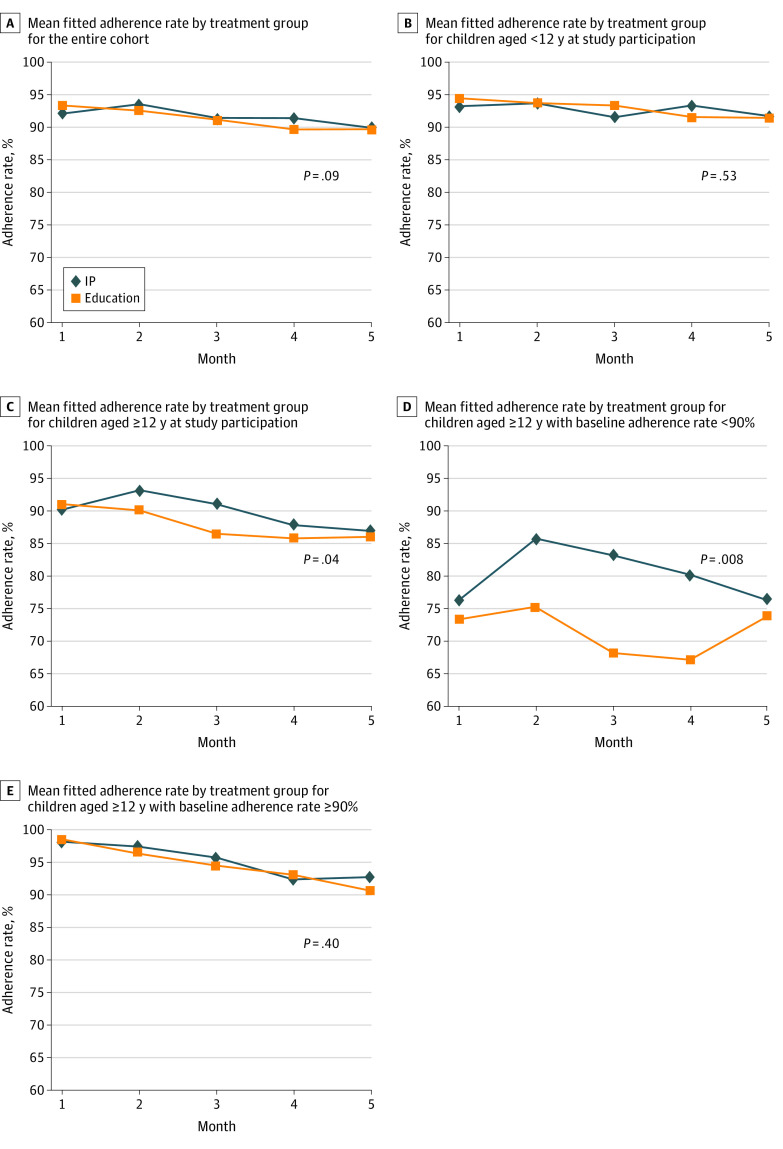
Mean Fitted Adherence Rate by Treatment Group, Intervention Package (IP) and Education Alone Graphs show adherence rates for entire cohort (A), for children younger than 12 years at study participation (B), for children aged 12 years and older at study participation (C), for children aged 12 years and older with baseline adherence less than 90% (D), and for children aged 12 years and older with baseline adherence greater than or equal to 90% (E). The IP included education and daily personalized text message reminders prompting directly supervised therapy.

The mean (SE) adherence rates for the intervention vs education groups for the entire cohort were 94.0% (0.6%) vs 92.5% (0.7%) (difference, 1.5%; 95% CI, −0.2% to 3.2%; *P* = .09) (Figure 2A). The corresponding mean (SE) adherence rates for the patients younger than 12 years were 94.4% (0.8%) vs 93.7% (0.8%) (difference, 0.7%; 95% CI, −1.5% to 2.8%; *P* = .53) (Figure 2B), and those for the patients aged 12 years and older were 93.1% (1.1%) vs 90.0% (1.3%) (difference, 3.1%; 95% CI, 0.1% to 6.0%, *P* = .04) (Figure 2C). For patients with baseline adherence rates less than 90%, there was no difference in mean (SE) adherence rates between the intervention and education groups for the entire cohort (79.8% [2.3%] vs 77.2% [2.7%]; difference, 2.6%; 95% CI, −3.6% to 8.8%; *P* = .40) or for the patients younger than 12 years (75.2% [3.9%] vs 81.3% [3.7%]; difference, −6.1%; 95% CI, −15.2% to 3.0%; *P* = .20). However, among patients aged 12 years and older with baseline adherence rates less than 90%, the mean (SE) adherence rates were significantly higher in the intervention group than in the education group (83.4% [2.5%] vs 74.6% [3.4%]; difference, 8.8%; 95% CI, 2.2% to 15.4%; *P* = .008) (Figure 2D); this was not true among patients in the same age group with baseline adherence rates 90% or higher (96.2% [0.8%] vs 95.0% [1.0%]; difference, 1.2%; 95% CI, −1.4% to 3.8%; *P* = .40) (Figure 2E).

#### Change From Preintervention to Postintervention Adherence Rates

**Figure 3.**
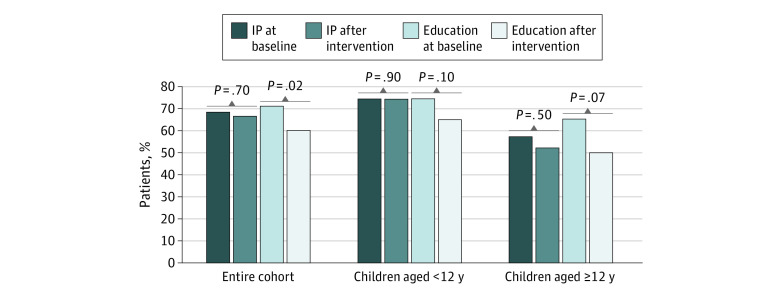
Proportion of Patients With Adherence Rates 95% or Higher Before and After the Intervention Period The intervention package (IP) included education and daily personalized text message reminders prompting directly supervised therapy. Proportions of patients were adjusted for baseline adherence, paternal education, and time in the adherence study.

Using multivariable binomial logistical regression analyses (adjusted for baseline adherence, paternal education, and time in the study), we found that in the entire cohort, the proportion of participants with adherence rates 95% or higher from baseline to the postintervention period remained stable for the intervention group, with rates of 68% and 66%, but decreased from 71% to 60% for the education group (Figure 3). Similar trends were observed for the 2 age categories: for patients younger than 12 years, the proportion with adherence rates 95% or higher remained at 74% from baseline to the postintervention period in the intervention group and decreased from 74% to 65% in the education group. For patients aged 12 years and older, the proportion with adherence rates 95% or higher decreased from 57% to 52% in the intervention group and from 65% to 50% in the education group.

We also examined the adherence rates from baseline to postintervention. The differences in the median (IQR) preintervention to postintervention adherence rates between the intervention and education groups were statistically significant (using nonparametric tests) for all patients (0.0% [IQR, −2.6% to 2.6%] vs −1.0% [IQR, −5.4% to 0.0%]; *P* < .001) and for those aged 12 years and older (0.0% [IQR, −1.9% to 3.6%] vs −1.8% [IQR, −7.1% to 0.0%]; *P* = .004), but not for those younger than 12 years (0.0% [IQR, −2.7% to 2.5%] vs −0.9% [IQR, −4.5% to 0.0%]; *P* = .06) (eTable 1 in Supplement 2). Sensitivity analyses were performed (eTable 2 in Supplement 2), making a series of assumptions about missing data. We did not identify any safety concerns.

## Discussion

We designed this trial to enhance mercaptopurine adherence in children with ALL in response to the observed high prevalence of suboptimal mercaptopurine adherence and the association between mercaptopurine nonadherence and relapse risk.^6,9,10^ We focused on the implementation phase of adherence per EMERGE criteria,^7^ because sustained remission in childhood ALL depends on continued daily ingestion of mercaptopurine during maintenance, beyond initiation and before discontinuation at the end of therapy. We found that a multicomponent adherence-enhancing intervention did not result in a significantly higher proportion of patients with adherence rates 95% or higher when compared with education alone. However, exploratory analyses indicated that the intervention resulted in higher mean adherence rates in patients aged 12 years and older with baseline adherence rates less than 90%.

Previous adherence-enhancing interventions in nononcology settings have used multiple components^15^ with tailored ongoing support from allied health professionals, who often deliver intense education, professional counseling, or daily support. With the need to ensure that we could use our intervention in a multicenter setting without depending on professional support at each site, we used technologically sophisticated, yet simple, inexpensive, and easily adopted, intervention components. Importantly, the intervention components directly addressed barriers to adherence and incorporated facilitators unique to children with ALL.^9,11^ Indeed, we were able to use this intervention successfully at 59 geographically dispersed sites in a racially and ethnically diverse population.

Multimedia educational programs are useful for enhancing adherence in those with limited English proficiency, are readily accessible via Internet or DVD, and do not require training of staff to present high-quality educational messages.^16,17,18^ In our trial, we delivered education to both groups and, hence, were unable to examine the impact of education on mercaptopurine adherence. Text message reminders have been associated with improved adherence,^19^ particularly in patients with asthma,^20^ HIV,^21^ and diabetes^22^ and in kidney transplant recipients.^23^ We established personalized text message reminders, consisting of individualized messages from the treating oncologist to their patient and parent daily at the time when medication was due. Text message reminders (including mercaptopurine dose and time of administration) addressed a primary barrier to adherence in children with ALL—that is, forgetting to take mercaptopurine.^9^ DST constitutes supervision of ingestion of every dose. DST has been used successfully in patients with tuberculosis, where it reduced tubercular relapse,^24^ and led the American Thoracic Society and Centers for Disease Control and Prevention to recommend DST for all patients.^25^ We used DST to incorporate the primary facilitator to mercaptopurine adherence—that is, parental vigilance.^11^

Although this multicomponent intervention did not result in a significantly higher proportion of patients with adherence rates 95% or higher, we did make some critical observations. First, we found that there was no difference in the mean adherence rates between the intervention and education groups in the patients younger than 12 years, likely because of high baseline adherence. In contrast, the patients aged 12 years and older did benefit from the intervention, particularly where the baseline adherence rate was less than 90%. The difference in mean adherence rate between the intervention and education groups in this subgroup was 8.8%. In our previous studies,^9,10^ we found that adolescents (patients aged ≥12 years) are especially likely to have poor adherence, perhaps because of their increasing assumption of independence and decreasing parental supervision. These findings are similar to those of previous studies^26,27^ in other populations, where patients with poor adherence drew maximum benefit from adherence-enhancing interventions. These findings also emphasize the importance of targeting interventions to only those at high risk of poor adherence, ensuring efficient resource utilization. Identifying patients at risk for poor adherence can be a challenge, given that patients at highest risk of poor adherence are most likely to self-report high adherence.^28^ We have used demographic, behavioral, and mercaptopurine metabolite levels to develop a tool to identify patients at risk for poor adherence^29^ and will test this in subsequent studies.

### Limitations

The findings of this study should be interpreted in the context of certain limitations. The trial was designed to detect a significant difference in the proportion of patients with adherence rates 95% or higher between the intervention and education groups. Although the proportion of participants with adherence rates 95% or higher from baseline to postintervention period remained stable for the intervention group and decreased for the education group, we were unable to identify a significant difference in the proportion of patients with adherence 95% or higher between the 2 groups. For the younger children, the high baseline adherence rates (and the consequent ceiling effect) prevented us from examining the impact of the intervention on adherence rates. Among the older children, the impact of the intervention was most evident among those with baseline nonadherence. It is possible that baseline adherence could have been overestimated, given that initiation of electronic monitoring in itself has been shown to improve adherence.^30^ We have previously observed a decrease in adherence of 3.2%^9^ to 4.5%^10^ over the 6 months of electronic monitoring in children with ALL; this may have mitigated the effect of the intervention. Here, we found that patients in the intervention group did not demonstrate a decrease in adherence, whereas those in the education group did show a decrease. Future interventions to improve mercaptopurine adherence in childhood ALL should target the entire maintenance period and will need additional reinforcements to prevent the decline in adherence. In addition, although we have established the infrastructure for follow-up of patients for disease relapse, we have not followed the cohort long enough for describing this outcome. These limitations notwithstanding, to our knowledge, this study is the first large randomized trial in pediatric oncology that addresses a clinically relevant problem, where more than 40% of patients aged 12 years and older are at risk for ALL relapse because of suboptimal mercaptopurine adherence.^9,10^

## Conclusions

We were unable to demonstrate that this multicomponent intervention was efficacious in meeting the primary end points. However, the intervention resulted in higher adherence rates in patients aged 12 years and older with baseline adherence less than 90%. These findings inform the next steps to enhance the efficacy of the intervention— that is, refinement in patient selection by including those at risk for poor adherence.

## Notes

Supplement 1. **Trial Protocol**

Supplement 2. **eAppendix 1. **Participating Children’s Oncology Group Institutions **eAppendix 2.** Supplemental Methods **eTable 1. **Median Pre- to Post-Intervention Adherence Rates by Study Arm **eTable 2.** Sensitivity Analysis

Supplement 3. **Data Sharing Statement**
