# Supplementary material for: Effect of a Daily Text Messaging and Directly Supervised Therapy Intervention on Oral Mercaptopurine Adherence in Children With Acute Lymphoblastic Leukemia: A Randomized Clinical Trial
Source: JAMA Netw Open. 2020 Aug 27;3(8):e2014205. doi: 10.1001/jamanetworkopen.2020.14205 (PMC7453312; doi:10.1001/jamanetworkopen.2020.14205)
Supplement: Supplement 1. — Trial Protocol [file jamanetwopen-3-e2014205-s001.pdf]

STUDY PROTOCOL:

CHILDREN'S ONCOLOGY GROUP ACCL1033

A Comprehensive Approach to Improve Medication Adherence in Pediatric ALL

(Including Amendment Summary)

Smita Bhatia, MD - Chair

Activated: 02/21/12
Closed: 08/13/2018

Version Date: 04/21/15
Amendment #3

CHILDREN'S ONCOLOGY GROUP

ACCL1033

A Comprehensive Approach to Improve Medication Adherence in Pediatric ALL

A Group-wide Non-Therapeutic Study

THIS PROTOCOL IS FOR RESEARCH PURPOSES ONLY, AND SHOULD NOT BE COPIED, REDISTRIBUTED OR USED FOR ANY OTHER PURPOSE.

STUDY CHAIR

Smita Bhatia, MD, MPH
Professor, Division of Pediatric Hematology/ Oncology,
Vice Chair, Dept of Pediatrics
Director, Institute of Cancer Outcomes and Survivorship
School of Medicine, University of Alabama at Birmingham
Associate Director, UAB Comprehensive Cancer Center
Children's Hospital of Alabama
1600 7th Avenue South, Lowder 500
Birmingham, AL 35233
Phone: (205) 638-2120
Fax: (205) 638-2121
E-mail: sbhatia@peds.uab.edu

TABLE OF CONTENTS

SECTION			PAGE
[redacted]			[redacted]
ABSTRACT			5
EXPERIMENTAL DESIGN SCHEMA			7
1.0	SPECIFIC AIMS		7
[redacted]			
3.0	ENROLLMENT PROCEDURES AND ELIGIBILITY CRITERIA		14
	3.1	Study Enrollment	14
	3.2	Patient Eligibility Criteria	15
4.0	MATERIALS AND METHODS		16
	4.1	Required Procedures	16
	4.2	Additional Data	25
	4.3	Specimen Collection and Submission	27
	4.4	Laboratory Analysis	27
	4.5	Follow-Up Data Collection	27
5.0	STATISTICAL CONSIDERATIONS		28
	5.1	Statistical Design	28
	5.2	Accrual (and Expected Duration of Accrual)	28
	5.3	Analytic Plan	28
	5.4	Power and Sample Size	30
	5.5	Gender and Minority Accrual Estimates	31
6.0	DATA SAFETY AND MONITORING		31
APPENDIX I: YOUTH AND PARENT/CAREGIVER INFORMATION SHEETS			32
APPENDIX II: DATA SUBMISSION REQUIREMENTS FOR ACCL1033			37
APPENDIX III: SPECIMEN REQUIREMENTS FOR ACCL1033			39
REFERENCES			40
SAMPLE INFORMED CONSENT/PARENTAL PERMISSION FOR PARTICIPATION IN RESEARCH			43

[redacted]

[redacted]

[redacted]

[redacted]

[redacted]

[redacted]

[redacted]

[redacted]

[redacted]

[redacted]

[redacted]

[redacted]

[redacted]

[redacted]

[redacted]

The Children's Oncology Group has received a Certificate of Confidentiality from the federal government, which will help us protect the privacy of our research subjects. The Certificate protects against the involuntary release of information about your subjects collected during the course of our covered studies. The researchers involved in the studies cannot be forced to disclose the identity or any information collected in the study in any legal proceedings at the federal, state, or local level, regardless of whether they are criminal, administrative, or legislative proceedings. However, the subject or the researcher may choose to voluntarily disclose the protected information under certain circumstances. For example, if the subject or his/her guardian requests the release of information in writing, the Certificate does not protect against that voluntary disclosure. Furthermore, federal agencies may review our records under limited circumstances, such as a DHHS request for information for an audit or program evaluation or an FDA request under the Food, Drug and Cosmetics Act. The Certificate of Confidentiality will not protect against mandatory disclosure by the researchers of information on suspected child abuse, reportable communicable diseases, and/or possible threat of harm to self or others.

ABSTRACT

Acute lymphoblastic leukemia (ALL) is the most common childhood malignancy; contemporary therapy has resulted in 5-year survival rates exceeding 85%. However, this success is not enjoyed equally by all. There is evidence that Hispanics and African-Americans have significantly inferior survival outcomes when compared with non-Hispanic whites – a difference not explained by disease biology.[1] Treatment of ALL requires a maintenance phase of ~24 months composed of daily oral self-administration of a chemotherapeutic agent, 6-Mercaptopurine (6MP), in order to achieve durable remissions. There is convincing evidence that low systemic exposure to oral 6MP during maintenance adversely affects prognosis,[2,3] and low red blood cell (RBC) levels of its major metabolite (thioguanine nucleotide [6TGN]) correlate with relapse.[4] However, significant inter-patient variability exists in RBC 6TGN levels,[5] due to non-adherence to prescribed 6MP, or to inherited differences in thiopurine methyltransferase TPMT activity.

Non-adherence poses a problem in 10% to 33% of children with ALL,[5-7] and lack of adherence to oral 6MP could potentially explain the inferior outcomes observed in the vulnerable sub-populations (by race/ ethnicity and age). We have tested this hypothesis in a Children's Oncology Group (COG) study (AALL03N1, PI: Bhatia), where we measured adherence to 6MP for 6 months in children with ALL from four racial/ethnic backgrounds (Hispanics, non-Hispanic whites, African-Americans, and Asians), using the following adherence assessments: i) serial RBC 6TGN levels; ii) electronic pill monitoring system (Medication Event Monitoring Systems [MEMS]); and iii) self-report. We have completed enrollment for this study, and preliminary results are presented here. 462 patients (168 Hispanics; 157 non-Hispanic whites; 69 Asians; 68 African Americans) yielded 76,055 person-days of adherence data. Median age at participation was 6 years (2-20); 67% were males; 40% had high-risk disease per NCI criteria; 61% reported income <$50k/y; 14% reported single-caregiver households. Among patients with normal TPMT activity, each 1% increase in MEMS-based adherence was accompanied by a 14 unit (pmol/$8 \cdot 10^8$ red cells) increase in TGN (p=0.01). Multivariate longitudinal analysis revealed adherence to be significantly lower in adolescents (≥12y: 84.5% vs. <12y: 92.6%, p=0.0003); patients from single-caregiver households (87.2% vs. 92.0%, p=0.03); patients with low income (<$50k/year: 89.4% vs. ≥$50k/y: 93.8%, p=0.02); and Hispanics (90.5±1.6%), Asians (85.3±3.7%) and African Americans (85.3±2.9%) compared with non-Hispanic whites (95.3±1.2%, p<0.0001). Reasons for missing 6MP included forgetfulness (79%), logistical barriers (19%), and active refusal (2%). After a median follow-up of 5.4 years, multivariate analysis (adjusting for clinical/sociodemographic factors) revealed that adherence <95% was associated with an increase in relapse risk (reference: adherence ≥95%; 94.9%-90%: Hazard Ratio [HR]=3.3, 95% Confidence Interval [CI], 1.0-11.6, p=0.06; 89.9%-85%: HR=3.4,

95%CI, 0.9-13.0, p=0.07; <85%: HR=4.5, 95%CI, 1.3-15.1, p=0.02), leading us to use <95% as the cut-point for adherence with a clinically unacceptable increase in relapse. Using this definition, 45% of the patients were non-adherers. The cumulative incidence of relapse was significantly higher among non-adherers (18.8% vs. 4.9%, p=0.0003). Furthermore, non-adherers were at a 3.7-fold increased risk of relapse (95%CI, 1.4-10.2, p=0.01), after adjusting for sociodemographic/clinical variables. The adjusted risk of relapse attributable to non-adherence was 47% for this cohort that had entered maintenance in first clinical remission.[8]

Study results suggest that lower adherence to oral 6MP influences risk of relapse, and contributes to the ethnic differences in disease outcome observed in children with ALL. Results from this study highlight the need to develop effective interventions to improve adherence targeted at populations at high risk. These interventions need to take into account the following: i) asymptomatic nature of this potentially lethal disease during maintenance (and hence low motivation or cues to remain adherent); ii) once-daily 6MP dosing schedule (a relatively simple schedule); iii) prolonged, yet finite duration of treatment (~24 months – and therefore a less critical need to institute behavioral modifications usually needed for adherence to lifelong medications); and iv) barriers/facilitators to adherence unique to this population. There is a critical need to study the efficacy of a comprehensive adherence-enhancing intervention program that is informed by perceived barriers to adherence experienced by those at high risk. This investigation will test the feasibility, utility, and efficacy of a technologically sophisticated, web-based medication scheduling and text-messaging reminder system (REM) that capitalizes on the ubiquitous presence and acceptance of personal cellular phones, and prompts directly supervised therapy (DST) of each dose by a designated parent/caregiver, coupled with a multimedia-based interactive patient education program (MIPE), to increase adherence to daily oral 6MP in children with ALL.

EXPERIMENTAL DESIGN SCHEMA

4 to 8 weeks prior to Day 1 →

- Identify eligible study subjects, explain nature of study, obtain informed consent/assent
- Dispense Patient Supply Kit (containing MEMS® medication bottles, standard child resistant caps, and instructions for pharmacist) to patient. At next scheduled refill prior to Day 1, pharmacy of patient's choice dispenses new supply of 6MP into MEMS® medication bottle
- Request MEMS® TrackCap™ and Depression questionnaires from Coordinating Center

Day 1 →

- CRA procures blood sample and ships to St. Jude Clinical Pharmacokinetics Laboratory
- CRA gives MEMS® TrackCap™ to patient and instructs patient to start using MEMS® TrackCap™; CRA follows up with patient or parent/caregiver by phone on next business day to confirm that MEMS® TrackCap™ is being used
- Parent/caregiver (<u>or</u> patient ≥18 years) complete Demographic Questionnaire
- Parent/caregiver <u>and</u> patient (if ≥12 years) complete Comprehensive Adherence Questionnaire
- Parent/caregiver <u>and</u> patient (if ≥12 years) complete Acculturation Questionnaire (**only Hispanic and Asian participants**)
- Parent/caregiver <u>and</u> patient (if ≥12 years) complete Depression Questionnaire (BDI-II and/or CDI 2)
- CRA enrolls patient on study via eRDE. Randomization into the Education Only (EDU) or Intervention Program (IP) arm will be automatically assigned
- CRA will complete OnStudy eRDE form on following business day, after confirming that MEMS® TrackCap™ is being used

Day 29 →

- CRA procures blood sample and ships to St. Jude Clinical Pharmacokinetics Laboratory
- Parent/caregiver <u>and</u> patient (if ≥12 years) complete Abbreviated Adherence Questionnaire
- Designated parent/ caregiver and patient (if ≥12 years) view the Multimedia Interactive Patient Education program (MIPE); CRA documents completion of MIPE on Day 29 Documentation Form

IP Patients Only:

- CRA issues cell phone(s) to parent/caregiver and patient aged 12 years and older (if needed)
- Healthcare provider gives customized electronic 6MP schedule to patient
- Healthcare provider activates automated customized SMS text messaging reminders (REM) to be delivered via cellular phone according to the patient's electronic 6MP schedule; if any changes to 6MP schedule are required between visits, healthcare provider updates customized SMS text messaging reminder via web-based interface
- CRA and healthcare provider instruct designated parent/caregiver and patient (if ≥12 years) to use the text message as a cue that the 6MP is due; and for the parent/caregiver to supervise the patient taking the dose (DST); and to confirm via text message that the dose has been taken
- CRA documents the provision of standardized instructions for REM and DST on the Day 29 Documentation Form and submits form to Coordinating Center (Children's of Alabama/UAB)

Day 57 →
Day 85 →
Day 113 →

- CRA procures blood sample and ships to St. Jude Clinical Pharmacokinetics Laboratory
- Parent/caregiver <u>and</u> patient (if ≥12 years) complete Adherence Questionnaire (comprehensive version on Day 57; abbreviated version on Days 85 and 113)
- **Day 85 ONLY:** Designated parent/ caregiver completes the Parental Distress Questionnaire (PIP)

IP Patients Only:

- Healthcare provider gives customized electronic 6MP schedule to patient
- Healthcare provider activates automated customized SMS text messaging reminders to be delivered via cellular phone according to the patient's electronic 6MP schedule; if any changes to 6MP schedule are required between visits, healthcare provider updates customized SMS text messaging reminder via web-based interface
- CRA and healthcare provider reinforce instructions to the patient (if ≥12 years) and parent/designated caregiver to use the text message as a cue that the 6MP is due and for the caregiver to supervise the patient taking the dose (DST); and to confirm via text message that the dose has been taken

Day 141 →

- CRA procures blood sample and ships to St. Jude Clinical Pharmacokinetics Laboratory
- Parent/caregiver <u>and</u> patient (if ≥12 years) complete Comprehensive Adherence Questionnaire
- Parent/caregiver <u>and</u> patient (if ≥12 years) complete Intervention Rating Questionnaire (IP only) or MIPE Rating Questionnaire (EDU only)
- Patient returns M<u>EMS</u>® *TrackCap*™ <u>to</u> CRA during clinic visit
- CRA mails the MEMS® *TrackCap*™ <u>in padded envelope</u> to the Coordinating Center

IP Patients Only

- Parent/caregiver and patient return cell phone(s) to institutional CRA during clinic visit*
- CRA mails cell phone(s) <u>in padded envelope</u> to Coordinating Center*

* Only if study cell phone(s) used by parent/caregiver and/or patient

1.0 SPECIFIC AIMS

Conduct a randomized trial of an intervention program (IP) consisting of customized printed 6MP schedules and electronic reminders (cellular text messages) (REM) coupled with directly supervised therapy (DST) and multimedia-based interactive patient education program (MIPE) vs. education alone (EDU) in children diagnosed with ALL. For children assigned to IP who are <12 years of age at randomization, only the designated caregiver will receive text message reminders, prompting DST. For older patients (≥12 years), both the patient and designated parent/caregiver will receive text message reminders, prompting DST.

Primary Aim: Determine the impact of interventions proposed in IP vs. EDU on adherence to oral 6MP in children with ALL. Adherence will be measured by: i) MEMS (primary measure of adherence to oral 6MP, providing real-time data; ii) red cell TGN levels (providing data on chronic, systemic 6MP exposure)

Hypothesis 1.1: An intervention package consisting of customized printed schedules, text message reminders, DST and education (IP) will result in a higher proportion of individuals with adherence rates ≥95% compared with education alone (EDU)

Hypothesis 1.2: A higher proportion of patients placed on IP will have adherence rates ≥95% compared with those placed on EDU, irrespective of age at study participation (<12 or ≥12 years), thus taking into account age-appropriate modifications in intervention (text message reminders sent only to parents of younger patients)

Secondary Aims

Secondary Aim 1: Examine the modifying effect of sociodemographic and psychosocial variables, and the mediating effect of health beliefs/ knowledge on change in adherence with intervention

Hypothesis S1: Certain subgroups will derive more benefit from IP (Table 1: Modifiers): adolescents; racial/ ethnic minority patients; patients from single parent households; parents/patients with absence of depressive symptoms; parents with low levels of distress; and families with low acculturation levels. Changes in adherence with MIPE will be mediated (for both IP and EDU) by changes in health knowledge/ beliefs (Table 1: Mediators): perceived severity of illness, knowledge regarding purpose of 6MP, perception of self-efficacy to overcome barriers. The impact of MIPE as a mediator will be stronger in IP arm because of the additional behavioral reinforcement by text message reminders and DST

Table 1. Modifiers and mediators of intervention on change in adherence

**Modifiers**	**Rationale**
≥12 years of age	Lack of parental supervision contributes to non-adherence, more likely to forget; more likely to respond to intervention
Racial/ ethnic minorities	SES/ linguistic issues create barriers to access; influence understanding of treatment; more likely to respond to intervention
Single parent household	Lack of supervision contributes to non-adherence, more likely to forget; more likely to respond to intervention
Depressive symptoms in parent/ child	Depressive symptoms impair cognitive focus, energy, motivation; less likely to respond to intervention
Parental distress	Parental distress may impair focus, and affect patients'/ parents' ability to follow instructions less likely to respond to intervention
Low levels of acculturation	Impaired understanding re role of 6MP; lack of communication with healthcare system; more likely to respond to intervention
**Mediators**	**Rationale**
Underestimation of severity of illness	Change in knowledge re severity of illness/ likelihood of relapse if 6MP not taken as prescribed will help improve adherence

Lack of knowledge re purpose of 6MP	Change in understanding regarding how 6MP works in preventing relapses will help improve adherence
Low self-efficacy to overcome barriers	Improvement in confidence in the ability to carry out instructions will help improve adherence

Secondary Aim 2: Determine impact of IP vs. EDU on risk of relapse of ALL

Hypothesis S2: IP will result in lower risk of relapse compared with EDU – upon mature follow-up of the two arms

[redacted]

[redacted]

[redacted]

[redacted]

[redacted]

[redacted]

[redacted]

[redacted]

[redacted] [redacted]

[redacted]

[redacted] [redacted]

[redacted]

[redacted]

[redacted]	[redacted]	[redacted]
[redacted]	[redacted]	[redacted]
[redacted]	[redacted]	[redacted]
[redacted]	[redacted]	[redacted]
[redacted]	[redacted]	[redacted]
[redacted]	[redacted]	[redacted]

[redacted]

[redacted] [redacted]

[redacted]

[redacted]

[redacted]

[redacted]

[redacted]

[redacted]

[redacted]

[redacted]

[redacted]

[redacted]

[redacted]

[redacted]

[redacted]

[redacted]

[redacted]

[redacted]

[redacted]

[redacted] [redacted]

[redacted]

[redacted] [redacted]

[redacted]

[redacted] [redacted]

[redacted]

[redacted]

[redacted]

[redacted]

3.0 ENROLLMENT PROCEDURES AND ELIGIBILITY CRITERIA

3.1 Study Enrollment

3.1.1 Patient Registration

Prior to enrollment on this study, patients must be assigned a COG patient ID number. This number is obtained via the eRDE system once authorization for the release of protected health information (PHI) has been obtained. The COG patient ID number is used to identify the patient in all future interactions with COG. If you have problems with the registration, please refer to the online help.

3.1.2 IRB Approval

Local IRB/REB approval of this study must be obtained by a site prior to enrolling patients. Sites must submit IRB/REB approvals to the NCI's Cancer Trials Support Unit (CTSU) Regulatory Office and allow 3 business days for processing. The submission must include a fax coversheet (or optional CTSU IRB Transmittal Sheet) and the IRB approval document(s). The CTSU IRB Certification Form may be submitted in lieu of the signed IRB approval letter. All CTSU forms can be located on the CTSU web

page (https://www.ctsu.org). Any other regulatory documents needed for access to the study enrollment screens will be listed for the study on the CTSU Member's Website under the RSS Tab.

IRB/REB approval documents may be faxed (1-215-569-0206),
Emailed (CTSURegulatory@ctsu.coccg.org) or mailed to the CTSU Regulatory office.

When a site has a pending patient enrollment within the next 24 hours, this is considered a "Time of Need" registration. For Time of Need registrations, in addition to marking your submissions as 'URGENT' and faxing the regulatory documents, call the CTSU Regulatory Helpdesk at: 1-866-651-CTSU. For general (non-regulatory) questions call the CTSU General Helpdesk at: 1-888-823-5923.

3.1.3 Study Enrollment

Patients may be enrolled on the study once all eligibility requirements for the study have been met. Study enrollment is accomplished by going to the Enrollment application in the eRDE system. If you have problems with enrollment, refer to the online help in the Applications area of the COG website. **Patients must NOT be enrolled until they have their 6MP dispensed in the MEMS® medication bottle (with a standard child resistant cap fitted to the MEMs bottle).** Please see Section 4.1.1 for additional details regarding patient identification and recruitment.

3.1.4 Randomization

Randomization, stratified by ethnic background (4 strata: Hispanic, non-Hispanic white, African-American, and Asian/Other) and age at randomization (2 strata: < 12 years, 12 years and older), will take place at the time a patient is enrolled on study via the Eligibility eRDE Form. Patients will be assigned to either the Intervention Program (IP) arm or Education Only (EDU) arm (see Section 5.1 for details).

3.2 Patient Eligibility Criteria

Important note: The eligibility criteria listed below are interpreted literally and cannot be waived (per COG policy posted 5/11/01). All clinical and laboratory data required for determining eligibility of a patient enrolled on this trial must be available in the patient's medical/research record which will serve as the source document for verification at the time of audit.

3.2.1 Inclusion Criteria

(1) Diagnosis of ALL at ≥ 1 year and ≤ 21 years of age, in first remission. Enrollment on a COG therapeutic study for ALL is not required.

(2) At the time of enrollment, patient must have completed at least 24 weeks of maintenance chemotherapy, and is scheduled to receive at least 24 more weeks of maintenance chemotherapy

(3) Receiving continuous oral 6MP during the maintenance phase of therapy for ALL (held only for toxicity or illness), and will be returning to the clinic every 4 weeks for scheduled appointments while enrolled on COG ACCL1033 (between Days 1 and 141).

(4) Has a designated parent or caregiver who is willing to enter into a mutual agreement with the patient to participate in a daily supervised medication administration routine

(5) Able and willing to use the MEMS® *TrackCap*™ (e.g., not using a pillbox or prescribed liquid 6MP)

(6) Parent/caregiver and patient (if 12 years and older) must be willing to use a cellular telephone to receive medication reminders via text messaging during study period

(7) Patient and parent/caregiver must speak English or Spanish

3.2.2 Exclusion Criteria

(1) Patients with Down syndrome (due to excessive toxicity during Maintenance on historical trials, patients with Down syndrome now receive reduced Maintenance duration and vincristine/steroid

pulses frequency [every 12 weeks rather than every 4 weeks]; thus Maintenance therapy for Down syndrome patients is not comparable to standard Maintenance therapy for high-risk ALL)

(2) Patients who previously participated in or are currently participating in another intervention clinical trial designed to improve adherence

3.2.3 Regulatory

3.2.3.1
All patients and/or their parents or legal guardians must sign a written informed consent.

3.2.3.2
All institutional, FDA, and NCI requirements for human studies must be met.

4.0 MATERIALS AND METHODS

4.1 Required Procedures

Each requirement is described in detail below (see Section 4.2, Table 5 for study schema).

4.1.1 Patient Identification and Enrollment

COG institutions will prospectively identify all patients meeting eligibility criteria. Patients will be recruited during the maintenance phase of ALL therapy. All eligible patients and their designated parents/caregivers will be approached before study Day 1 (we suggest approaching 4 to 8 weeks prior to the expected Day 1 appointment), the study explained to them, and informed consent/assent will be obtained. A separate consent is not required from the designated parent/ caregiver; however, the parent/ caregiver will be given the Parent/Caregiver Information Sheet (Appendix I) to review prior to obtaining the study consent/assent. Day 1 will be defined as a scheduled appointment for maintenance chemotherapy (usually a monthly appointment for Vincristine) occurring after the patient has completed at least 24 previous weeks of maintenance chemotherapy. In order to detect any systematic differences between participants and non-participants, institutions will collect a limited set of demographic variables (age at diagnosis, time from diagnosis, current age, gender, preferred language, race/ethnicity, and reason given for study refusal) from all eligible patients who choose not to participate in the study. The coordinating center, Children's of Alabama/University of Alabama at Birmingham (UAB), will request submission of a Study Participation Worksheet every 3 months, which will include the following information: the number of patients eligible, the number of patients approached, and the number of patients who refused since the institution's last report. For each refusal, CRAs will complete a Study Refusal form and submit to the coordinating center. Parents/caregivers and patients will be informed that the major goals of the study are to institute mechanisms to improve adherence to daily oral 6MP; that all parents/caregivers and patients ≥ age 12 will watch an educational video; and that some of the patients will receive a system of reminders for oral 6MP, and a designated parent/ caregiver will supervise the intake of the drug – while others will continue to take their medications as per their usual routine, in addition to having watched the educational video (i.e., all parents and patients ≥ age 12). In order to address the needs of the Spanish-speaking patient population, the study consent, questionnaires, and instructional materials relevant to the MEMS® medication bottles, *TrackCap*™, and intervention, have been translated into Spanish.

At the time informed consent is obtained, the Clinical Research Assistant (CRA) will distribute the Patient Supply Kit to the patient so their next 6MP refill will be dispensed into the MEMS® medication bottle. The Patient Supply Kit contains a supply of empty MEMS® medication bottles with standard child resistant caps and written instructions for the patient and pharmacist. Once informed consent is obtained, the CRA can request a MEMS® *TrackCap*™ from the coordinating center (Children's of Alabama/UAB,

205-638-2129; AdherenceStudy@peds.uab.edu). The coordinating center will also mail the BDI-II and CDI 2 depression questionnaires that will be completed on study Day 1.

The patient will be instructed to have their next scheduled 6MP refill dispensed into the MEMS® medication bottle and will use the standard child resistant cap until study Day 1.

Patients should be enrolled via the COG eRDE system on (and not prior to) Day 1 of this study. By Day 1 of the study, the patient must have their 6MP dispensed in the MEMS® medication bottle (with a standard child resistant cap fitted to the MEMs bottle). At the Day 1 appointment, the CRA will assign a MEMS®*TrackCap™* to the patient and instruct the patient and parent/caregiver to start using the MEMS® *TrackCap™* when taking each prescribed dose of 6MP throughout the study period. The site CRA will follow-up by phone with the parent/caregiver on the next business day following enrollment, to confirm that the *TrackCap™* is being used or if not, to identify obstacles to *TrackCap™* use and determine solutions. The site CRA will contact the coordinating center by phone (Children's of Alabama/UAB, 205-638-2129) or email (AdherenceStudy@peds.uab.edu) for assistance as needed regarding any obstacle to *TrackCap™* use that cannot be resolved at the institutional level.

On Day 1, the CRA will complete the Eligibility eRDE Form, and the eRDE system will automatically randomize participants to a study assignment (Intervention Program [IP] or Education Only [EDU]). CRAs will need to enroll the patient in the correct stratum, using the self-reported race/ethnicity of the patient (African-American, Caucasian, Hispanic, or Asian/Other; **as reported on the Demographics Questionnaire**) and age of the patient (< 12 years, ≥ 12 years as of day of enrollment). Definitions of the race/ethnicities are listed below:

African-American:
Includes patients who are African-American or of sub-Saharan black African ancestry

Caucasian:
Includes white or light-skinned patients of European, North African, or Middle Eastern ancestry

Hispanic:
Patients of Hispanic ethnicity, including the following: Mexican, Mexican American, Chicano, Cuban, Puerto Rican, or Other Spanish / Hispanic / Latino ethnicities

Asian/Other:

Asian**:**
Patients of Asian ancestry, including the following: Asian Indian (subcontinent), Chinese, Japanese, Korean, Native Hawaiian, Guamanian or Chamorro, Pacific Islander, Filipino, Vietnamese, Samoan, Hmong, Cambodian, Thai, Laotian, or Other Asian races

Other**:**
Patients of who do not self-report race/ethnicity in the categories listed above or who select two of the race/ethnicity categories listed above (multi-ethnic/multi-racial, Native American, etc.)

On the next business day following enrollment, the CRA will contact the designated parent/caregiver to confirm the use of the TrackCap. If the CRA is unable to contact the designated parent/caregiver the following day, the CRA will continue calling daily until contact is made. After calling to confirm use of the TrackCap, the CRA will complete the OnStudy eRDE form, which will document the following:

- The designated parent/caregiver who is willing to enter into a mutual agreement with the patient to participate in a daily supervised medication administration routine (e.g. "Mother", "Father," "Grandmother") as described in Section 4.1.5.2.3. For patients who travel between

more than one household, only one designated parent/ caregiver (usually the parent/caregiver with whom the child spends the majority of his/her time) will be identified.

- Whether cellular telephones will need to be assigned to the designated parent/caregiver and/or patient (if ≥ 12 years).
- The language preference (English or Spanish) of the designated parent/caregiver and patient (if ≥ 12 years),

Participants should NOT be notified of their randomization until Day 29. The first 28 days of the trial will constitute an observational or "break-in" period – when all study participants (irrespective of the randomization arm) will receive their medications from the MEMS® medication bottle. The adherence rate for Days 1 - 28 will serve as the baseline adherence measure. The intervention will begin on Day 29.

As an enrollment incentive, COG will be reimbursing all IRB-approved sites $2000 (1.0 cancer control credit) per patient registered on ACCL1033.

4.1.2 Blood Samples
A 5 ml aliquot of blood will be obtained on Day 1 for TPMT phenotyping and on Days 29, 57, 85, 113, and 141 of the study for 6TGN levels. The specific days correspond to the days in the maintenance cycle when patients return to the clinic for intravenous vincristine and steroid pulses. If the TPMT assay cannot be performed using any of the blood samples collected during the study, the coordinating center will contact the institutional CRA to request the collection of an additional 5 ml blood sample. See Section 4.3 for additional blood sample collection and shipping instructions.

4.1.3 Questionnaires
Prior to administration of the questionnaires, the responsible investigators at the institutions will determine if the patient and/or parent/caregiver needs a Spanish version of the questionnaire, as well as whether the questionnaire needs to be administered by an interviewer rather than being self-administered. All questionnaires have been translated into Spanish in order to address the needs of the Spanish-speaking study participants. See Table 3 for the questionnaire collection schedule.

4.1.3.1 Demographic Questionnaire
A brief Demographic Questionnaire will be completed by the designated parent/ caregiver (or patient if 18 years of age or older) on Day 1, and includes information related to self-reported race/ethnicity, socioeconomic status, and family structure. Estimated completion time is 5 minutes.

4.1.3.2 Adherence Questionnaire
The designated parent/caregiver and patient (if ≥ 12 years) will be asked to complete a comprehensive Adherence Questionnaire during regularly scheduled clinic visits on Days 1, 57, and 141 of the study in order to determine baseline knowledge and adherence-related beliefs and behaviors, and to document changes (if any) in knowledge and adherence-related beliefs and behaviors over the course of the study. The questionnaire is based on the Health Belief and Self Efficacy Models and is designed to elicit the behavioral determinants of adherence. The Adherence Questionnaire elicits self-assessment of adherence and reasons for missed doses (if any) [perceived barriers]; patient level of responsibility [self-efficacy]; current health status [perceived vulnerability/susceptibility]; use of MEMS cap; knowledge of disease/treatment regimen [perceived severity, susceptibility, benefits]; medication burden, barriers to medication-taking [perceived barriers]; adherence self-efficacy [self-efficacy]; family relationships [social support]; and social desirability. All items will be collected from participants on both study arms (IP and EDU) at baseline (Day 1), at 4 weeks following implementation of the intervention (Day 57), and at completion of the study (Day 141) to allow comparison of the questionnaire data before and after all patients view MIPE and before and after the IP arm experiences the text messaging and DST portion of the intervention. Estimated completion time is 15 minutes. An abbreviated Adherence Questionnaire, including only those questions relevant to the

number of days that the patient did and did not take 6MP over the past month, reasons for missed doses (if any), patient level of responsibility, change in health status; and use of MEMS cap, will be administered on Days 29, 85, and 113 of the study. Estimated completion time is 5 minutes.

4.1.3.3 Intervention Rating Questionnaire (IP only)
On Day 141, patients (if ≥ 12 years) and parents/caregivers assigned to the IP arm will be asked to complete a brief Intervention Rating Questionnaire to rate their perceptions regarding the helpfulness of the MIPE program, cellular text message reminders (REM), and DST. Estimated completion time is 5 minutes.

4.1.3.4 MIPE Rating Questionnaire (EDU only)
On Day 141, patients (if ≥ 12 years) and parents/caregivers assigned to the EDU arm will be asked to complete a brief MIPE Rating Questionnaire to rate their perceptions regarding the helpfulness of the MIPE program. Estimated completion time is 5 minutes.

4.1.3.5 Acculturation Questionnaire (Hispanic and Asian participants only)
Acculturation questionnaires will be collected from Hispanic and Asian participants. Hispanic patients (if ≥ 12 years) and their parents/caregivers will be asked to complete a Hispanic Acculturation Questionnaire on Day 1 in order to determine the level of acculturation of Hispanic participants. The Hispanic Acculturation Questionnaire[40] consists of 12 items scaled on a 5-point Likert format that measure three dimensions: Language Use (5 items), Media Factor (3 items), and Ethnic Social Relations Factor (4 items). A score of 1 indicates a low level of acculturation and 5 a high level of acculturation. Estimated completion time is 5 minutes.

Asian patients (if ≥ 12 years) and their parents/caregivers will be asked to complete the Suinn-Lew Asian Self Identity (SL-Asia) Acculturation Questionnaire on Day 1 in order to determine the level of acculturation of Asian participants. The SL-Asia Acculturation Questionnaire[24] consists of 26 items (multiple choice and 5-point Likert format) that ask about the participant's historical background as well as recent behaviors that may be related to cultural identity. A score of 1 indicates a low level of acculturation and 5 a high level of acculturation. Estimated completion time is 10 minutes.

4.1.3.6 Depression Questionnaires
The designated parent/caregiver and patient (if ≥ 12 years) will be asked to complete a questionnaire to measure depression during their regularly scheduled clinic visit on Study Day 1. Depressive symptoms (and not anxiety) are associated with poor adherence;[25] depressive symptoms may impair cognitive focus, energy, motivation, and affect the patients'/parents' ability to follow instructions. The designated parent/caregiver and patients 18 years and older will complete the Beck Depression Inventory-II (BDI-II).[41,42] Adolescent patients (12-17 years) will complete the Children's Depression Inventory-2 (CDI 2) self-report version; and for younger patients (<12 years), the designated parent/caregiver will complete the CDI 2 parent-report version.[43] English and Spanish versions are available. Estimated completion time is 5 minutes.

Additionally, the BDI-II and CDI 2 (patient self-report version, patients 12-17 years) contain items assessing suicidal ideation. The CRAs at the participating sites will review the responses to these questions at the time of questionnaire completion, and will alert the patient's clinician immediately should the patient and/or parent's response indicate active suicidal ideation (i.e., BDI-II, Item #9 - response #2 or #3 or CDI 2 self-report, Item # 8 - response #3). The clinician will then further evaluate the patient/parent and initiate an emergent referral to psychosocial services as indicated.

The BDI-II and CDI 2 questionnaires are licensed and may not be posted in the questionnaire packet on the COG website. The coordinating center will mail these questionnaires when the MEMS® *TrackCap*™ is requested (at the time of consent).

4.1.3.7 Parental Distress Questionnaires

The designated parent/caregiver will be asked to complete a questionnaire to measure parental distress during their regularly scheduled clinic visit on Study Day 85. Parental distress may play a role in adherence. We will measure parental distress using the Pediatric Inventory for Parents (PIP)[26] – a parenting burden measure for parents of children with cancer/other chronic illnesses. It captures parental distress in 4 domains (Communication, Emotional Distress, Medical Care, Role Function), and has also been validated in Spanish.[44] Estimated completion time is 10 minutes.

Table 3. Questionnaire collection schedule for patient* and parent/caregiver

	Study Day					
	**Day 1**	**Day 29**	**Day 57**	**Day 85**	**Day 113**	**Day 141**
**Demographic Questionnaire (Designated Parent/Caregiver; patients>18 may complete if designated caregiver unaware of patient demographics)**						
IP and EDU arms	√					
**Acculturation Questionnaire (Asian /Hispanic Patients* and Parents/Caregivers only)**						
IP and EDU arms	√					
**BDI-II Depression Questionnaire (Designated Parent/Caregiver and Patients 18 years and older)**						
IP and EDU arms	√					
**CDI 2 Depression Questionnaire (self-report for patients 12-17 years; parent report for patients younger than 12 years)**						
IP and EDU arms	√					
**Version of Patient* and Parent/Caregiver Adherence Questionnaire**						
Comprehensive Version (IP and EDU arms)	√		√			√
Abbreviated Version (IP and EDU arms)		√		√	√	
**Parental Distress (PIP) Questionnaire (Designated Parent/Caregiver only)**						
IP and EDU arms				√		
**Intervention Rating Questionnaire (Patient* and Parent/Caregiver)**						
IP arm only						√
**MIPE Rating Questionnaire (Patient* and Parent/Caregiver)**						
EDU arm only						√

* Patients only complete the questionnaires if they are 12 years or older.

4.1.4 Electronic Medication Monitoring (MEMS)

We will use the MEMS monitoring device, which employs microelectronic technology to record actual medication vial openings (date and time) as a measure of dosing. A patient supply kit containing empty MEMS® child resistant medication bottles and child resistant standard caps will be dispensed to study participants after informed consent is obtained (before Study Day 1). The patient will receive written instructions directing them to provide the empty MEMS® medication bottle and standard cap, along with their 6MP prescription, to their local pharmacist, who will fill the prescription using the MEMS® medication bottle the next time a 6MP refill is needed. The patient will be instructed that any additional

refills of 6MP required during the study period are also to be dispensed in a MEMS® medication bottle. Included with the empty MEMS® medication bottle will be instructions for the pharmacist, explaining that the patient is participating in a study monitoring adherence to the regularly prescribed 6MP medication regimen. **The patients must have their 6MP in the MEMS® medication bottle by Day 1. At the Day 1 appointment, the CRA will assign a MEMS®*TrackCap™* to the patient and instruct the patient to start using the MEMS® *TrackCap™* when taking each prescribed dose of 6MP throughout the study period. The CRA will follow-up by phone with the parent/caregiver on the next business day following enrollment, to confirm that the *TrackCap™* is being used or if not, to identify obstacles to *TrackCap™* use and determine solutions (if CRA is unable to reach the parent/caregiver on the next business day, the CRA will continue calling daily until contact is made). The CRA will contact the coordinating center by phone (Children's of Alabama/UAB, 205-638-2129) or email (AdherenceStudy@peds.uab.edu) for assistance as needed regarding any obstacle to *TrackCap™* use that cannot be resolved at the institutional level.** Patients and parents/caregivers will be informed of the function of MEMS® *TrackCap™*, and a patient instruction sheet will be provided to the families. The patients will be instructed to return the MEMS® *TrackCap*™ to the clinic on Day 141, and the CRA will mail it to the coordinating center at Children's of Alabama/UAB (see address below).

At the completion of a patient's study participation (Day 141), please mail MEMS® *TrackCap*™ in a padded envelope (provided as needed to the institutions by Children's of Alabama/UAB) to:

Dr. Smita Bhatia
UAB Division of Peds Hem/Onc
1600 7th Ave S, Lowder 500
Birmingham, AL 35233-1711
Phone: (205) 638-2129
Fax: (205) 212-3400

Data from the MEMS® *TrackCap™* will be downloaded by the coordinating center using the MEMS® communicator and software, providing the dates and times of all pill container openings.

Please contact the coordinating center (Children's of Alabama/UAB, 205-638-2129; AdherenceStudy@peds.uab.edu) to obtain patient supply kits.

Please Note: Since this is a research study, results of study blood tests and electronic medication monitoring will not be reviewed in real-time, and hence will not be available for clinical decision-making; therefore, neither patients nor their physicians will be given the results of the blood tests or electronic monitoring by the MEMS® device.

4.1.5 Intervention

The reasons why individuals are non-adherent vary; attaining high adherence rates therefore requires a multifaceted approach. We have used the results of our prior studies to inform the intervention that will be tested in the current study – the approach is summarized in Table 4.

Table 4: A multifaceted approach to address barriers to adherence identified by children with ALL or their caregivers.

**Barriers to Adherence** →	**Interventions to Address the Barriers to Adherence**
1. Lack of understanding re purpose of 6MP in leukemia treatment<br>2. Underestimation of perceived susceptibility to/ severity of illness (disease is asymptomatic in maintenance phase)<br>3. Low perception of self-efficacy to overcome barriers	**Multimedia Interactive Patient Education Program (MIPE)**<br>▪ Participants customize their learning experience based on their specific learning needs and linguistic preferences<br>▪ Content addresses health knowledge, self-efficacy and health beliefs
1. Prolonged duration of treatment<br>2. Forgetfulness<br>3. Poor communication of instructions by healthcare provider<br>4. Language barriers<br>5. Taking a passive role in treatment<br>6. Lack of parental supervision with ingestion of medication	**Web-Based Medication Scheduling Application with Integrated Text Messaging Reminder System (REM)**<br>▪ Printed 6MP instructions customized for patient in English/ Spanish<br>▪ Automated text reminders delivered to patient (if ≥12 years) ***and*** parent/ caregiver in preferred language daily at scheduled time of patient's 6MP dose<br>**Directly Supervised Therapy (DST)**<br>▪ Automated text reminders prompt patient (if ≥12 years) and parent/caregiver to engage in DST

Upon enrollment, subjects are randomized 1:1 to receive Education Only (EDU) or the Intervention Program (IP) (Figure 1). The first 28 days of the trial constitute an observational or "break-in" period – when all study participants (irrespective of the randomization arm) receive their 6MP from the MEMS® medication bottle with *TrackCap*™. The adherence rate for Days 1 - 28 serves as the baseline adherence. The actual intervention begins on Day 29 and ends on Day 140.

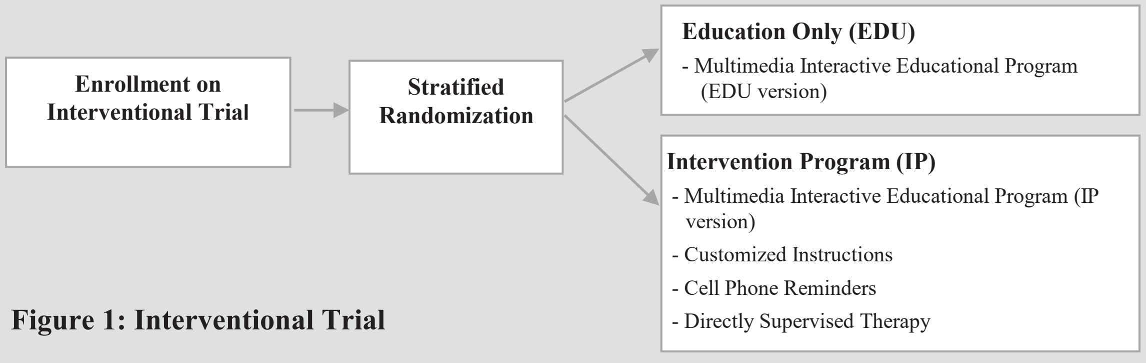

Figure 1: Interventional Trial

4.1.5.1 Education Only (EDU)

On Day 29, the designated parent/caregiver and patient (if ≥ 12 years) will view a multimedia interactive education program (MIPE – EDU version) during their scheduled clinic visit; the institutional CRA will submit documentation certifying completion of this portion of the intervention (see Day 29 Documentation Form). There are two EDU versions of the MIPE program, depending on the age of the participant: < 12 years and ≥ 12 years.

The MIPE (EDU version) program features video clips of ALL patients of various ages (and their parents/caregivers) discussing maintenance treatment. Content is accessible in English and Spanish. Participants choose the patient/parent/caregiver they most closely identify with and view a variety of video clips featuring the selected patient/parent/caregiver addressing adherence-specific content, including the purpose and importance of taking oral 6MP, health beliefs related to perceived susceptibility/severity of illness, perceived benefits of and barriers to maintenance therapy, and examples

of ways that patient/ parents have overcome barriers associated with taking oral 6MP to improve their own/their child's adherence. Video clips featuring explanations from healthcare professionals are also integrated into the program. The multimedia program provides the flexibility for participants to customize their learning experience based on their specific learning needs and linguistic preferences in a culturally-sensitive learning environment. The program, available in the clinic on-line or via DVD, takes 20 to 25 minutes to view, depending on the choices made by participants in their interaction with the program. This intervention is designed to address the clinical implications of the Health Belief and Self-Efficacy Models, in which individuals who understand the perceived benefit of a health behavior (i.e., taking oral 6MP as prescribed) are more likely to take control of that behavior (i.e., improve adherence).

Patients on the EDU arm will continue to take the 6MP from the MEMS® medication bottle and measures of adherence will be assessed in an identical fashion as for IP patients (i.e., MEMs data will be downloaded and red cell 6TGN levels will be collected; the only exception will be real-time text messaging confirmation of adherence).

4.1.5.1.1 Alternative MIPE EDU Viewing for the Designated Parent/Caregiver

The designated parent/caregiver should be encouraged to be present at the Day 29 appointment. However, if they are unable to accompany the patient to the Day 29 appointment, the CRA will provide an Alternative MIPE EDU Viewing Packet to be sent home with the patient. The Alternative MIPE Viewing Packet will include the MIPE EDU program (< 12 years or ≥ 12 years version) and a letter with instructions for watching the MIPE EDU program within 24 hours of the patient's Day 29 clinic appointment. On the next business day following the Day 29 clinic visit, the CRA will call the designated parent/caregiver to confirm that the designated parent/caregiver has watched the MIPE EDU program, and will document this on the Day 29 Documentation Form. If the MIPE EDU program has not been viewed at the time that the CRA makes the follow-up call, the CRA will ask the parent/caregiver if they agree to view the program on that day. If the parent/caregiver agrees, the CRA will call back the following day to confirm that the program has been viewed and will document this on the Day 29 Documentation Form; if the MIPE EDU program has not been viewed at the time of the second follow-up call, the CRA will document this on the Day 29 Documentation Form. If the CRA is unable to reach the designated parent/caregiver on Day 29 and/or for the second follow-up call, the CRA will continue calling daily until contact is made.

4.1.5.2 Intervention Program (IP)

4.1.5.2.1 Multimedia Interactive Patient Education (MIPE – IP version)

On Day 29, the designated parent/caregiver and patient (if ≥ 12 years) will view a multimedia interactive education program (MIPE – IP version) during their scheduled clinic visit; the institutional CRA will submit documentation for each patient randomized to the IP arm to certify completion of this portion of the intervention (see Day 29 Documentation Form). There are two IP versions of the MIPE program, depending on the age of the participant: < 12 years and ≥ 12 years. The content is identical to the MIPE EDU program, except the IP version also includes details about the customized instructions, cell phone reminders, and DST.

4.1.5.2.2 Reminder System (REM)

On Days 29, 57, 85, and 113 (scheduled clinic visits), the patient's healthcare provider prepares and prints a customized electronic schedule in the patient's preferred language from the ***ACCL1033 MEDACTIONPLAN ALL 6MP Schedule Template.*** *MEDACTIONPLAN* is a secure, HIPAA-compliant web-based application[37] (*MEDACTIONPLAN*; www.medactionplan.com) that allows healthcare providers to quickly create customized medication schedules for patients. A customized *MEDACTIONPLAN* portal was created specifically for the ACCL1033 study (https://www.medactionplan.com/ct/network.asp). The healthcare provider prepares a customized 6MP schedule during the patient's routine clinic visit using the *MEDACTIONPLAN ALL 6MP Schedule Template* to specify 6MP dose and frequency of administration.

Medication-specific instructions (e.g., "do not take with dairy products") and the purpose of each medication (e.g., "treats leukemia") are included within the schedule and can be modified as needed by the healthcare provider. A permanent electronic record of the medication schedule is saved in the web-based application; the schedule is then printed for the patient in English or instantaneously converted into Spanish as needed prior to printing. Next, the healthcare provider activates **automated customized SMS text messaging reminders to be delivered via cellular phone according to the patient's electronic 6MP schedule**. These customized reminders are delivered via cell phone to the parent/caregiver and patient at the scheduled time of each 6MP dose, in their preferred language (English or Spanish), and include the name of the drug (6MP), number of tablets prescribed (e.g., "take 1-1/2 tablets"), time due (e.g., "at 9 pm"), and name of the healthcare provider (e.g., "Dr. Martin"). **The entire process of creating/ printing a customized 6MP schedule and activating the automated web-based reminders within *MEDACTIONPLAN* requires less than 5 minutes of the healthcare provider's time.** Medication schedules can be changed and updated by the healthcare provider at any time. Web-based text reminders are updated concurrently with scheduling modifications. Standardized instructions regarding the REM portion of the intervention will be provided to the CRAs for dissemination to the healthcare providers caring for patients randomized to the IP arm prior to Day 29 (*Intervention Instructions for the Healthcare Provider*) and parents/caregivers on Day 29 (*Intervention Instructions for the Designated Parent/Caregiver*). On Day 29, the CRA will review the instructions with the parent/caregiver and patients (≥ 12 years).

4.1.5.2.3 Directly Supervised Therapy (DST)

On Day 29, the designated parent/ caregiver who enters into a mutually agreed upon partnership with the patient, is trained briefly in prompting and supervising the patient take his/ her medication on a daily basis. Once the designated parent/caregiver receives the text-message, they are instructed to prompt initiation of the dose (if necessary), and to supervise the ingestion of the prescribed 6MP (in all instances). If the patient is not present when the dose of 6MP is due (patients who travel between two households, away at college, staying with a relative, etc.), the designated parent/caregiver will call the patient (or other parent/caregiver for patients < 12 years) to prompt initiation of the dose. After the patient has taken the scheduled dose, the designated parent/caregiver and patient (if ≥ 12 years) respond to the text message with a standardized reply function on the cell phone, providing a real-time central record of adherence. This training for DST is a component of the MIPE program (IP version only) that is viewed on Day 29 by parents/caregivers and patients (if ≥ 12 years); additionally, standardized instructions will be provided to parents/caregivers regarding the DST portion of the intervention on Day 29 (*Intervention Instructions for the Designated Parent/Caregiver*). On Day 29, the CRA will review the instructions with the parent/caregiver and patients (≥ 12 years). Parents/caregivers and patients (if ≥ 12 years) who do not have a cell phone, or who have limited or no text-messaging capability on their current cell phone, will be issued a study cell phone with unlimited phone and text messaging capabilities for use for the 4-month duration of the intervention. For patients aged 12-17 years, parental permission for the child to use the cell phone will be obtained during the consent process; patients whose parents do not grant permission for cell phone use will be ineligible to participate (see Section 3.2.1., Inclusion Criteria); salient demographics will be recorded for these patients to ensure that this population does not differ in any systematic way from the patients who are allowed use of cell phones (Study Refusal form - see Section 4.1.1). CRAs will indicate on the OnStudy eRDE form if a cell phone needs to be assigned to the parent/caregiver and/or patient, and the coordinating center (Children's of Alabama/UAB), will mail the phone(s) to the CRA.

4.1.5.2.4 Alternative Intervention Training for the Designated Parent/Caregiver

The designated parent/caregiver should be strongly encouraged to be present at the Day 29 appointment. However, if they are unable to accompany the patient to the Day 29 appointment, the CRA will provide an Alternative Intervention Training Packet to be sent home with the patient. The Alternative

Intervention Training Packet will include the MIPE IP program (< 12 years or ≥ 12 years version), detailed instructions for the DST and REM portions of the intervention, and a letter with instructions for completing the intervention training within 24 hours of the patient's Day 29 clinic appointment. The CRA will call the designated parent/caregiver the following business day to review the instructions and confirm that the designated parent/caregiver has completed the Alternative Intervention Training. The CRA will document this on the Day 29 Documentation Form. If the Alternative Intervention Training has not been completed at the time that the CRA makes the follow-up call, the CRA will ask the parent/caregiver if they agree to complete the training on that day. If the parent/caregiver agrees, the CRA will call back the following day to confirm that the training has been completed and will document this on the Day 29 Documentation Form; if the training has not been completed at the time of the second follow-up call, the patient will be removed from the study and the CRA will complete the Off Study form. If the CRA is unable to reach the designated parent/caregiver on Day 29 and/or for the second follow-up call, the CRA will continue calling daily until contact is made.

4.1.5.2.5 Day 29 Documentation Form

On Day 29, the institutional CRA will complete the Day 29 Documentation Form, which includes documentation of (1) completion of MIPE viewing by the patient (if ≥ 12 years) and designated parent/caregiver (IP and EDU arms) and (2) provision of standardized instructions regarding the REM and DST portions of the intervention to the healthcare provider, patient (if ≥ 12 years) and parent/caregiver (IP arm only). If the designated parent/caregiver is not present at the Day 29 appointment, the CRA will document that an Alternative Intervention Training Packet (IP arm only) or Alternative MIPE EDU Viewing Packet (EDU only) was sent home with the patient and a follow-up phone call was made on the following business day to confirm completion of training. The CRA will submit the completed Day 29 Documentation Form within 1 week of the Day 29 clinic visit to Dr. Smita Bhatia at the study coordinating center (Children's of Alabama/UAB) via email, or fax (contact information below).

4.2 Additional Data

For each patient registered on ACCL1033, a Disease History and Therapeutic Summary, five Maintenance Report Worksheets, and a Day 141 Lab form, will be prepared by the institutional CRA, <u>reviewed by the institutional Primary Investigator (PI) or PI's designee,</u> and faxed, or emailed to the coordinating center (Children's of Alabama/UAB). These forms will summarize pertinent data from the medical records, such as: history, physical examination, and diagnostic data at diagnosis (including cytogenetics). In addition, CRAs will confirm any usual care practices pertaining to medication adherence with the patient's treating clinician (e.g. giving the patient a medication calendar, pill counts, parent signing form stating patient has taken medications, measurement of 6TGN levels), and document these on the Disease History and Therapeutic Summary form. Appendix II summarizes the data submission requirements for ACCL1033.

Within 4 weeks of study enrollment, please complete the Disease History and Therapeutic Summary Worksheet and mail, fax, or email the completed worksheet to the coordinating center. In addition, a Maintenance Report Worksheet should be completed **<u>for each 4 week maintenance period the patient completes</u> during the study period (5 total).** This includes the following details: 6MP doses with dates and reasons for any 6MP doses that were held during the designated study period; CBC reports and liver function tests during the designated study period, and history of all transfusions during the designated study period. Please email or fax **within 4 weeks** of completion of each timepoint to the coordinating center:

FAX or EMAIL worksheets to:
Dr. Smita Bhatia
Fax: (205) 212-3400
Email: AdherenceStudy@peds.uab.edu

At the completion of a patient's study participation (Day 141), cell phone(s) issued from Children's of Alabama/UAB are mailed back <u>in a padded envelope</u> (may be combined with the MEMS® *TrackCap*™) to:

Dr. Smita Bhatia
UAB Division of Peds Hem/Onc
1600 7th Ave S, Lowder 500
Birmingham, AL 35233-1711

The Study Schema is detailed in Table 5.

Table 5. Study Schema

	Prior to Day 1	Day 1	Days 1-28	Day 29	Day 57	Day 85	Day 113	Day 141
Assent/Consent, supply kit issued to patient	√							
6MP dispensed in MEMS bottle with standard caps (IP and EDU)	√							
CRA enrolls patient on study via COG eRDE system		√						
Randomization to IP vs. EDU		√						
TPMT phenotyping/ Demographic Questionnaire (IP and EDU)		√						
MEMS *TrackCap*™ given to patient (IP and EDU)		√						
Acculturation Questionnaire (Hispanic and Asian participants only)		√						
Depression Questionnaire (IP and EDU)		√						
Electronic monitoring of 6MP via MEMS (IP and EDU)			●	—	—	—	→	
Break-in period (obtain baseline assessment of adherence [IP, EDU])			●→					
Parental Distress (PIP) Questionnaire (IP and EDU)						√		
MEMS returned and mailed to Coordinating Center for data download (IP and EDU)								√
Red blood cell 6TGN assay (IP and EDU)				√	√	√	√	√
Adherence self-report questionnaire - comprehensive (IP & EDU)		√			√			√
Adherence self-report questionnaire – abbreviated (IP & EDU)				√		√	√	
Multimedia Interactive Patient Education Program (IP and EDU)				√				
Standardized training for all intervention components (IP)				√				
Cell phone(s) issued to patient and parent/caregiver if needed (IP)				√				
Customized 6MP Instructions + Text Reminders (IP)				●	—	—	→	
DST/6MP ingestion prompted by Text Reminders (IP)				●	—	—	→	
Intervention Rating Questionnaire (IP) or MIPE Rating Questionnaire (EDU)								√

4.3 Specimen Collection and Submission

Blood samples will be obtained at the time of regularly scheduled blood draws on Day 1 of the study (TPMT phenotyping), and then on Days 29, 57, 85, 113, and 141 (6TGN levels) as described below and in Appendix III. Self-explanatory blood draw kits will be sent by the coordinating center (Children's of Alabama/UAB, 205-638-2129) to each participating institution. Each kit will contain instructions, blood sampling tubes and mailing supplies.

Day 1: Whole blood (5 ml) in a green top (sodium heparin) tube (TPMT phenotyping)

Days 29, 57, 85, 113, 141: Whole blood (5 ml) in a green top (sodium heparin) tube (6TGN levels)

Ship samples on **refrigerant pack** included in blood draw kits (a.k.a. wet ice) by overnight **PRIORITY (morning)** delivery within 24 hours (specify Saturday delivery if shipping on Friday) to:

Dr. Mary Relling
Clinical Pharmacokinetics Laboratory
St. Jude Children's Research Hospital
262 Danny Thomas Place, MS 313, CCC Rm-I5411
Memphis, Tennessee 38105
Phone: (901) 595-2242

When shipping study specimens to the St. Jude Clinical Pharmacokinetics Laboratory for ACCL1033, please use the **COG FedEx account number** (See the "Biology" section of the COG website for current account number and an explanation of FedEx accounts). **NOTE: Please notify the coordinating center when specimens are sent by faxing a copy of the completed lab slip to (205) 212-3400 or emailing a copy to AdherenceStudy@peds.uab.edu.**

Staff is available Monday-Saturday to accept samples. There is no need to call the lab regarding weekend/holiday deliveries. If there are specific holiday shipping requirements, a Groupwide memo will be posted on the COG website.

Please direct inquiries regarding specimen collection/shipment to the coordinating center (Children's of Alabama/UAB, 205-638-2129; AdherenceStudy@peds.uab.edu).

4.4 Laboratory Analysis

4.4.1 RBC TPMT Assay

Red cell TPMT activity will be measured by the method of Weinshilboum et al. The assay (described elsewhere)[45] is based on the conversion of 6MP to radioactively labeled 6-methylmercaptopurine with C-methyl-S-adenosyl-L-methionine as the methyl donor. One unit of enzyme activity represents the formation of 1 nmol of S-methylmercaptopurine/hr of incubation.

4.4.2 RBC Metabolites Assay

Red cell 6TGN levels will be measured by a modification of a previously described assay.[46] Nucleotides are dephosphorylated to nucleosides by treatment with alkaline phosphatase, and the thiopurine bases and ribosides are quantified by HPLC, using UV detection and a diode array detector to confirm the identity of all metabolites.

4.5 Follow-Up Data Collection

Study participants will be followed closely at their respective treating institutions for the following events: i) death, ii) relapse and iii) second malignancy. For patients that relapse or develop a second malignancy, institutions will need to submit documentation of the event (clinic note, pathology report, etc.). The coordinating center will contact participating institutions to obtain an update on the vital and

disease status of the study participants. A follow-up form will be requested every 6 months for 5 years from the time of completion of the study, and then annually until 10 years from diagnosis.

5.0 STATISTICAL CONSIDERATIONS

5.1 Statistical Design

This is a randomized clinical trial. This trial determines the impact of interventions proposed in IP vs. that proposed in EDU on adherence to oral 6MP in children with ALL. At the time of enrollment, participants will be automatically randomized via the eRDE system. Eligible participants will be randomized to a study assignment (IP or EDU) using a blocked stratified randomization with ethnic background (4 strata) and age at the time of randomization (2 strata) as stratification factors and a block size of 6 to balance the number of participants in each arm.

Table 6. ACCL1033 Patient Accrual

	**<12 years (accounting for attrition)**	**≥12 years (accounting for attrition)**	**Total number needed for trial**	**Total number to be enrolled to account for attrition**
Non-Hispanic white	132	92	170	224
Hispanic	132	92	170	224
African American	48	32	58	80
Asian/Other	48	32	58	80
Total	360	248	456	608
See Section 3.1.1 for the race/ethnicity definitions				

5.2 Accrual (and Expected Duration of Accrual)

We plan to enroll a maximum of 608 subjects, 304 each in IP and EDU arms, and within each arm the numbers to be enrolled are shown by race/ ethnicity and age in Table 6, such that, after accounting for attrition due to study withdrawal there will be 228 evaluable patients in the IP arm and 228 patients in the EDU arm, for a total of 456 evaluable patients. The expected duration of accrual is 48 months. Based on the 136 institutions that have participated in AALL03N1, these institutions diagnose 1,784 children with ALL each year (non-Hispanic whites: n=1,123; Hispanics: n=381, African-Americans: n=118, Asians: n=77; Others: n=45). Thus, over the 48-month accrual period, these sites will see approximately 4,500 newly diagnosed non-Hispanic whites, 1,525 Hispanics, 475 African-Americans, 300 Asians, and 180 Others with ALL; providing an ample sized pool for recruitment.

5.3 Analytic Plan

Eligible participants will be randomized to **EDU** or **IP** using blocked stratified randomization (8 strata) with racial/ethnic background (NHW, Hispanics, AA, Asians /other) and age at study (<12y , ≥12 years) as stratification factors and a block size of 6 to balance participant number/ type in each arm.

Adherence measured by MEMS will be treated as a binomial variable, and that measured by TGN levels as a continuous variable. MEMS-based adherence rate will be defined as the ratio of days with MEMS cap openings (N) to days 6MP was prescribed (D) for each patient, reported as a percent (N/D*100). Days when 6MP was held by prescriber will be removed from the denominator. Adherence measurements will be calculated for 5 time points (Days 29, 57, 85, 113, 141), each reflecting adherence over the preceding 28 days. Intervention begins on Day 29. Since participants are randomized, baseline adherence rate (Day 29) is not expected to differ between EDU and IP. Nevertheless, we will evaluate the success of randomization by comparing baseline group differences in adherence, demographic, clinical and psychosocial variables. We will conduct propensity score analysis to adjust for imbalance, to obtain unbiased estimates of intervention effects. We will use multiple imputations for missing data before conducting propensity analyses.

<u>Primary Aim 1:</u> Determine impact of intervention package (**IP)** vs. education **(EDU)** on adherence to oral 6MP in children with ALL. Adherence measured by: i) **MEMS** (primary measure); and ii) red cell **TGN levels**

Hypothesis 1.1: **IP** will result in a higher proportion of patients with adherence rates ≥95% compared with **EDU**. **Hypothesis 1.2: IP** will result in a higher proportion of ≥12 years old patients with adherence rates ≥95% compared with **EDU**; **IP** will result in a higher proportion of <12y-old patients with adherence rates ≥95% compared with **EDU**, allowing evaluation of the developmentally-tailored intervention

The primary outcome of this trial is the proportion of patients with adherence rate ≥95% to 6MP over the duration of the intervention. Using an intention-to-treat analysis, the effectiveness of IP will be determined by comparing the proportion of adherent patients in the IP and EDU groups using logistic regression. This test will be conducted for all ages combined (**Hypothesis 1.1**) and separately for <12y and ≥12 years olds (**Hypothesis 1.2**). We will also examine longitudinal changes in red cell TGN levels between the two arms using GEE for longitudinal normally distributed data to corroborate results from the MEMS data analysis. The TGN analysis will be limited to patients with normal TPMT activity and TGN will be adjusted for 6MP dose-intensity.

Exploratory Aim 1: Examine the modifying effect of sociodemographic and psychosocial variables, and the mediating effect of health beliefs/ knowledge on change in adherence with intervention

Hypothesis E1: Certain subgroups will derive more benefit from IP (*<u>modifiers</u>*: **Table 1**). Changes in adherence with MIPE will be *<u>mediated</u>* by change in health knowledge/beliefs **(Table 1).** Impact of MIPE as a mediator will be stronger in IP because of additional behavioral reinforcement by customized printed schedules, text message reminders and DST.

Interactions of group by modifying and mediating covariates will be included in the logistic regression model and their significance tested. To better understand the effects of mediators, individuals' adherence rates (as binomial variable) will be modeled longitudinally using GEE methods (with compound symmetry as working correlation matrix) by including the interactions of treatment group by time and treatment group by time by mediator variable. This will enable examination of whether changes in proportion of adherent patients in each group resulted from changes in individuals' adherence rates as a consequence of variation in mediator variables. As power for examining interaction is expected to be low, these analyses will be exploratory.

<u>Exploratory Aim 2:</u> Establish the infrastructure to determine the impact of **IP** vs. **EDU** on risk of relapse in children with ALL

Hypothesis E2: IP will result in lower risk of relapse compared with **EDU** – upon mature follow-up of the two groups

An intention-to-treat analysis will be used to compare the effectiveness of EDU and IP interventions in decreasing the risk of relapse. Cox proportional hazards regression models will be used to examine the impact of intervention on relapse. Covariates in the analysis will include clinical and sociodemographic predictors, and the intervention arm (IP vs. EDU).

5.4 Power and Sample Size

Sample size and power determination was based on binomial distribution, classifying each patient as "adherent" (adherence rate ≥95%) or "non-adherent" (adherence rate <95%). Sample size calculation was performed using NCSS PASS. ***Aim 1:*** The effectiveness of IP over EDU will be determined by comparing P_{IP} (proportion of patients with adherence rate ≥95% in IP) to P_{EDU} (the corresponding rate in EDU), by testing Ho: $P_{IP} = P_{EDU}$ in all ages combined as well as in <12y and ≥12 years olds. To account for 3 independent tests, a 2-sided Bonferroni-adjusted Type I error=0.017 (for an overall Type I error=0.05) will be used for each test. Since the value for P_{EDU} is unknown, we assumed its value to be at least that observed under usual care in AALL03N1 and increased it up by +5% and +10%. Thus, for <12y, we let P_{EDU} =0.60 (observed in AALL03N1), 0.65, 0.70. For ≥12 years, we let P_{EDU} =0.42, 0.47, 0.52. For both age groups combined, we let P_{EDU} =0.56, 0.61, 0.66. Of note, these values included patients whose estimated adherence rates were 100% in AALL03N1. When both age groups are combined (**Hypothesis 1.1**), the sample size of 456 (228 per arm) has 80% power to detect a difference between P_{EDU} =0.56 to 0.66 and P_{IP} =0.70 to 0.79 (i.e., 70%-79% of IP patients have adherence rates ≥95% vs. 56%-66% EDU patients), or an Odds Ratio (OR) =1.9-2.0. Under **Hypothesis 1.2**, for <12y, 133 patients/arm will provide 80% power to detect a difference between P_{IP} =0.78-0.86 and P_{EDU} =0.60-0.70, or an OR =2.4-2.7. For ≥12 years, 95 patients/arm will provide 80% power to detect a difference between P_{IP} =0.65-0.74 and P_{EDU} =0.42-0.52, or an OR =1.9-2.0. Thus, this study is powered to detect a minimum improvement of 17 to 23 percentage points in the proportion of adherent patients with the intervention. This effect size is commensurate with published intervention studies that demonstrate an effect size range of 17.8%[47] to 46%.[34]

Exploratory Aim 1: Adequate power for detecting a modifier/mediator of treatment effect requires a larger sample size; analysis will therefore be considered exploratory. *Exploratory Aim 2:* At 2-sided Type I error of 0.05, assuming a 10% relapse rate over 5y of follow-up, there is 80% power to detect a hazard ratio of 2.7.

Feasibility of achieving target enrollment: In the observational trial (AALL03N1) the attrition rate was 16%. We have inflated the target enrollment from 456 (evaluable patients needed for Aim 1) to 608 (accounting for a higher 25% attrition anticipated in an intervention trial). **Table 6** demonstrates the number of patients needed by race/ethnicity within each age group (based on AALL03N1 age distribution).

5.5 Gender and Minority Accrual Estimates

The gender and minority distribution of the study population is expected to be:

Accrual Targets			
**Ethnic Category**	**Sex/Gender**		
	**Females**	**Males**	**Total**
Hispanic or Latino	90	134	224
Not Hispanic or Latino	154	230	384
**Ethnic Category: Total of all subjects**	244	364	608*
**Racial Category**			
Asian (including Native Hawaiian and other Pacific Islander)	16	24	40
Black or African American	32	48	80
Other (including American Indian and Alaskan Native)	16	24	40
White	180	268	448
**Racial Category: Total of all subjects**	244	364	608*

* These totals must agree
This distribution was derived from AALL03N1.

6.0 DATA SAFETY AND MONITORING

As this is a low risk behavioral intervention not falling under the purview of the Children's Oncology Group Data Safety Monitoring Committee (DSMC), the DSMC of record will be located at the study coordinating center (Children's of Alabama/UAB). The Study PI (Smita Bhatia) will be responsible for monitoring protocol conduct and reporting any deviations, unanticipated problems, adverse events and/or serious adverse events related to protocol intervention and/or research procedures performed on <u>this study</u> to the Children's of Alabama/UAB DSMC. If a patient experiences a serious adverse event, or unanticipated problem <u>related to their participation in ACCL1033</u>, you must notify the coordinating center (AdherenceStudy@peds.uab.edu, (205) 638-2129) within 5 calendar days. Adverse events related to ACCL1033 participation that do not meet the criteria of serious OR are not unanticipated problems should be reported to the study coordinating center as soon as possible and no later than 4 weeks following the event. <u>Please note that adverse events, serious adverse events and unanticipated problems related to the patient's ALL treatment protocol do not have to be reported to the ACCL1033 coordinating center.</u>

APPENDIX I: YOUTH AND PARENT/CAREGIVER INFORMATION SHEETS

INFORMATION SHEET REGARDING RESEARCH STUDY
(for children who are 7 to 11 years of age)

A Study of a Multi-Part Program to Help People Take Their Medicine at Home

1. We have been talking with you about your maintenance treatment for acute lymphoblastic leukemia (ALL). One problem with maintenance treatment is remembering to take medicines at home like you're supposed to.

2. We are asking you to take part in a research study because you are in maintenance treatment for ALL and you are taking a medicine called "6MP" by mouth. A research study is when doctors work together to try out new ways to help people who are sick. In this study, we are trying to learn more about how to help people take their 6MP at home as they should. We will do this by trying a new program to help people be better at taking their 6MP like they're supposed to.

3. Children who are part of this study will continue with their maintenance treatment as their doctor ordered. They will receive a special pill bottle to keep their 6MP medicine in. The bottle also has a special cap that records when the bottle is opened. You will use this bottle during the study. At the end of the study you will give the special cap back to the study doctors. Your parent will also be asked to answer some questions about your family, how you are feeling, and about how you are doing with taking your 6MP. Your parent will also watch a video on the Internet or on a DVD during one of your clinic visits. In the video, people talk about taking 6MP. Some children and their parents will take part in a program designed to help them remember to take their 6MP like they should. Parents of these children will also receive text messages on their cell phones reminding them to give 6MP each time it is due, and the parent will make sure that the child takes each dose of 6MP during the study. If parents do not have cell phones or text messaging, they will be given cell phones with text messaging to use during the study. Other children will have their clinic visits as usual. You have the same chance of being asked to take part in the cell phone text messaging reminder program or not. You or your doctor will not decide this.

4. As part of this study, you will have some blood taken every month for a total of 6 times. We will collect the blood during your normal clinic visits. We will collect the blood at the same time you would normally have blood drawn, so there would be no extra needle sticks.

5. Sometimes good things can happen to people when they are in a research study. These good things are called "benefits." We hope that a benefit to you of being part of this study is helping us learn more about how to help people take their medicine like they should. But we don't know for sure if there is any benefit of being part of this study.

6. Sometimes bad things can happen to people when they are in a research study. These bad things are called "risks." One risk to you from this study is possible pain, bruising, or infection at the site of the needle stick for blood draws, but since you will already be having the blood test anyway, this risk is the same if you are in study or not. Another risk is worry or concern about watching the Internet/DVD program or worry or concern answering study questions. Other things may happen to you that we don't yet know about.

7. Your family can choose to be part of this study or not. Your family can also decide to stop being in this study at any time once you start. Make sure to ask your doctors any questions that you have.

INFORMATION SHEET REGARDING RESEARCH STUDY
(for teens from 12 through 17 years of age)

A Study of a Multi-Part Program to Help People to Take Their Medication at Home

1. We have been talking with you about your maintenance treatment for acute lymphoblastic leukemia (ALL). One problem with maintenance treatment is remembering to take medications at home like you're supposed to.

2. We are asking you to take part in a research study because you are in maintenance treatment for ALL and you are taking a medication called "6MP" by mouth. A research study is when doctors work together to try out new ways to help people who are sick. In this study, we are trying to learn more about how to help people take their 6MP at home as they should. We will do this by trying a new program to help people be better at taking their 6MP like they're supposed to.

3. Children and teens who are part of this study will continue with their maintenance treatment as their doctor ordered. They will receive a special pill bottle to keep their 6MP medication in, which has a special cap that records when the bottle is opened. You will use this bottle during the study and at the end, you will return the special cap to the study doctors. You and your parent/caregiver will also be asked to answer some questions about your family, how you are feeling, and about how you are doing with taking your 6MP. You and your parent will also be asked to watch an interactive video program on the Internet or on a DVD during one of their clinic visits. In the interactive video, people talk about taking 6MP. Some teens and their parents will take part in a program designed to help them remember to take their 6MP like they should. These teens and their parents will also receive text messages on their cell phones reminding them to take 6MP each time it is due, and the parent will make sure that the teen takes each dose of 6MP during the study. If teens and parents do not have cell phones or text messaging, they will be given cell phones with text messaging to use during the study. Other teens will have their clinic visits as usual. If you agree to be in this study, there is a 1 in 2 (or 50-50) chance that you will be asked to take part in the cell phone reminder program. That means there is also a 1 in 2 (50-50) chance that you will not be asked to take part in the cell phone text messaging reminder program.

4. As part of this study, you will have some blood taken once a month for a total of 6 times. We will collect the blood during your normally scheduled clinic visits. We will collect the blood at the same time you are having blood drawn for your maintenance treatment, so there would be no extra needle sticks.

5. Sometimes good things can happen to people when they are in a research study. These good things are called "benefits." We hope that a benefit to you of being part of this study is helping us learn more about how to help people take their medications as they should. But we don't know for sure if there is any benefit of being part of this study.

6. Sometimes bad things can happen to people when they are in a research study. These bad things are called "risks." One risk to you from this study is possible pain, bruising, or infection at the site of the needle stick for blood draws, but since you will already be having the blood test anyway, this risk is the same if you are in study or not. Another risk is discomfort watching the Internet/DVD program or discomfort answering study questions. Other things may happen to you that we don't yet know about.

7. Your family can choose to be part of this study or not. Your family can also decide to stop being in this study at any time once you start. Make sure to ask your doctors any questions that you have.

INFORMATION SHEET REGARDING RESEARCH STUDY ACCL1033
(for parents and caregivers of children enrolled on ACCL1033)

A Study of a Multi-Part Program to Help People Take Their Medicine at Home

1. We are asking you to take part in a research study because your child is in maintenance treatment for ALL and is taking a medicine called “6MP” by mouth. One problem with maintenance treatment is children and teens remembering to take medicines at home like they are supposed to. In this study, we are trying to learn more about how to help children and teens take their 6MP at home as they should. We will do this by trying a new program to help children and teens improve their 6MP-taking practices. This program requires the participation of both the child and the child’s parent or caregiver. We are providing this information to you to help you decide if you want to participate in this research study with your child or teen.

2. Children and teens who are part of this study will continue with their maintenance treatment as their doctor ordered. They will receive a special pill bottle (called MEMS) to keep their 6MP medicine in. The bottle also has a special cap (called MEMS TrackCap™ CR) that records when the bottle is opened. Your child will use this bottle during the study. At the end of the study you/your child will give the special cap back to the study doctors.

3. You and your child (if $\geq$ 12 years old) will be asked to complete 9 to 11questionnaires over the course of the study. These questionnaires ask about your family (sociodemographic information, such as the number of people in your household, your educational level, and income), how you and your child are feeling, and how your child is doing with taking their 6MP. If you/your child is Hispanic or Asian, you will also be asked to complete a questionnaire about language preferences.

4. You and your child (if $\geq$ 12 years old) will view an educational video (available in English and Spanish) on the Internet or on a DVD during one of the child’s clinic visits. In the video, patients and their parents talk about their experiences with 6MP.

5. **Intervention Program (Arm IP):** During this research study, some children and their parents will take part in an intervention program designed to help patients remember to take their 6MP as they should. Children and teens taking part in the intervention program will receive text messages sent to their cell phone (if the child is $\geq$12 years old) and the cell phone of their parent (or designated caregiver) reminding them to take 6MP each time it is due. The parent will make sure that the child takes each dose of 6MP during the study. If the child (if $\geq$12 years old) and/or their parent does not have a cell phone, or if their cell phones have limited or no text messaging capabilities, they will be given cell phones with text messaging to use during the study. Children and teens taking part in the intervention program will also be given a 6MP medication schedule that has been created especially for them. Parents will receive a short training session about how to prompt and supervise their child to take their 6MP dose after receiving the text message reminders. After each 6MP dose, both parent and child (if $\geq$ 12 years old) will respond to the text message indicating that the dose was taken. At the end of the study, parents and children (if $\geq$12 years old) participating in this intervention program will be asked to complete an additional questionnaire about whether the program was helpful.

6. **Education Only (Arm EDU)**: During this research study, some children will have their clinic visits as usual. Your child has the same chance of being asked to take part in the cell phone text messaging reminder program or not. You, your child, and your child's doctor will not decide this.

7. As part of this study, your child will have some blood taken at 6 times. We will collect the blood during your child's normal clinic visits. We will collect the blood at the same time your child would normally have blood drawn, so there would be no extra needle sticks. At the second clinic visit since entering the study, a blood test (TPMT phenotyping) will be done to test for how your child's body handles 6MP, and blood tests at clinic visits 3 to 7 will measure the levels of 6MP in your child's blood. For each of these blood tests, we will collect 5 mL (about 1 teaspoon) of blood.

8. Sometimes good things can happen to people when they are in a research study. These good things are called "benefits." We hope that a benefit to you and your child as being part of this study is helping us to learn more about how to help people take their medicine like they should. But we don't know for sure if there is any benefit of your or your child's being part of this study.

9. Sometimes bad things can happen to people when they are in a research study. These bad things are called "risks." One risk to your child from this study is possible pain, bruising, or infection at the site of the needle stick for blood draws, but since your child will already be having blood tests anyway, the risk is the same if your child is in the study or not. Another risk is your or your child's worry or concern about watching the Internet/DVD program or discomfort answering study questions. Other things may happen to you or your child that we don't yet know about.

10. You, your child, and your family can choose to be part of this study or not. You, your child, and your family can also decide to stop being in this study at any time once you start. Make sure to ask your child's doctors any questions that you have.

APPENDIX II: Data submission requirements for ACCL1033

<table>
<tr><th>Report</th><th>When due</th><th>Submit to:</th></tr>
<tr><td>Eligibility and On Study eRDE Forms</td><td>At time of study enrollment; OnStudy due next business day following enrollment or as soon as contact is made with the family (the OnStudy form will be overdue after 7 days)</td><td>On-line via eRDE system</td></tr>
<tr><td>Study Refusal Form</td><td>As soon as possible from the dateparent/patient refuses consent ; may also be submitted with Study Participation Worksheets</td><td rowspan="9">FAX all reports to:
Dr. Smita Bhatia
(205) 212-3400

<u>OR</u> scan and email to:
AdherenceStudy@peds.uab.edu</td></tr>
<tr><td>Study Participation Worksheet</td><td>As soon as possible and no later than 4 weeks of request from coordinating center (requested every 3 months)</td></tr>
<tr><td>Demographic Questionnaire (completed by parent/ caregiver or patient (if ≥ 18 years)on Day 1)</td><td rowspan="2">As soon as possible and no later than 1 week of study Day 57</td></tr>
<tr><td>Acculturation Questionnaire (completed by Hispanic and Asian patients (if ≥ 12 years) and parent/caregivers only on Day 1)</td></tr>
<tr><td>BDI-II Depression Questionnaire (Designated Parent/Caregiver and Patients 18 years and older)</td><td>As soon as possible and no later than 1 week of study Day 57</td></tr>
<tr><td>CDI 2 Depression Questionnaire (self-report for patients 12-17 years; parent report for patients younger than 12 years)</td><td>As soon as possible and no later than 1 week of study Day 57</td></tr>
<tr><td>Disease History and Therapeutic Summary Worksheet</td><td>As soon as possible and no later than 1 week of study Day 57</td></tr>
<tr><td>Maintenance Report Worksheets</td><td>As soon as possible and no later than 4 weeks of each timepoint, starting with Day 29 study timepoint</td></tr>
<tr><td>Day 29 Documentation Form (completed by CRA on Day 29)</td><td>Within 1 week of the Day 29 study visit</td></tr>
</table>

Adherence Questionnaires (completed by the parent/caregiver and patient (if ≥ 12 years) on Days 1, 57, and 141 [comprehensive]; and Days 29, 85, and 113 [abbreviated])	As soon as possible and no later than 2 weeks from the time **the questionnaire is completed and returned**	
Intervention Rating Questionnaire (IP) or MIPE Rating Questionnaire (EDU) ; completed by the parent/caregiver and patient (if ≥ 12 years) on Day 141)		
Parental Distress (PIP) Questionnaire (Designated Parent/Caregiver only)	As soon as possible and no later than 1 week of study Day 113	
Day 141 Lab form	As soon as possible and no later than 4 weeks of the Day 141 study visit	
Off Study form	As soon as possible and no later than 2 weeks from the date patient goes off study	
Follow-up Forms	Requested by the coordinating center every 6 months for 5 years from the time of completion of the study, and then annually until 10 years from diagnosis; **Due as soon as possible and no later than 2 months of request from coordinating center.**	
**Report**	**When due**	**Mail to:**
MEMS® *TrackCap*™	Please ask patient to return on Day 141 clinic visit. Mail in a padded envelope to coordinating center as soon after Day 141 as possible (phone and cap may be mailed together). Due within 3 months of Day 141 visit, **but should be mailed as soon as received from patient and/or parent**.	Dr. Smita Bhatia<br>UAB Division of Peds Hem/Onc<br>1600 7th Ave S, Lowder 500<br>Birmingham, AL 35233-1711<br>(205) 638-2129
Cell phone(s)*<br>*IP only; if patient and/or parent/caregiver assigned a study cell phone		

* For the purposes of data submission, the due dates are based upon the ACCL1033 study timepoints, which do not necessarily correspond to actual calendar days. For example, it is possible to have a Study Day 29 that is 35 days from the Day 1 appointment (if patient rescheduled or delayed their appointment one week). The coordinating center will also report study deviations from all COG institutions.

APPENDIX III: Specimen Requirements for ACCL1033

Study Day	Specimen Specifics	Studies to be Performed:	Shipping:
1	5 ml blood in green top tube (sodium heparin)	TPMT phenotyping	Ship samples on **refrigerant pack** included in blood draw kits (a.k.a. wet ice) by overnight **PRIORITY (morning)** delivery within 24 hours (specify Saturday delivery if shipping on Friday) to:<br><br>Dr. Mary Relling<br>Clinical Pharmacokinetics Laboratory<br>St. Jude Children's Research Hospital<br>262 Danny Thomas Place, MS 313,<br>CCC Rm-I5411<br>Memphis, Tennessee 38105<br>Phone: 901 595-2242<br><br>Staff is available Monday-Saturday to accept samples. There is no need to call the lab regarding weekend/holiday deliveries. If there are specific holiday shipping requirements, a Groupwide memo will be posted on the COG website.<br>**Please direct inquiries regarding specimen collection/shipment to the coordinating center (Children's of Alabama/UAB 205-638-2129).**<br><br>When shipping study specimens to the St. Jude Clinical Pharmacokinetics Laboratory for ACCL1033, please use the **COG FedEx account number.** (See the "Biology" section of the COG website for current account number and an explanation of FedEx accounts).<br><br>**NOTE: Please notify the coordinating center when specimens are sent by faxing a copy of the completed lab slip to (205) 212-3400 or emailing to AdherenceStudy@peds.uab.edu**
29	5 ml blood in green top tube (sodium heparin)	6TGN	
57	5 ml blood in green top tube (sodium heparin)	6TGN	
85	5 ml blood in green top tube (sodium heparin)	6TGN	
113	5 ml blood in green top tube (sodium heparin)	6TGN	
141	5 ml blood in green top tube (sodium heparin)	6TGN	

* If the Day 1 TPMT sample is missed or within 90 days of a RBC transfusion, it may be obtained at anytime during the study. If the sample is not obtained for any other timepoint, the sample may be collected within one week of the corresponding study timepoint.

REFERENCES

1. Bhatia S, Sather HN, Heerema NA, et al: Racial and ethnic differences in survival of children with acute lymphoblastic leukemia. Blood 100:1957-64, 2002
2. Koren G, Ferrazini G, Sulh H, et al: Systemic exposure to mercaptopurine as a prognostic factor in acute lymphocytic leukemia in children. N Engl J Med 323:17-21, 1990
3. Relling MV, Hancock ML, Boyett JM, et al: Prognostic importance of 6-mercaptopurine dose intensity in acute lymphoblastic leukemia. Blood 93:2817-2823, 1999
4. Lennard L, Lilleyman JS: Variable mercaptopurine metabolism and treatment outcome in childhood lymphoblastic leukemia. J Clin Oncol 7:1816-23, 1989
5. Lennard L, Welch J, Lilleyman JS: Intracellular metabolites of mercaptopurine in children with lymphoblastic leukaemia: a possible indicator of non-compliance? Br J Cancer 72:1004-6, 1995
6. Davies HA, Lennard L, Lilleyman JS: Variable mercaptopurine metabolism in children with leukaemia: a problem of non-compliance? BMJ 306:1239-1240, 1993
7. Lau RC, Matsui D, Greenberg M, et al: Electronic measurement of compliance with mercaptopurine in pediatric patients with acute lymphoblastic leukemia. Med Pediatr Oncol 30:85-90, 1998
8. Bhatia S, Landier W, Hageman L, et al: Nonadherence to Oral 6-Mercaptopurine (6MP) in a Multi-ethnic Cohort of Children with Acute Lymphoblastic Leukemia (ALL) and its Impact on Relapse – a Children's Oncology Group (COG) Study (AALL03N1). Blood (ASH Annual Meeting Abstracts) 120:Abstract 882, 2012
9. Relling MV, Hancock ML, Rivera GK, et al: Mercaptopurine therapy intolerance and heterozygosity at the thiopurine S-methyltransferase gene locus. J Natl Cancer Inst 91:2001-8, 1999
10. Bhatia S, Villaluna D, Landier W, et al: Prevalence and predictors of non-adherence to 6-mercaptopurine in children with acute lymphoblastic leukemia - a Children's Oncology Group study. Blood 112:897, 2008
11. Bhatia S, Landier W, Shangguan M, et al: Non-Adherence to Oral 6-Mercaptopurine (6MP) Explains Ethnic Differences In Disease-Free Survival In Children with Acute Lymphoblastic Leukemia (ALL) - a Children's Oncology Group (COG) Study (AALL03N1). Blood 116:7, 2010
12. MacDougall LG, Wilson TD, Cohn R, et al: Compliance with chemotherapy in childhood leukaemia in Africa. S Afr Med J 75:481-484, 1989
13. Wright EC: Non-compliance--or how many aunts has Matilda? Lancet 342:909-913, 1993
14. Cramer JA, Mattson RH, Prevey ML, et al: How often is medication taken as prescribed? A novel assessment technique. JAMA 261:3273-3277, 1989
15. Mallion JM, Dutrey-Dupagne C, Vaur L, et al: Benefits of electronic pillboxes in evaluating treatment compliance of patients with mild to moderate hypertension. J Hypertens 14:137-144, 1996
16. Olivieri NF, Matsui D, Hermann C, et al: Compliance assessed by the Medication Event Monitoring System. Arch Dis Child 66:1399-1402, 1991
17. Urquhrt J: The electronic medication event monitor: lessons for pharmacotherapy. Clin Pharmacokinet 32:345-56, 1997
18. Macdougall LG, McElligott SE, Ross E, et al: Pattern of 6MP urinary excretion in children with ALL: urinary assays as a measure of drug compliance Ther Drug Monit 14:371-5, 1992
19. Bhatia S, Landier W, Shangguan M, et al: Nonadherence to oral mercaptopurine and risk of relapse in Hispanic and non-Hispanic white children with acute lymphoblastic leukemia: a report from the children's oncology group. J Clin Oncol 30:2094-101, 2012
20. Osterberg L, Blaschke T: Adherence to medication. N Engl J Med 353:487-97, 2005

21. Landier W, Hughes C, Calvillo E, et al: Understanding the barriers and facilitators to adherence to oral chemotherapy in a multiethnic cohort of youth with ALL. AACR Science of Health Disparities in Racial/Ethnic Minorities and the Medically Underserved Proceedings, 2009
22. Marin G, Perez-Stable EJ, Marin BV: Cigarette smoking among San Francisco Hispanics: the role of acculturation and gender. Am J Public Health 79:196-8, 1989
23. Latham CL, Calvillo E: A health protection model for Hispanic adults with Type 2 diabetes. J Clin Nurs 16:186-96, 2007
24. Suinn RM, Ahuna C, Khoo G: The Suinn-Lew Asian Self-Identify Acculturation Scale: concurrent and factorial validation. Educ Psychol Meas 52:1041-1046, 1992
25. DiMatteo MR, Lepper HS, Croghan TW: Depression is a risk factor for noncompliance with medical treatment: meta-analysis of the effects of anxiety and depression on patient adherence. Arch Intern Med 160:2101-7, 2000
26. Streisand R, Braniecki S, Tercyak KP, et al: Childhood illness-related parenting stress: the pediatric inventory for parents. J Pediatr Psychol 26:155-62, 2001
27. Lilleyman JS, Lennard L: Mercaptopurine metabolism and risk of relapse in childhood lymphoblastic leukaemia. Lancet 343:1188-1190, 1994
28. McDonald HP, Garg AX, Haynes RB: Interventions to enhance patient adherence to medication prescriptions: scientific review. JAMA 288:2868-79, 2002
29. Haynes RB, Ackloo E, Sahota N, et al: Interventions for enhancing medication adherence. Cochrane Database Syst Rev:CD000011, 2008
30. Williams PL, Storm D, Montepiedra G, et al: Predictors of adherence to antiretroviral medications in children and adolescents with HIV infection. Pediatrics 118:e1745-57, 2006
31. Weis SE, Slocum PC, Blais FX, et al: The effect of directly observed therapy on the rates of drug resistance relapse in tuberculosis. N Engl J Med 330:1179-84, 1994
32. Barclay E: Text messages could hasten tuberculosis drug compliance. Lancet 373:15-16, 2009
33. Revere D, Dunbar PJ: Review of computer-generated outpatient health behavior interventions: clinical encounters "in absentia". J Am Med Inform Assoc 8:62-79, 2001
34. Frick PA, Lavreys L, Mandaliya K, et al: Impact of an alarm device on medication compliance in women in Mombasa, Kenya. Int J STD AIDS 12:329-33, 2001
35. Erickson SR, Ascione FJ, Kirking DM, et al: Use of a paging system to improve medication self-management in patients with asthma. J Am Pharm Assoc (Wash) 38:767-9, 1998
36. Dunbar PJ, Madigan D, Grohskopf LA, et al: A two-way messaging system to enhance antiretroviral adherence. J Am Med Informat Assoc 10:11-15, 2003
37. Chuang W, Pineiro MJ, Ginsburg A, et al: Medactionplan.com as a tool for patient education and medication management in the blood and marrow transplant setting. BBMT 15:Suppl 1:34, 2009
38. Makoul G, Cameron KA, Baker DW, et al: A multimedia patient education program on colorectal cancer screening increases knowledge and willingness to consider screening among Hispanic/Latino patients. Patient Educ Couns 76:220-6, 2009
39. Krishna S, Francisco BD, Balas EA, et al: Internet-enabled interactive multimedia asthma education program: a randomized trial. Pediatrics 111:503-10, 2003
40. Marin G, Sabogal F, Marin BO, et al: Development of a short acculturation scale for Hispanics. Hispanic J Behav Sci 9:183-205, 1987
41. Beck AT, Steer RA, Ball R, et al: Comparison of Beck Depression Inventories -IA and -II in psychiatric outpatients. J Pers Assess 67:588-97, 1996
42. Beck AT, Ward CH, Mendelson M, et al: An inventory for measuring depression. Arch Gen Psychiatry 4:561-71, 1961
43. Saylor CF, Finch AJ, Jr., Spirito A, et al: The children's depression inventory: a systematic evaluation of psychometric properties. J Consult Clin Psychol 52:955-67, 1984

44. Del Rincon C, Remor E, Arranz P: Estudio psicometrico preliminar de la version espanola del Pediatric Inventory for Parents (PIP). International Journal of Clinical and Health Psychology 7:435-452, 2007
45. Weinshilboum RM, Raymond FA, Pazmino PA: Human erythrocyte thiopurine methyltransferase: radiochemical microassay and biochemical properties. Clin Chim Acta 85:323-333, 1978
46. Su Y, Hon YY, Chu Y, et al: Assay of 6-mercaptopurine and its metabolites in patient plasma by high-performance liquid chromatography with diode-array detection. J Chromatogr B Biomed Sci Appl 732:459-468, 1999
47. Strandbygaard U, Thomsen SF, Backer V: A daily SMS reminder increases adherence to asthma treatment: a three-month follow-up study. Respir Med 104:166-71, 2010

This model informed consent form has been reviewed by the DCT/NCI and is the official consent document for this study. Institutions should use the sections of this document which are in bold type in their entirety. Editorial changes to these sections may be made as long as they do not change information or intent. If the local IRB insists on making deletions or more substantive modifications to any of the sections in bold type, they must be justified in writing by the investigator at the time of the institutional audit.

SAMPLE INFORMED CONSENT/PARENTAL PERMISSION FOR PARTICIPATION IN RESEARCH

ACCL1033: A Comprehensive Approach to Improve Medication Adherence in Pediatric ALL

If you are a parent or legal guardian of a child who may take part in this study, permission from you is required. The assent (agreement) of your child may also be required. When we say "you" in this consent form, we mean you or your child; "we" means the doctors and other staff.

Why am I being invited to take part in this study?

You are being asked to take part in this research study because you have been diagnosed with acute lymphoblastic leukemia (ALL) and you are currently receiving maintenance therapy that includes taking a drug called 6MP by mouth.

This study is called a clinical trial. A clinical trial is a research study involving treatment of a disease in human patients. This study is organized by Children's Oncology Group (COG). COG is an international research group that conducts clinical trials for children with cancer. More than 200 hospitals in North America, Australia, New Zealand, and Europe are members of COG.

It is common to enroll children and adolescents with cancer in a clinical trial that seeks to improve cancer treatment over time. Clinical trials include only people who choose to take part. You have a choice between taking part in this clinical trial and not taking part in this trial.

Please take your time to make your decision. You may want to discuss it with your friends and family. We encourage parents to include their child in the discussion and decision to the extent that the child is able to understand and take part.

Why is this study being done?

The overall goal of this study is to compare the effects, good and/or bad, of a multi-component intervention designed to help people with ALL take their maintenance medication as directed, with the effects of an educational program. We hope to find out whether the intervention helps people to take their medication better than those receiving only education. In this study, you will get either the multi-component intervention or you will be asked to watch an educational video. You will not get both.

The use of the multi-component intervention is experimental.

What will happen on this study that is research?

If you agree to take part in this study, you will be given a Patient Supply Kit. The Supply Kit contains a special medication bottle (MEMS). You will be asked to have all 6MP prescriptions filled into the MEMS medication bottle throughout the study. The MEMS medication bottle will be fitted with a special cap (TrackCap™ CR) that electronically records when the medication bottle is opened throughout the study. When you come back to clinic with your 6MP prescription filled in the MEMS bottle, you will be assigned to 1 of 2 intervention plans. The 2 intervention plans are called Arm IP and Arm EDU, as follows:

- **Arm IP: Children/teens will receive multimedia interactive patient education (parents and patients ≥ age 12 years), a customized medication schedule, medication reminders (cell phone text messages – parents and patients ≥ age 12 years) and parent/caregiver training to supervise the child or teen's taking of medication in addition to usual care (whatever is normally done at their clinic to help people with taking their medications as directed)**

- **Arm EDU: Children/teens will receive multimedia interactive patient education (parents and patients ≥ age 12 years) in addition to usual care (whatever is normally done at their clinic to help people with taking their medications as directed)**

Random Assignment
The intervention plan that you receive is decided by a process called randomization. Randomization means that the intervention is assigned based on chance. It is a lot like flipping a coin, except that it is done by computer. You and your doctor will not pick which intervention you get. The randomization process is described in the COG Family Handbook for Children with Cancer.

Some children and teens will be randomized to receive the education and reminder intervention (IP); others will be randomized to receive education only (EDU).

Taking part in this study will not change the Maintenance therapy you are currently receiving.

Diagram of Study

This chart shows the interventions on this study.

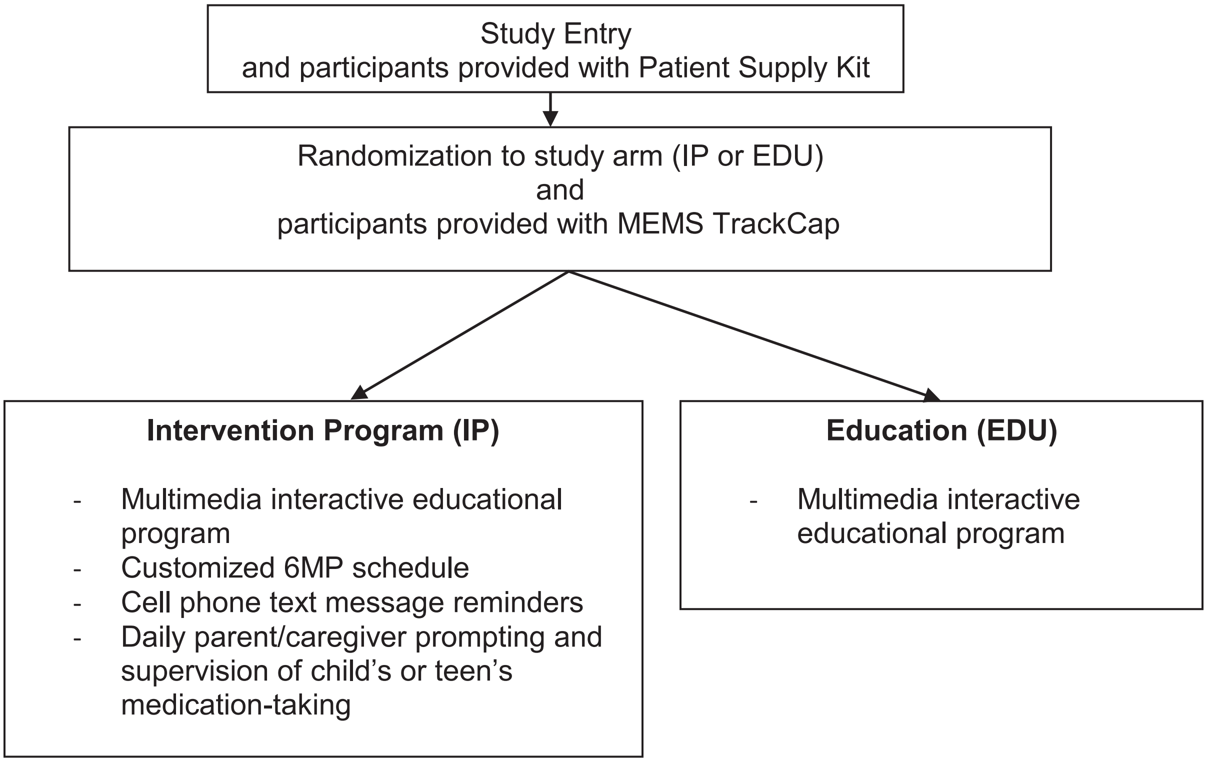

Study activities for all participants

Clinic visit #1

When you agree to be on this study, you will receive a Patient Supply Kit that contains special medication bottles (called MEMS) for your pharmacist to use when you get refills of your 6MP medication. The special MEMS bottles will have a regular cap. We will give you written instructions for you to use, and for you to give to your pharmacist when you get your refills. You will then start using the special MEMS bottle with the regular cap to take your 6MP until your next visit to the clinic.

Clinic visit #2

When you come to your next clinic visit (usually your next monthly appointment for vincristine) you will be given a special cap for the MEMS medication bottle, called a MEMS TrackCap™ CR. The TrackCap records the time and date of each opening and closing of the medication bottle. You will use the TrackCap and MEMS medication bottle when you take each dose of 6MP during the time you are in this study.

At various times during this study, you will be asked to complete 9 to 11 questionnaires (how many depends on your ethnicity). We would also like to collect blood samples every month for a total of 6 samples during the study. The questionnaires and blood samples are described in detail below.

Study activities for participants on Arm IP

If you are randomized to Arm IP, the following things will happen:

Clinic visit #3

You (if 12 years of age or older) and your parent/caregiver will be asked to watch an interactive program (DVD or on the Internet) during your regularly scheduled clinic visit. The program will take about 20-25 minutes to watch, and can be viewed in English or Spanish. The program discusses what Maintenance therapy is and why it is important, the reasons for taking 6MP, problems people face in taking medications as directed, and provides video clips of patients and parents talking about their experiences with taking 6MP. If your parent/caregiver cannot be at this clinic visit, a packet will be sent home with you that contains the DVD and instructions about the activities they should do while you are on this study.

You (if 12 years of age or older) and your parent/caregiver will each also be given a cell phone to use during the study if you do not have one or if your cell phone has limited or no text messaging. You will also be given a 6MP medication schedule that has been specially created for you. This schedule can be either in English or in Spanish. You (if 12 years of age or older) and your parent/caregiver will also start to receive text messages each time your 6MP is due to remind you to take your medication.

Parent(s)/caregiver(s) will receive a short training session to prompt and watch you take your 6MP medication.

When you (if 12 years of age or older) and/or your parent/caregiver receive the text message reminders, your parent/caregiver will prompt you as needed to take your 6MP medication and will watch you take your 6MP dose. After each dose is taken, both you (if 12 years of age or older) and/or your parent/caregiver will answer the text message indicating the dose was taken.

Clinic visits #4 through #6

At every monthly scheduled clinic visit, you will receive a monthly medication schedule tailored to you. You (if 12 years of age or older) and/or your parent/caregiver will continue to receive text message reminders to take your 6MP medication and will be asked to reply to them, as described above.

Clinic visit #7

At this clinic visit, you will return the MEMS TrackCap, and any cell phone(s) you and/or your parent were given to use during the study.

Study activities for participants on Arm EDU

If you are assigned to Arm EDU, the following things will happen:

Clinic visit #3

You (if 12 years of age or older) and your parent/caregiver will be asked to watch an interactive program (DVD or on the Internet) during your regularly scheduled clinic visit. The program will take about 20-25 minutes to watch, and can be viewed in English or Spanish. The program discusses what Maintenance therapy is and why it is important, the reasons for taking 6MP, problems people face in taking medications as directed, and provides video clips of patients and parents talking about their experiences with taking 6MP. If your parent/caregiver cannot be at this clinic visit, a packet will be sent home with you that contains the DVD.

Clinic visit #4 through #7
You will continue to receive care as usual at your clinic, during your scheduled monthly visits. On the last clinic visit for this study (#7), you will return the MEMS TrackCap.

Required Research Study Tests and Measurements
The following tests will be done because you are part of this study. If you were not in the study you would probably not have these tests. There should be no extra visits because of this study.

Blood tests for TPMT phenotyping and 6MP levels
For all children and teens participating in this study, we would like to collect blood at 6 times during the study. Blood samples will be collected on days when you are scheduled to return to the clinic for maintenance therapy (IV vincristine and steroid pulses). You will already be scheduled to have blood drawn on these days, so there would be no extra needle sticks.

We would like to take blood samples at the following times you are on this study:

- Clinic visit #2 – to test for how your body handles 6MP (TPMT phenotyping)
- Clinic visits #3 through #7 – to measure levels of 6MP in your blood

For each of the blood samples listed above, we will collect 5 mL (about 1 teaspoon) of blood. The information learned will not change the way you are treated, and the results of these tests will not be given to you.

Research Measurements
The following measurements will be done because you are taking part in this study. These measurements are not part of standard care.

We will ask you and your parent/caregiver to complete several questionnaires at several times during the time you are on this study.

We will ask all study participants to complete the following questionnaires:

- Demographic Questionnaire: We will ask you a few questions about you and your family at clinic visit #2 for the study (on the day you receive the MEMS® *TrackCap*™). If you are under age 18, your parent/caregiver will complete this questionnaire. The questionnaire will take about 5 minutes to complete.

- How You Are Feeling Questionnaire: We will ask you (if 12 years of age or older) and your parent/caregiver questions about how you are feeling at clinic visit #2 for the study. If you are under age 12, your parent/caregiver will complete this questionnaire. The questionnaire will take about 5 minutes to complete.

- Full Medication Questionnaire: We will ask you (if 12 years of age or older) and your parent/caregiver questions about your medication-taking, including questions about your current health status, knowledge about your disease and treatment plan, doses you take, reasons for missed doses, use of the MEMS cap, your confidence in taking medication as directed, problems you may have in taking medication as directed, and about any help you may get from family members for taking medication as directed. The questionnaire will take about 15 minutes to complete. We will ask you (if 12 years of age or older) and your parent/caregiver to complete this questionnaire at three times during the study: at clinic visit #2, visit #4, and at your last clinic visit for this study (visit #7).

- Short Medication Questionnaire: We will ask you (if 12 years of age or older) and your parent/caregiver some of the same questions included in the Full Medication Questionnaire, including questions about the number of days you took 6 MP over the prior 4 weeks, reasons for any missed doses, use of the MEMS cap, and about any changes in your health. This questionnaire will take about 5 minutes to complete. We will ask you (if 12 years of age or older) and your parent/caregiver to complete this questionnaire at study clinic visits #3, #5, and #6.

Additional Questionnaire for parent/caregivers ONLY:
We will also ask your parent/caregiver to complete a parental stress questionnaire during clinic visit #5. This questionnaire will ask about take about 10 minutes to complete.

Additional Questionnaire for participants on the EDU arm ONLY:
We will also ask you (if 12 years of age or older) and your parent/caregiver to complete the following at clinic visit #7 for the study:

- Education Program Questionnaire: We will ask questions about the education program and how helpful you felt it was. This questionnaire will take about 5 minutes to complete.

Additional Questionnaire for participants on the IP arm ONLY:
We will also ask you (if 12 years of age or older) and your parent/caregiver to complete the following at clinic visit #7 for the study:

- Intervention Questionnaire: We will ask you questions about the Intervention program and how helpful you felt it was. This questionnaire will take about 5 minutes to complete.

Additional Questionnaire for participants who are Hispanic:
We will also ask you (if 12 years of age or older) and your parent/caregiver to complete the following at clinic visit #2 for the study:

- Hispanic Acculturation Questionnaire: We will ask you and your parent/caregiver questions such as what language you usually use to read, speak and think in; in what language you prefer to watch television and listen to the radio; and what language your friends use. This questionnaire will take about 5 minutes to complete.

Additional Questionnaire for participants who are Asian:
We will also ask you (if 12 years of age or older) and your parent/caregiver to complete the following at clinic visit #2 for the study:

- Asian Acculturation Questionnaire: We will ask you and your parent/caregiver questions such as what language you usually use to read, speak and think in; in what language you prefer to watch television and listen to the radio; and what language your friends use. This questionnaire will take about 5 minutes to complete.

What side effects or risks can I expect from being in the study?

Risks for all participants on study:
The risks of this study are minor. Some of the questions in the questionnaires are of a personal nature and may make you feel uncomfortable. The questionnaires that are being used for this research study have been used many times by many hospitals and clinics. To the best of our knowledge they have not caused anyone serious problems. You do not have to answer any question that you don't want to.

Blood drawing may cause pain, bruising, bleeding, or infection at the site of the needle stick; however, blood tests will be done for your usual medical care whether or not you participate in this study. The study-related blood tests will be in the form of an extra tube, with no addition need sticks, and there are no additional risks related to blood drawing associated with participating in this study beyond those experienced during usual medical care. Care will be taken to avoid these complications.

Additional Risks for participants on the EDU arm:
In addition to the risks above, the risks associated with viewing the educational video are considered minor, and may include discomfort in taking part in this activity.

Additional Risks for participants on the IP arm:
In addition to the risks above, the risks associated with viewing the educational video, and receiving and replying to text messages are considered minor, and may include discomfort in taking part in one or more of these activities.

In addition to the risks described above, there may be unknown risks, or risks that we did not anticipate, associated with being in this study.

Reproductive risks
There are no reproductive risks associated with being on this study.

Are there benefits to taking part in the study?

We hope that this study will help you personally, but we do not know if it will.

Taking part in this study may or may not help you be better able to take your medication as directed. While study doctors hope the educational video and multi-component Intervention Program will be useful in helping people take medications as directed, there is no proof of this yet. We expect that the information learned from this study will benefit other patients in the future.

What other options are there?

Instead of being in this study, you can choose not to take part in this study.

How many people will take part in the study?

The total number of people enrolled on this study is expected to be 608.

How long is the study?

People in this clinical trial are expected to be on this study for about 6 months. After you are finished with the study activities, you will not have any follow-up examinations or medical tests for this study. However, we would like to continue to collect some information about how you are doing for as long as you are willing to let us. The researchers handling this study at your hospital will collect this information by looking at your medical record, or if you have not visited

the hospital recently, by calling you on the telephone once a year to see how you are doing. If they need to call you on the telephone, the call would last about 5 minutes.

You can stop taking part in the study at any time. However, if you decide to stop participating in the study, we encourage you to talk to the study doctor and your regular doctor first.

Your doctor or the study doctor may decide to take you off this study:
- if he/she believes that it is in your best interest
- if your disease comes back while you are participating in this study

What about privacy?

We will do our best to make sure that the personal information in your medical record will be kept private. However, we cannot guarantee total privacy. Your personal information may be given out if required by law. If information from this study is published or presented at scientific meetings, your name and other personal information will not be used.

The Children's Oncology Group has received a Certificate of Confidentiality from the federal government, which will help us protect the privacy of our research participants. Information about the certificate is included in **Attachment #1**.

Organizations that may look at and/or copy your research or medical records for research, quality assurance and data analysis include groups such as:

- **Children's Oncology Group**
- **Representatives of the National Cancer Institute (NCI), Food and Drug Administration (FDA), and other U.S. regulatory agencies involved in overseeing research**
- **The Institutional Review Board of this hospital**
- **The Pediatric Central Institutional Review Board (CIRB) of the National Cancer Institute**
- **The Institutional Review Board of Children's of Alabama/University of Alabama at Birmingham (the location of the study coordinating center)**
- **Children's of Alabama/University of Alabama at Birmingham (UAB), as the coordinating center, will receive the research information including personal information**

What are the costs?

Taking part in this study may lead to added costs to you. If you are assigned to Arm IP and choose to use your own cellular telephone, it is possible that you may be charged by your phone company for sending or receiving study-related text messages (if your cell phone has limited or no text messaging services). **To avoid these charges, you have the option of using a study cell phone, which will be supplied to you free of charge, for use throughout the time that you are participating in this study.** You or your insurance company will be charged for continuing medical care and/or hospitalization. There are no plans for the study to pay for medical treatment.

Please ask about any expected added costs or insurance problems. Staff will be able to assist you with this.

In the case of injury or illness resulting from this study, emergency medical treatment is available but will be provided at the usual charge. No funds have been set aside to compensate you in the event of injury. However by signing this form, you are not giving up any legal rights to seek to obtain compensation for injury.

For more information on clinical trials and insurance coverage, you can visit the National Cancer Institute's Web site at http://cancer.gov/clinicaltrials/understanding/insurance-coverage. You can print a copy of the "Clinical Trials and Insurance Coverage" information from this Web site.

Funding support

If you choose to enroll on this study, this institution will receive some money from the Children's Oncology Group to do the research. There are no plans to pay you for taking part in this study.

This study includes providing specimens to the researcher; there are no plans for you to profit from any new product developed from research done on your specimens.

What are my rights as a participant?

Taking part in this study is voluntary. You may choose not to be in this study. If you decide not to be in this study, you will not be penalized and you will not lose any benefits to which you are entitled. You will still receive medical care.

You can decide to stop being in the study at any time. Leaving the study will not result in any penalty or loss of benefits to which you are entitled. Your doctor will still take care of you.

We will tell you about new information that may affect your health, welfare, or willingness to stay in this study. A committee outside of COG closely monitors study reports and notifies COG if changes must be made to the study. Members of COG meet twice a year to discuss results of treatment and to plan new treatments.

You may ask to be given a summary of the study results after they are written up. This may be several years from now since all people on the study need to have finished taking part in this study.

Whom do I call if I have questions or problems?

For questions about the study or if you have a research related problem or if you think you have been injured in this study, you may contact Dr. *XXXX* or your doctor at *XXXX*.

If you have any questions about your rights as a research participant or any problems that you feel you cannot discuss with the investigators, you may call *XXXX* IRB Administrator at *XXXX*.

If you have any questions or concerns that you feel you would like to discuss with someone who is not on the research team, you may also call the Patient Advocate at *XXXX*.

Where can I get more information?

The COG Family Handbook for Children with Cancer has information about specific cancers, tests, treatment side effects and their management, adjusting to cancer, and resources. Your doctor can get you this Handbook, or you can get it at www.childrensoncologygroup.org/familyhandbook .

Visit the NCI's Web site at http://www.cancer.gov.

If you are in the United States, you may call the NCI's *Cancer Information Service* at:
1-800-4-CANCER (1-800-422-6237) or TTY: 1-800-332-8615.

Information about long term follow-up after cancer treatment can be found at: http://www.survivorshipguidelines.org/.

A description of this clinical trial will be available on http://www.ClinicalTrials.gov, as required by U.S. Law. This Web site will not include information that can identify you. At most, the Web site will include a summary of the results. You can search this Web site at any time.

You will get a copy of this form. You may also ask for a copy of the protocol (full study plan).

Signature

I have been given a copy of all _____ pages of this form. The form includes one (1) attachment.

I have reviewed the information and have had my questions answered.
I agree to take part in this study.

Participant____Date ____________

Parent/Guardian____Date ____________

Parent/Guardian____Date ____________

Physician/PNP obtaining consent______________________________Date ____________
IRB# _______________ IRB Approved: ___________

Attachment #1
Certificate of Confidentiality

The Children's Oncology Group has received a Certificate of Confidentiality from the federal government, which will help us protect the privacy of our research subjects. The Certificate protects against the involuntary release of information about subjects collected during the course of our covered studies. The researchers involved in the studies cannot be forced to disclose the identity or any information collected in the study in any legal proceedings at the federal, state, or local level, regardless of whether they are criminal, administrative, or legislative proceedings. However, the subject or the researcher may choose to voluntarily disclose the protected information under certain circumstances. For example, if the subject or his/her guardian requests the release of information in writing, the Certificate does not protect against that voluntary disclosure. Furthermore, federal agencies may review our records under limited circumstances, such as a DHHS request for information for an audit or program evaluation or an FDA request under the Food, Drug and Cosmetics Act. The Certificate of Confidentiality will not protect against the required reporting by hospital staff of information on suspected child abuse, reportable communicable diseases, and/or possible threat of harm to self or others.

CHILDREN'S ONCOLOGY GROUP

The world's childhood cancer experts

Group Chair
Peter C. Adamson, M.D.
adamson@email.chop.edu

Group Vice Chair
Susan Blaney, M.D.
smblaney@txch.org

Chief Operating Officer
Elizabeth O'Connor, M.P.H.
econnor@childrensoncology group.org

Chief Administrative Officer
Maria Hendricks, M.S.N, R.N. C.C.R.P.
hendricksm@email.chop.edu

Group Statistician
James Anderson, Ph.D.
janderson@unmc.edu

Associate Group Statistician
Mark Krailo, Ph.D.
mkrailo@childrensoncology group.org

Group Chair's Office
The Children's Hospital of Philadelphia
3501 Civic Center Blvd
CTRB 10060
Philadelphia, PA 19104

P 215 590 6359
F 215 590 7544

Group Operations Center
222 E. Huntington Drive
Suite 100
Monrovia, CA 91016

P 626 447 0064
F 626 445 4334

Statistics & Data Center Headquarters
222 E. Huntington Drive
Suite 100
Monrovia, CA 91016

P 626 447 0064
P 626 445 4334

Gainesville Office
COG Statistics and Data Center
University of Florida
6011 NW 1st Place
Gainesville, FL 32607

P 352 273 0556
F 352 392 8162

Omaha Office
University of Nebraska Medical Center
984375 Nebraska Medical Center
Omaha, NE 68198 4375

P 402 559 4112
F 402 559 7259

A National Cancer Institute supported clinical cooperative group and Equal Opportunity Affirmative Action Institutions

Memo

To: Principal Investigators and Clinical Research Associates

From: Lanie Lindenfeld, Protocol Coordinator

Re: **ACCL1033** Amendment #1

Date: Monday, January 14, 2013

X	**AMENDMENT (#1)**	____	**STATUS CHANGE**
X	Change of Participants/Coordinator(s)	____	Closure
X	Editorial or Administrative Changes	____	Partial Closure
____	Scientific Changes*	____	Temporary Closure
____	Therapy Changes*	____	Reactivation
X	Eligibility Changes*		
X	Informed Consent Changes*		

***FOR THE PURPOSE OF INSTITUTIONAL PERFORMANCE ASSESSMENT, THIS AMENDMENT WILL REQUIRE SUBMISSION TO AND APPROVAL BY IRBs WITHIN 90 DAYS.**

Below is a description of amendment #1 for **ACCL1033,** *A Comprehensive Approach to Improve Medication Adherence in Pediatric ALL*. The main goals of the amendment are summarized below, followed by details of all the changes.

1. The study will be open to all race/ethnicities and age groups, since the intervention has the potential to improve adherence in all populations.

- Inclusion of Asian children and patients of other racial/ ethnic backgrounds (including patients of mixed racial/ ethnic backgrounds)
- Inclusion of younger children (patients <12 years at study entry will be eligible as long as age at diagnosis was ≥ 1 year); only the parents will complete the questionnaires and receive the intervention for children <12 years at study entry

Rationale: Since the opening of ACCL1033, we have completed the analysis of all the patients enrolled on AALL03N1 (from the 4 racial/ ethnic groups). The findings from the analysis demonstrate that Asians (along with Hispanics, and African Americans) have significantly lower adherence when compared with non-Hispanic whites; and that younger patients (<12) also have a substantial number of non-adherers who would benefit from the intervention. These findings form the basis for inclusion of Asians into the

study. Furthermore, we believe that the intervention will benefit ALL patients across all ages and racial/ ethnic groups, and are therefore proposing inclusion of patients of other race/ethnicities (including patients of mixed racial/ ethnic backgrounds) with this Amendment. Younger children (patients < 12 years at study entry) will be eligible as long as age at diagnosis was ≥ 1 year. For children <12 years at study entry, the intervention will be directed at the designated parent/ caregiver (the designated parent/ caregiver will be responsible for completing the questionnaires).

2. **The Usual Care group will now view the educational video (MIPE program)**
   - ~~The Usual Care (UC)~~ arm is now called the **Education Only arm (EDU)**
   - EDU arm will also view the Multimedia Interactive Patient Education video on Day 29
   - The EDU arm will also be compared to patients who participated in AALL03N1 (historical control) to understand the impact of the educational program

Rationale: Given the high prevalence of non-adherence among children with ALL, we believe that no group should go un-intervened. It is for this reason that we will now have one group (the original Usual Care group) view the educational video (now called EDU), so that all participants may potentially benefit from participating in the study. The adherence rates in the EDU arm will be compared with those from the AALL03N1 study (historical controls), to understand the impact of the educational program.

3. **The study aims were revised:**

Primary Aim: Determine the impact of interventions proposed in IP vs. EDU on adherence to oral 6MP in children with ALL. Adherence will be measured by: i) MEMS (primary measure of adherence to oral 6MP, providing real-time data; ii) red cell TGN levels (providing data on chronic, systemic 6MP exposure)

Secondary Aim 1: Determine the impact of the education program on adherence to 6MP by comparing patients on EDU to an un-intervened historical control (HC) group drawn from the COG AALL03N1 study

Secondary Aim 2: Examine the modifying effect of sociodemographic characteristics, health beliefs/ knowledge, and degree of acculturation on changes in adherence with the IP (IP vs. EDU; and EDU vs. HC).

4. **With the revision in the eligibility criteria and study aims, the statistical analysis section and sample size have been modified. The target accrual has increased from 400 to 570 participants. Detailed analytic plan is described in detail below and in Section 5 (pages 26-28).**

5. **Other changes include:**
   a. For patients who travel between more than one household, only one designated parent/ caregiver will be selected for the study.
   b. ~~The Directly Observed Therapy (DOT)~~ component of the intervention has been renamed **"Directly Supervised Therapy (DST)".** If the patient is not physically present with the

designated parent/caregiver at the time the 6MP dose is prescribed, the designated parent/caregiver can call the patient (for patients ≥12 years at study participation) or alternate caregiver (for patients < 12 years) to make sure that the 6MP dose was taken.

c. After participants complete the ACCL1033 EDU or IP arm, we will request the completion of follow-up forms to document patients that relapse, develop a SMN, or expire, in order to demonstrate that the intervention has an impact on outcome (i.e., relapse risk). These follow-up forms will be completed every 6 months, for a total of 5 years, and then annually until 10 years from diagnosis.

d. It has come to our attention that there may be other intervention studies designed to improve adherence in pediatric oncology patients. Patients who have participated in these types of studies will be excluded from ACCL1033.

e. Other minor changes were made to the protocol, as described below.

All changes to the protocol and informed consent are detailed below. The inserted text appears in ***italics and bold*** and text that has been removed from the protocol appears with a ~~strike through~~.

GLOBAL CHANGES THROUGHOUT PROTOCOL, CONSENT

- ~~Usual Care (UC)~~ now called **Education Only (EDU)**
- ~~Directly Observed Therapy (DOT)~~ now called **Directly Supervised Therapy (DST)**

PROTOCOL CHANGES

Title Page

- Updated the amendment number and version date.

Table of Contents (page 2)

- Repaginated the Table of Contents as needed
- Added Sections 4.4 and 4.5 (previously omitted)

Study Committee (pages 3 and 4)

- Updated the study committee membership and contact information as needed.

Abstract (pages 5 and 6)

- Updated results from AALL03N1 to include preliminary results from all race/ethnicities:

We have completed enrollment for this study, and preliminary results are presented here. 462 patients (168 Hispanics; 157 non-Hispanic whites; 69 Asians; 68 African Americans) yielded 76,055 person-days of adherence data. Median age at participation was 6 years (2-20); 67% were males; 40% had high-risk disease per NCI criteria; 61% reported income <$50k/year; 14% reported single-caregiver households. Among patients with normal TPMT activity, each 1% increase in MEMS-based adherence was accompanied by a 14 unit (pmol/$8\cdot10^8$ red cells) increase in TGN (p=0.01). Multivariate longitudinal analysis revealed adherence to be significantly lower in adolescents (≥12y: 84.5% vs. <12y: 92.6%, p=0.0003); patients from single-caregiver households (87.2% vs. 92.0%, p=0.03); patients with low income (<$50k/y: 89.4% vs. ≥$50k/y: 93.8%, p=0.02); and Hispanics (90.5±1.6%), Asians (85.3±3.7%) and African Americans (85.3±2.9%) compared with non-Hispanic whites (95.3±1.2%, p<0.0001). Reasons for missing 6MP included forgetfulness (79%), logistical barriers (19%), and active refusal (2%). After a median follow-up of 5.4 years, multivariate analysis (adjusting for clinical/sociodemographic factors) revealed that adherence <95% was associated with an increase in relapse risk (reference: adherence ≥95%; 94.9%-90%: Hazard Ratio [HR]=3.3, 95% Confidence Interval [CI], 1.0-11.6, p=0.06; 89.9%-85%: HR=3.4, 95%CI, 0.9-13.0, p=0.07;

<85%: HR=4.5, 95%CI, 1.3-15.1, p=0.02), leading us to use <95% as the cut-point for adherence with a clinically unacceptable increase in relapse. Using this definition, 45% of the patients were non-adherers. The cumulative incidence of relapse was significantly higher among non-adherers (18.8% vs. 4.9%, p=0.0003). Furthermore, non-adherers were at a 3.7-fold increased risk of relapse (95%CI, 1.4-10.2, p=0.01), after adjusting for sociodemographic/clinical variables. The adjusted risk of relapse attributable to non-adherence was 47% for this cohort that had entered maintenance in first clinical remission (Bhatia S. personal communication).

~~We have completed enrollment for the Hispanics and non-Hispanic whites; preliminary results are presented here. After 53,394 person-days of observation, the adherence rate declined from 94.7% (Month 1) to 90.2% (Month 6) (p<0.001). The adherence rate was significantly lower among Hispanics (88.4%) compared with non-Hispanic whites (94.8%, p<0.001), among older patients (age ≥ 12 years, p<0.001), and among patients from single-mother households (p=0.001). The cumulative incidence of relapse was significantly higher among Hispanics (16.5%±4.0% at 4 years from study entry) compared to non-Hispanic whites (6.3%±2.2%, p=0.02) After adjusting for sex, NCI risk classification, chromosomal abnormalities, 6MP dose intensity, TPMT activity, ethnicity, time from diagnosis to adherence study entry, parental education and household income, there was a progressive increase in risk of relapse with decreasing levels of adherence (90%-94.9%: HR=4.1, p=0.02; 85%-89.9%: HR=4.0, p=0.04; <85%: HR=5.7, p=0.002; referent group: adherence rate ≥ 95%). More importantly, the association between ethnicity and recurrence or death was mitigated in size and lost its significance (HR=1.8, p=0.26), after accounting for adherence, parental education, and income (Bhatia S. personal communication).~~ Study results suggest that lower adherence to oral 6MP influences risk of relapse, and along with socioeconomic status, contributes to the ethnic differences in disease outcome observed in children with ALL. ~~The study continues to enroll African-American and Asian patients to complete target enrollment.~~

- Last Sentence of Paragraph 3 (page 6):

  This investigation will test the feasibility, utility, and efficacy of a technologically sophisticated, web-based medication scheduling and text-messaging reminder system (REM) that capitalizes on the ubiquitous presence and acceptance of personal cellular phones, and prompts directly ~~observed~~ ***supervised*** therapy (~~DOT~~ ***DST***) of each dose by a ***designated*** parent/~~designated~~ caregiver, coupled with a multimedia-based interactive patient education program (MIPE), to increase adherence to daily oral 6MP in children with ALL ~~at high risk for non-adherence~~.

Experimental Design Schema (page 7)

- The schema has been updated to reflect the changes in this amendment. See the tracked changes version of the amendment for greater detail.

Section 1.0 (pages 8 and 9)

- The specific aims have been updated to reflect the revised study design and Table 1 has been added (subsequent tables have been re-numbered):

Conduct a randomized trial of an intervention program (IP) consisting of ***customized printed 6MP schedules and*** ~~customized~~ electronic reminders (cellular text messages) (REM) coupled with directly ~~observed~~ ***supervised*** therapy (~~DOT~~***DST***) and multimedia-based interactive patient education program (MIPE) ***vs. education alone (EDU)*** in children ***diagnosed*** with ALL ~~at high risk for non-adherence~~. ***For children assigned to IP who are <12 years of age at randomization, only the designated caregiver will receive text message reminders, prompting DST. For older patients (≥12 years), both the patient and designated parent/caregiver will receive text message reminders, prompting DST.***

Primary Aim~~s~~

Determine the impact of interventions proposed in IP vs. EDU on adherence to oral 6MP in children with ALL. Adherence will be measured by: i) MEMS (primary measure of adherence to oral 6MP, providing real-time data; ii) red cell TGN levels (providing data on chronic, systemic 6MP exposure)

Hypothesis 1.1: ***An intervention package consisting of customized printed schedules, text message reminders, DST and education (IP) will result in a higher proportion of individuals with adherence rates ≥95% compared with education alone (EDU)***

Hypothesis 1.2: ***A higher proportion of patients placed on IP will have adherence rates ≥95% compared with those placed on EDU, irrespective of age at study participation (<12 or ≥12 years), thus taking into account age-appropriate modifications in intervention (text message reminders sent only to parents of younger patients)***

~~1. Determine the impact of the IP on adherence to 6MP using the following adherence assessments: (1) Frequency of 6MP dosing using MEMS (primary measure of adherence); (2) 6MP dosage- and TPMT-normalized serial RBC 6TGN levels; and (3) Self-report of adherence.~~

Secondary Aims

Secondary Aim 1: ***Determine the impact of the education program on adherence to 6MP by comparing patients on EDU to an un-intervened historical control (HC) group drawn from the COG AALL03N1 study***

Hypothesis 2: ***Intervention with a multimedia interactive education program (patients on EDU) will result in higher proportion of individuals with adherence rates (≥95%) compared with those observed in the un-intervened HC group (COG AALL03N1), after adjusting for baseline sociodemographic characteristics***

Secondary Aim 2: Examine the modifying effect of socio-demographic ~~and clinical~~ characteristics, ~~as well as~~ health beliefs/ knowledge, ***and degree of acculturation*** on changes in adherence with the IP ***(IP vs. EDU; and EDU vs. HC).***

Hypothesis 3: ***Subgroups of children with ALL (characteristics shown below) will derive the most benefit from the intervention***

<u>Demographics</u>: age at study participation, race/ethnicity, number of adult caregivers in household

<u>Health beliefs/ knowledge</u>: perceived severity of illness, knowledge regarding purpose of 6MP, perception of self-efficacy to overcome barriers

<u>Acculturation:</u> degree of acculturation

Table 1. (page 9_Vulnerable sub-populations hypothesized to derive the most benefit from intervention

Vulnerable subpopulations	***Rationale for why interventions should work in these populations***
≥12 years of age	***Lack of supervision without intervention contributes to non-adherence; more likely to forget***
Single parent household	***Lack of supervision without intervention contributes to non-adherence; more likely to forget***
Underestimation of severity of illness	***Lack of understanding regarding the critical need for 6MP to sustain continuous remission***
Lack of knowledge re purpose of 6MP	***Lack of understanding regarding the critical need for 6MP to sustain continuous remission***
Low self-efficacy to overcome barriers	***Intervention would provide the necessary resources to overcome barriers***
Spanish-speaking – miscommunication	***Written instructions & text messages in Spanish to improve understanding/communication***

<u>Background and Rationale</u>

The following sections have been updated and revised:

<u>Section 2.4 (page 11)</u>

- Deleted the last sentence in first paragraph: ~~The study has completed target enrollment for Hispanics and non-Hispanic whites; enrollment for the Asians and African-Americans is ongoing until target enrollment is reached.~~
- Updated results from AALL03N1 in second paragraph:

We have completed enrollment for AALL03N1, and preliminary results are presented here. Four hundred and sixty-two patients (168 Hispanics; 157 non-Hispanic whites; 69 Asians; 68 African Americans) yielded 76,055 person-days of adherence data. Median age at participation was 6 years (2-20); 67% were males; 40% had high-risk disease per NCI

criteria; 61% reported income <$50k/year; 14% reported single-caregiver households. Among patients with normal TPMT activity, each 1% increase in MEMS-based adherence was accompanied by a 14 unit (pmol/$8\cdot10^8$ red cells) increase in TGN (p=0.01). Multivariate longitudinal analysis revealed adherence to be significantly lower in adolescents (≥12y: 84.5% vs. <12y: 92.6%, p=0.0003); patients from single-caregiver households (87.2% vs. 92.0%, p=0.03); patients with low income (<$50k/y: 89.4% vs. ≥$50k/y: 93.8%, p=0.02); and Hispanics (90.5±1.6%), Asians (85.3±3.7%) and African Americans (85.3±2.9%) compared with non-Hispanic whites (95.3±1.2%, p<0.0001). Reasons for missing 6MP included forgetfulness (79%), logistical barriers (19%), and active refusal (2%). After a median follow-up of 5.4 years, multivariate analysis (adjusting for clinical/sociodemographic factors) revealed that adherence <95% was associated with an increase in relapse risk (reference: adherence ≥95%; 94.9%-90%: Hazard Ratio [HR]=3.3, 95% Confidence Interval [CI], 1.0-11.6, p=0.06; 89.9%-85%: HR=3.4, 95%CI, 0.9-13.0, p=0.07; <85%: HR=4.5, 95%CI, 1.3-15.1, p=0.02), leading us to use <95% as the cut-point for adherence with a clinically unacceptable increase in relapse. Using this definition, 45% of the patients were non-adherers. The cumulative incidence of relapse was significantly higher among non-adherers (18.8% vs. 4.9%, p=0.0003). Furthermore, non-adherers were at a 3.7-fold increased risk of relapse (95%CI, 1.4-10.2, p=0.01), after adjusting for sociodemographic/clinical variables. The adjusted risk of relapse attributable to non-adherence was 47% for this cohort that had entered maintenance in first clinical remission. The results describing the prevalence and predictors of adherence among the Caucasians and Hispanics were recently published.[18]

~~Preliminary results were presented at the American Society of Hematology in 2008 and 2010, and have since been updated. These updated results are summarized here. A total of 158 non Hispanic white and 169 Hispanic children contributed 53,394 person-days of adherence observation in the AALL03N1 study. Adherence was monitored using MEMS caps to record date/time of 6MP bottle openings. The adherence rate declined from 94.7% (Month 1) to 90.2% (Month 6) (p<0.001). Adherence rate was significantly lower among Hispanics (88.4%) compared with non-Hispanic whites (94.8%, p<0.001), among older patients (age ≥12 years, p<0.001), and among patients from single-mother households (p=0.001). (Bhatia S, et al, Manuscript in preparation, 2011).~~

Section 2.5 (page11)

- Updated reasons for missing 6MP in first paragraph:

  In the AALL03N1 study, the most frequent reasons reported by patients/parents for missing 6MP included ***forgetfulness (79%), logistical barriers (19%), and active refusal (2%).*** ~~forgetfulness (55%), disruption of usual routine (22%), logistical barriers (15%), side-effects (5%), miscommunication (2%), and active refusal (1%).[10]~~

- Updated the second paragraph to include barriers and facilitators to adherence identified by children/teens with ALL and their parents (pages 11 and 12):

We conducted semi-structured interviews (in English and Spanish) of children with ALL and their parents to understand facilitators and barriers to adherence to oral chemotherapy. Interviews were conducted separately (but concurrently) with patients ≥12 years of age and their parents. Patients <12 years were represented in the interviews by a parent. Identified barriers fell into three groups: (1) cognitive/process-related (forgetfulness, change in normal activities, lack of organization); (2) medication-related (palatability, pill-swallowing, dietary restrictions, and side-effects), and (3) behavioral/psychological (adolescent risk-taking, and volitional refusal). Identified facilitators included knowledge/belief in efficacy of medication, clear delineation of responsibility, use of process enhancers (aides, pill boxes, and calendars), trust/confidence in healthcare providers, a positive outlook, and social/spiritual support. Although patients and parents were interviewed separately (yet simultaneously) during the study, there was remarkable concordance in key themes identified by each patient/parent pair. No culture- or ethnicity-specific barriers or facilitators were identified, despite the use of interview prompts designed to specifically elicit culturally-based beliefs and practices.

Adherent teens and their parents consistently emphasized the importance of parental responsibility as a strategy for overcoming forgetfulness; these pairs described the use of parental reminders to prompt teens to take their medications, along with parental follow-up with the teen to be certain that the doses had been taken (coded as "parental vigilance" in the qualitative study). In contrast, the non-adherent teens had difficulty identifying the person in the parent-teen dyad responsible for medication administration. Furthermore, patients who understood the consequences of not taking 6MP demonstrated adherence and reported overcoming barriers. Results suggest that adolescents with ALL may differ in regard to their need for autonomy/ independence compared to healthy adolescents. Several adolescents indicated that although they would normally find close parental supervision distasteful, they welcomed parental involvement when it came to taking their oral chemotherapy as prescribed. Findings are consistent with HBM.

~~In a qualitative study conducted to identify barriers and facilitators to adherence in children with ALL,[20] 38 semi-structured interviews of Hispanic and non Hispanic white children/teens with ALL and their parents were conducted. Facilitators to adherence included delineation of roles and responsibilities for medication taking between parent and child, **parental vigilance**, and **use of reminder systems**. Barriers included **forgetfulness**, lack of organization, **getting busy with other things**, taking a passive role in treatment, **not developing a routine for taking medications**, difficulty swallowing pills, confusion regarding instructions for taking medications, and **lack of parental supervision of medication taking**. Children with an understanding of the association between taking oral chemotherapy and control of disease demonstrated adherent behaviors and reported overcoming barriers or taking medication despite barriers that could not be overcome. Children who lacked this understanding reported non adherent behaviors and consistently cited barriers for not taking prescribed medication. Findings are consistent with Health Belief and Self-Efficacy Models, where adherence behaviors are determined by perceived susceptibility to and severity of illness weighed against perceived benefits of and barriers to treatment regimens, coupled with self-belief in the capability to perform required actions needed to achieve adherence.~~

Section 2.6 (page12)

- Updated results from AALL03N1 in second paragraph and deleted the last paragraph:

Using the AALL03N1 data, we attempted to identify a clinically relevant level of adherence. After a median follow-up of 5.4 years, multivariate analysis (adjusting for clinical/sociodemographic factors) revealed that adherence <95% was associated with an increase in relapse risk (reference: adherence ≥95%; 94.9%-90%: Hazard Ratio [HR]=3.3, 95% Confidence Interval [CI], 1.0-11.6, p=0.06; 89.9%-85%: HR=3.4, 95%CI, 0.9-13.0, p=0.07; <85%: HR=4.5, 95%CI, 1.3-15.1, p=0.02), leading us to use <95% as the cut-point for adherence with a clinically unacceptable increase in relapse. Using this definition, 45% of the patients were non-adherers. The cumulative incidence of relapse was significantly higher among non-adherers (18.8% vs. 4.9%, p=0.0003). Furthermore, non-adherers were at a 3.7-fold increased risk of relapse (95%CI, 1.4-10.2, p=0.01), after adjusting for sociodemographic/clinical variables. The adjusted risk of relapse attributable to non-adherence was 47% for this cohort that had entered maintenance in first clinical remission (Bhatia S. personal communication).

~~Using data from AALL03N1 we have demonstrated that the median adherence rate was 88.2% among those who experienced a recurrence; significantly lower than among those who remained in continuous complete remission (96.2%, p=0.001). The cumulative incidence of relapse for the entire cohort was 11.0%±2.1% at 4 years from adherence study entry. After adjusting for sex, NCI risk classification, chromosomal abnormalities, 6MP dose intensity, TPMT activity, ethnicity, time from diagnosis to adherence study entry, parental education and household income, there was a progressive increase in risk of relapse with decreasing levels of adherence (90%-94.9%: HR=4.1, p=0.02; 85%-89.9%: HR=4.0, p=0.04; <85%: HR=5.7, p=0.002; referent group: adherence rate ≥95%.. Association between ethnicity and recurrence/death (HR=2.6, p=0.02) became non-significant (HR=1.8, p=0.26), after accounting for adherence, parental education and income (Bhatia, personal communication).~~

~~Using the AALL03N1 data, we attempted to identify a clinically relevant level of adherence. We have identified those patients with adherence levels < 95% are at increased risk of relapse. Using < 95% adherence rate as the definition of non-adherence, we determined that 44% of children with ALL were non-adherent. Finally, non-adherent patients had significantly higher risk cumulative incidence of relapse compared with adherent patients (17.0%±3.7% vs. 4.9%±1.9%, p=0.001).~~

Section 2.7.1 (13)

- ~~Directly Observed Therapy (DOT)~~ replaced with ***Directly Supervised Therapy (DST)***

Section 2.8 (page 14)

- Updated results from AALL03N1 in first paragraph:

  Results from AALL03N1 demonstrate that ~~44~~***45***% of children with ALL are consuming <95% of prescribed 6MP (non-adherers), and that the prevalence of non-adherers is significantly higher among Hispanic***, Asians, and African Americans.*** ~~adolescents (79%).~~
- ~~Directly Observed Therapy (DOT)~~ replaced with ***Directly Supervised Therapy (DST)***
- Revised part of last sentence in section:

The study will test the hypothesis that … in the setting of directly observed ***supervised*** therapy will significantly enhance adherence to oral 6MP ***compared to education alone***. among those identified to be at high risk.

Section 3.1.4 (page 15)

- Updated randomization since patients will be stratified by ethnic background and age:

Randomization, stratified by ethnic background (3 ***5*** strata: Hispanic, non-Hispanic white, and African-American***, Asian, and Other***) ***and age at randomization (2 strata: < 12 years, 12 years and older)***, will take place at the time a patient is enrolled on study via the Eligibility eRDE Form. The African-American stratum will remain closed until accrual goals on COG study AALL03N1 have been met, at which time the stratum will be opened to enable enrollment of African-American patients. Patients will be assigned to either the Intervention Program (IP) ***arm*** or Usual Care ***Education Only*** (UC***EDU***) arm (see Section 5.1 for details).

Section 3.2 (page 15)

- The eligibility criteria have been revised to include younger age groups and there is no longer a race/ethnicity requirement. Patients who are enrolled or previously participated in another intervention clinical trial designed to improve adherence will be excluded:

3.2.1 Inclusion Criteria

(1) Diagnosis of ALL ***at ≥ 1 year and*** ≤ 21 years of age, in first remission. Enrollment on a COG therapeutic study for ALL is not required.

(2) Belonging to the following self-reported racial/ethnic categories: (a) non-Hispanic white/Caucasian or (b) Hispanic, or (c) African-American. Below are definitions for these categories:

Non-Hispanic white/Caucasian:
Includes white or light-skinned patients of European, North African, or Middle Eastern ancestry

Hispanic:
Patients of Hispanic ethnicity include Mexican, Mexican-American, Chicano, Cuban, Puerto Rican, or other Hispanic/Spanish/Latino ethnicities

African American:
Includes patients who are African-American or of sub-Saharan black African ancestry

Please note that patients of multi-ethnic/multi-racial backgrounds are not eligible for this study. While patients of multi-ethnic/multi-racial ancestry (e.g., Caucasian/African-American, Hawaiian/Puerto Rican) are not eligible, patients of mixed ancestry within a race/ethnicity (e.g., Mexican/Puerto Rican = Hispanic) may participate as long as they fall under the general classification of "Caucasian," "African-American," or "Hispanic."

(2) Age ≥ 12 years and ≤ 25 years at the time of study enrollment

(3) Has completed at least 24 weeks (6 months) of maintenance chemotherapy, and is scheduled to receive at least 6 more months of maintenance chemotherapy

(4) Receiving oral 6MP during the maintenance phase of therapy for ALL

(5) Has a ***designated*** parent or designated caregiver (i.e., adult physically present in the household) who is willing to enter into a mutual agreement with the patient to participate in a daily supervised medication administration routine. For patients who travel between m*ore than* one household, a parent or designated caregiver will be required at each household.

(6) Able and willing to use the MEMS® *TrackCap*™ (e.g., not using a pillbox or prescribed liquid 6MP)

(7) Patient and p ***P***arent/caregiver ***and patient (if 12 years and older)*** must be willing to use a cellular telephone to receive medication reminders via text messaging during study period

(8) Patient and parent/caregiver must speak English or Spanish

3.2.2 Exclusion Criteria

(1) Patients with Down syndrome (due to excessive toxicity during Maintenance on historical trials, patients with Down syndrome now receive reduced Maintenance duration and vincristine/steroid pulses frequency [every 12

weeks rather than every 4 weeks]; thus Maintenance therapy for Down syndrome patients is not comparable to standard Maintenance therapy for high-risk ALL)

(2) Patients who previously participated in or are currently participating in another intervention clinical trial designed to improve adherence

Section 4.1.1 (page 16)

- Revised the following sentences in paragraph 1, so that Study Participation worksheets are due every 3 months and all participants will watch the MIPE video:
    - All eligible patients and their ***designated*** parents/ ~~designated~~ caregivers will be…
    - A separate consent is not required from the ***designated*** parent~~s/designated~~ caregiver~~s~~; however, ~~all~~ ***the*** parent~~s/designated~~ caregivers will be given the Parent/Caregiver Information Sheet (Appendix I) to review prior to obtaining the study consent/assent.
    - Added the following sentence: ***Patients must be enrolled on or after day 1 of maintenance cycle/course 3.***
    - ~~Whenever an institution enrolls a new patient,~~ ***T***~~t~~he coordinating center (City of Hope) will request submission of a Study Participation Worksheet ***every 3 months***, which will include the following information: the number of patients eligible, the number of patients approached, and the number of patients who refused since the institution's last ~~enrollment~~ ***report***.
    - Parents/caregivers and patients will be informed that the major goals of the study are to institute mechanisms to improve adherence to daily oral 6MP; ***that all parents/caregivers and patients ≥ age 12 will watch an educational video; and*** that some of the patients will ~~watch an educational video,~~ receive a system of reminders for oral 6MP, and a ***designated*** parent/ ~~designated~~ caregiver will ~~observe~~ ***supervise*** the intake of the drug – while ~~the~~ others will continue to take their medications as per their usual routine***, in addition to having watched the educational video (i.e., all parents and patients ≥ age 12).***

- Revised the following in Section 4.1.1, paragraph 4 (page 16):

At the Day 1 appointment, the ~~site~~ CRA will assign a MEMS® *TrackCap™* to the patient and instruct the patient ***and parent/caregiver*** to start using the MEMS® *TrackCap™* when taking each prescribed dose of 6MP throughout the study period. The site CRA will follow-up by phone with the ~~patient~~ ***parent/caregiver*** on the next business day following enrollment…

- Section 4.1.1, revised paragraphs 5-7 (page 17), to define race/ethnicity and data needed on the On Study form:

On Day 1, the CRA will complete the Eligibility eRDE Form, and the eRDE system will automatically randomize participants to a study assignment (Intervention Program [IP] or Education Only [EDU]). CRAs will need to enroll the patient in the correct stratum, using the self-reported race/ethnicity of the patient (African-American, Asian, Caucasian, Hispanic, and Other) and age of the patient (< 12 years, ≥ 12 years). Definitions of the race/ethnicities are listed below:

African-American:
Includes patients who are African-American or of sub-Saharan black African ancestry
Asian:
Patients of Asian ancestry, including the following: Asian Indian (subcontinent), Chinese, Japanese, Korean, Native Hawaiian, Guamanian or Chamorro, Pacific Islander, Filipino, Vietnamese, Samoan, Hmong, Cambodian, Thai, Laotian, or Other Asian races
Caucasian:
Includes white or light-skinned patients of European, North African, or Middle Eastern ancestry
Hispanic:
Patients of Hispanic ethnicity, including the following: Mexican, Mexican American, Chicano, Cuban, Puerto Rican, or Other Spanish / Hispanic / Latino ethnicities
Other:
Patients of who do not self-report race/ethnicity in the categories listed above (multi-ethnic/multi-racial, Native American, etc.)

~~Participants should NOT be notified of their randomization until Day 29.~~ ***On the next business day following enrollment, after confirming use of the TrackCap, the CRA will complete the OnStudy eRDE form,*** ~~On Day 1, the CRA will complete the Eligibility eRDE form,~~ which will document ***the following:***

- ***The designated parent/caregiver who is willing to enter into a mutual agreement with the patient to participate in a daily supervised medication administration routine (e.g. "Mother", "Father," "Grandmother") as described in Section 4.1.5.2.3. For patients who travel between more than one household, only one designated parent/ caregiver (usually the parent/caregiver with whom the child spends the majority of his/her time) will be identified.***
- ***Whether cellular telephones will need to be assigned to the designated parent/caregiver and/or patient (if ≥ 12 years)***
- ***The language preference (English or Spanish) of the designated parent/caregiver and patient (if ≥ 12 years)***

~~parent/designated caregiver (i.e., adult physically present in the home) who is willing to enter into a mutual agreement with the patient to participate in a daily supervised medication administration routine (e.g. "Mother", "Father," "Grandmother") as described in Section 4.1.5.2.3. For patients who travel between more than one household, a parent or designated caregiver will be identified on the form for each household.~~

~~In addition, the CRA should determine if cellular telephones will need to be assigned to the parent/designated caregiver(s) and/or patient in the event that the patient is randomized to the IP arm, and the language preference (English or Spanish) of the parent/designated caregiver(s) and patient. The On Study form is available on the COG protocol webpage to assist CRAs with the collection of this information from the patient and parent/caregiver. On Day 1, the CRA will complete the Eligibility eRDE Form, and the eRDE system will automatically randomize participants to a study assignment (Intervention Program [IP] or Usual Care [UC]). Participants should NOT be notified of their randomization until Day 29.~~

Participants should NOT be notified of their randomization until Day 29…

Section 4.1.3 (page 18)

- Questionnaires will be completed by the designated parent/caregiver and by patients (if ≥ 12 years):

Questionnaires will be completed independently by the patient ***(if ≥ 12 years)*** **and** by ***the designated*** parent/caregiver (see Table ~~2~~ ***3*** for data collection schedule). ***The questionnaires will be completed only by the parents of the patients <12 years of age.*** ~~For patients who travel between more than one household, a questionnaire will be completed by one parent/caregiver from each household. Exception: Only one Demographic Questionnaire will be completed per enrolled patient, as outlined in Section 4.1.3.1.~~

Section 4.1.3.1 (page 18)

- The following changes were made to the demographics questionnaire description:

A brief Demographic Questionnaire will be completed by the ***designated*** parent/~~designated~~ caregiver (or patient if 18 years of age or older) on Day 1, and includes information related to self-reported race/ethnicity, socioeconomic status, and family structure. ~~If the patient is under age 18 and resides in more than one household, the Demographic Questionnaire will be completed by the parent/designated caregiver from the primary household.~~

Section 4.1.3.2 (page 18)

- The following sentences were revised:
  - ***The designated parent/caregiver and*** ~~P~~***p***atient ***(if ≥ 12 years)*** ~~and parents/caregivers~~ will be asked…
  - All items will be collected from participants on both study arms (IP and ~~UC~~**EDU**) at baseline (Day 1), at 4 weeks following implementation of the intervention (Day 57), and at completion of the study (Day 141) to allow comparison of the questionnaire data before and after ~~the IP group~~ ***all patients*** views ~~the Educational Program~~ ***MIPE*** and before and after ***the IP arm*** experienc***es***~~ing~~ the text messaging ***and DST*** portion of the intervention.
  - ~~Two separate Adherence Questionnaires, one each for the patient and parent/caregiver, will be available in both comprehensive and abbreviated versions.~~

Section 4.1.3.3 (page 18)

- The following changes were made:

  Intervention Rating Questionnaire ***(IP only)***
  On Day 141, patients ***(if ≥ 12 years)*** and parents/caregivers assigned to the IP arm will be asked to complete a brief Intervention Rating Questionnaire to rate their perceptions regarding the helpfulness of the MIPE program, cell***ular text message*** ~~phone~~ reminders (REM), and ~~DOT~~***DST***. Estimated completion time is 5 minutes. ~~See Section 4.1.5 for details of the study intervention.~~

Section 4.1.3.4 (page 19)

- Added section 4.1.3.4. Patients randomized to the EDU arm will watch the MIPE program and will be asked to complete a MIPE Rating Questionnaire at the end of the study:

  4.1.3.4 MIPE Rating Questionnaire (EDU only)
  On Day 141, patients (if ≥ 12 years) and parents/caregivers assigned to the EDU arm will be asked to complete a brief MIPE Rating Questionnaire to rate their perceptions regarding the helpfulness of the MIPE program. Estimated completion time is 5 minutes.

Section 4.1.3.5 (page 19)

- This section was previously mislabeled as section 4.1.3.3.4. The first sentence was modified:

  4.1.3.~~3.4~~**5**. Acculturation Questionnaire (Hispanic participants only)
  Hispanic patients ***(if ≥ 12 years)*** and their parents/caregivers…

Table 3 (previously ~~Table 2~~) (page 19)

- ~~UC~~ changed to **EDU**
- Added MIPE Rating Questionnaire
- Clarified that patients will only complete questionnaires if they are age 12 or older

Section 4.1.4 (page 19)

- Corrected spelling of Medication in section title
- The CRA will call the parent/caregiver to confirm use of the MEMS cap:

  The CRA will follow-up by phone with the ~~patient~~ *parent/caregiver* on the next business day…

Section 4.1.5 (pages 20 and 21)

- ~~Directly Observed Therapy (DOT)~~ replaced with ***Directly Supervised Therapy (DST)*** and added ***(if ≥12 years)*** for the patients in Table 4 (previously Table 3)
- Last paragraph revised:

  Upon enrollment, subjects are randomized 1:1 to receive ~~usual care~~ **Education Only** (~~UC~~***EDU***) or ***the*** Intervention Program (IP) (Figure 1). The first 28 days of the trial constitute an observational or "break-in" period – when all study participants (irrespective of the randomization arm) receive their ~~medications~~ ***6MP*** from…
- Figure 1 updated so that the ~~UC arm~~ is now the **EDU arm** and the EDU arm will also watch the MIPE program

Section 4.1.5.1 (page 21)

- The ~~UC arm~~ is now the **EDU arm** and will watch the MIPE program (EDU version):

  4.1.5.1 ~~Usual Care~~ *Education Only* (~~UC~~*EDU*)
  On Day 29, the designated parent/caregiver and patient (if ≥ 12 years) will view ~~an~~ multimedia interactive education program (MIPE – EDU version) during their scheduled clinic visit; the institutional CRA will submit documentation

certifying completion of this portion of the intervention (see Day 29 Documentation Form). There are two EDU versions of the MIPE program, depending on the age of the participant: < 12 years and ≥ 12 years.

The MIPE (EDU version) program features video clips of ALL patients of various ages (and their parents/caregivers) discussing maintenance treatment. Content is accessible in English and Spanish. Participants choose the patient/parent/caregiver they most closely identify with and view a variety of video clips featuring the selected patient/parent/caregiver addressing adherence-specific content, including the purpose and importance of taking oral 6MP, health beliefs related to perceived susceptibility/severity of illness, perceived benefits of and barriers to maintenance therapy, and examples of ways that patient/ parents have overcome barriers associated with taking oral 6MP to improve their own/their child's adherence. Video clips featuring explanations from healthcare professionals are also integrated into the program. The multimedia program provides the flexibility for participants to customize their learning experience based on their specific learning needs and linguistic preferences in a culturally-sensitive learning environment. The program, available in the clinic on-line or via DVD, takes 20 to 25 minutes to view, depending on the choices made by participants in their interaction with the program. This intervention is designed to address the clinical implications of the Health Belief and Self-Efficacy Models, in which individuals who understand the perceived benefit of a health behavior (i.e., taking oral 6MP as prescribed) are more likely to take control of that behavior (i.e., improve adherence).

<u>Section 4.1.5.1.1 (pages 21 and 22)</u>

- This section was added if the designated parent/caregiver is not present at the Day 29 appointment, when the MIPE program is viewed:

4.1.5.1.1 Alternative MIPE EDU Viewing for the Designated Parent/Caregiver
The designated parent/caregiver should be encouraged to be present at the Day 29 appointment. However, if they are unable to accompany the patient to the Day 29 appointment, the CRA will provide an Alternative MIPE EDU Viewing Packet to be sent home with the patient. The Alternative MIPE Viewing Packet will include the MIPE EDU program (< 12 years or ≥ 12 years version) and a letter with instructions for watching the MIPE EDU program within 24 hours of the patient's Day 29 clinic appointment. On the next business day following the Day 29 clinic visit, the CRA will call the designated parent/caregiver to confirm that the designated parent/caregiver has watched the MIPE EDU program, and will document this on the Day 29 Documentation Form. If the MIPE EDU program has not been viewed at the time that the CRA makes the follow-up call, the CRA will ask the parent/caregiver if they agree to view the program on that day. If the parent/caregiver agrees, the CRA will call back the following day to confirm that the program has been viewed and will document this on the Day 29 Documentation Form; if the MIPE EDU program has not been viewed at the time of the second follow-up call, the CRA will document this on the Day 29 Documentation Form.

<u>Section 4.1.5.2.1 (page 22)</u>

- The IP patients will watch the IP version of the MIPE program and patients will only watch the program if they are 12 years or older. The Intervention Documentation form is now called the Day 29 Documentation Form (will also be used for EDU patients to document MIPE viewing).

4.1.5.2.1 Multimedia Interactive Patient Education (MIPE – *IP version*)
On Day 29, ***the designated parent/caregiver and*** patients ***(if ≥ 12 years)*** ~~on the IP arm and their parent/designated caregiver~~ will view a~~n~~ ~~interactive~~ multimedia ***interactive*** education program ***(MIPE – IP version)*** during their scheduled clinic visit; the institutional CRA will submit documentation for each patient randomized to the IP arm to certify completion of this portion of the intervention (see ~~Intervention~~ ***Day 29*** Documentation Form). ***There are two IP versions of the MIPE program, depending on the age of the participant: < 12 years and ≥ 12 years. The content is identical to the MIPE EDU program, except the IP version also includes details about the customized instructions, cell phone reminders, and DST.*** ~~This interactive multimedia web application features video clips of ALL patients of various ages (and their parents/caregivers) discussing maintenance treatment. Content is accessible in English and Spanish. Participants choose the patient/parent/caregiver they most closely identify with and view a variety of video clips featuring the selected patient/parent/caregiver addressing adherence-specific content, including the purpose and importance of taking oral 6MP, health beliefs related to perceived susceptibility/severity of illness, perceived benefits of and barriers to maintenance therapy, and examples of ways that patient/ parents have overcome barriers associated with taking oral 6MP to improve their own/their child's adherence. Video clips featuring explanations from healthcare professionals are also integrated into the program. The multimedia program provides the flexibility for participants to customize their learning experience based on their specific learning needs and linguistic preferences in a culturally-sensitive learning environment. The program,~~

~~available in the clinic on-line or via DVD, takes 10 to 15 minutes to view, depending on the choices made by participants in their interaction with the program. This intervention is designed to address the clinical implications of the Health Belief and Self-Efficacy Models, in which individuals who understand the perceived benefit of a health behavior (i.e., taking oral 6MP as prescribed) are more likely to take control of that behavior (i.e., improve adherence).~~

Section 4.1.5.2.2 (page 22)

- Changed ~~MEDACTIONPLAN ALL Maintenance Therapy Template~~ to ***ACCL1033 MEDACTIONPLAN ALL 6MP Schedule Template***
- Added the third sentence to describe the ACCL1033 study specific MedActionPlan website:

A customized MEDACTIONPLAN portal was created specifically for the ACCL1033 study (https://www.medactionplan.com/ct/network.asp).

- The last sentence of this section was updated with intervention instructions for the healthcare provider and parent/caregiver:

Standardized instructions regarding the REM portion of the intervention will be provided to the CRAs for dissemination to the healthcare providers caring for patients randomized to the IP arm prior to Day 29 ***(Intervention Instructions for the Healthcare Provider)*** and ~~to patients and their~~ parents/caregivers on Day 29 ***(Intervention Instructions for the Designated Parent/Caregiver). On Day 29, the CRA will review the instructions with the parent/caregiver and patients (≥ 12 years).***

Section 4.1.5.2.3 (page 23)

- If patients reside in multiple households, only one parent/caregiver will be designated for the study. If the patient isn't with the designated parent/caregiver at the time the 6MP dose is due, the designated parent/caregiver will call the patient to confirm that the dose was taken. The DST instructions have also been clarified.

4.1.5.2.3 Directly ~~Observed~~*Supervised* Therapy (~~DOT~~*DST*)

On Day 29, the designated parent/ caregiver who enters into a mutually agreed upon partnership with the patient, is trained briefly in prompting and ~~observing~~ supervising the patient take his/ her medication on a daily basis. ~~For patients who travel between more than one household, a designated caregiver will be identified at each household, and each designated caregiver will be required to undergo training on Day 29 (see Section 4.1.5.2.4 for Alternative Training if designated caregiver(s) is/are not at the Day 29 clinic visit).~~ Once the designated parent/caregiver receives the text-message, they are instructed to prompt initiation of the dose (if necessary), and to ~~visually~~ supervise the ingestion of the prescribed 6MP (in all instances). ***If the patient is not present when the dose of 6MP is due (patients who travel between two households, away at college, staying with a relative, etc.), the designated parent/caregiver will call the patient (or other parent/caregiver for patients < 12 years) to prompt initiation of the dose.*** After the patient has taken the scheduled dose, ***the designated parent/caregiver*** ~~s/he~~ ***and patient (if ≥ 12 years)*** ~~and the designated caregiver~~ respond to the text message with a standardized reply function on the cell phone, providing a real-time central record of adherence. This training for ~~DOT~~***DST*** is a component of the MIPE program ***(IP version only)*** that is viewed on Day 29 ***by parents/caregivers and patients (if ≥ 12 years)***; additionally, standardized instructions will be provided to ~~patients and their~~ parents/caregivers regarding the ~~DOT~~***DST*** portion of the intervention on Day 29 ***(Intervention Instructions for the Designated Parent/Caregiver). On Day 29, the CRA will review the instructions with the parent/caregiver and patients (≥ 12 years).*** Parents/caregivers and patients ***(if ≥ 12 years)*** who do not have a cell phone, or who have limited or no text-messaging capability on their current cell phone, will be issued a ***study*** cell phone with unlimited ***phone and*** text messaging capabilities for use for the 4-month duration of the intervention. For patients ~~under~~ age***d 12-17 years*** ~~18~~, parental permission … ***CRAs will indicate on the OnStudy eRDE form*** ~~Please contact the coordinating center (City of Hope 626-256-HOPE (4673) ext. 61189; AdherenceStudy@coh.org)~~ if a cell phone needs to be assigned to the parent/caregiver and/or patient***, and the Coordinating Center (City of Hope), will mail the phone(s) to the CRA.***

Section 4.1.5.2.4 (page 23)

- If a patient resides in multiple households, only one parent/caregiver will be designated for the study. Other minor clarifications were also made to this section:

4.1.5.2.4 Alternative Intervention Training for the Designated Parent/Caregiver

The designated parent/caregiver~~(s)~~ should be ***strongly*** encouraged to be present at the Day 29 appointment. However, if they are unable to accompany the patient to the Day 29 appointment, the CRA will provide an Alternative Intervention Training Packet to be sent home with the patient. The Alternative Intervention Training Packet will include the MIPE ***IP*** program ***(< 12 years or ≥ 12 years version)***, ~~standardized~~ ***detailed*** instructions for the ~~DOT~~***DST*** and REM portions of the intervention, and a letter with instructions for completing the intervention training within 24 hours of the patient's Day 29 clinic appointment. ~~When the CRA calls to confirm the use of the MEMS® TrackCap™ on the next business day following the Day 29 clinic visit (see Section 4.1.1), they~~ ***The CRA will call the designated parent/caregiver the following business day to review the instructions and*** ~~will also~~ confirm that the designated parent/caregiver has completed the Alternative Intervention Training. ***The CRA*** ~~and~~ will document this on the ~~Intervention~~ ***Day 29*** Documentation Form. If the Alternative Intervention Training has not been completed at the time that the CRA makes the follow-up call, the CRA will ask the parent/caregiver if they agree to complete the training on that day. If the parent/caregiver agrees, the CRA will call back the following day to confirm that the training has been completed and will document this on the ~~Intervention~~ ***Day 29*** Documentation Form; if the training has not been completed at the time of the second follow-up call, the patient will be removed from the study and the CRA will complete the Off Study form.

~~If the patient will be residing at additional household(s) during the study period and the designated parent/caregiver at the alternative household(s) was not present for the Day 29 training, the CRA will supply the patient with an additional Alternative Intervention Training Packet for each additional household; training for parent/caregiver(s) at the additional household(s) is to be completed on the first day that the patient arrives at that additional household(s). The CRA will document on the Intervention Documentation Form that these instructions have been conveyed to the family.~~

Section 4.1.5.2.5 (page 23 and 24)

- The Intervention Documentation Form is now called the Day 29 Documentation Form and CRAs will document that the MIPE program was viewed on Day 29 (all patients) and for patients randomized to the intervention, that the intervention training was completed:

4.1.5.2.5 *Day 29* Documentation ~~of Intervention~~ *Form*

On Day 29, the institutional CRA will complete the ~~Intervention~~ ***Day 29*** Documentation Form, which includes documentation of (1) completion of MIPE viewing by the patient ***(if ≥ 12 years)*** and ***designated*** parent/caregiver ***(IP and EDU arms)*** and (2) provision of standardized instructions regarding the REM and ~~DOT~~***DST*** portions of the intervention to the healthcare provider, patient ***(if ≥ 12 years)*** and parent/caregiver ***(IP arm only)***. If the designated parent/caregiver is not present at the Day 29 appointment, the CRA will document that an Alternative Intervention Training Packet ***(IP arm only) or Alternative MIPE EDU Viewing Packet (EDU only)*** was sent home with the patient and a follow-up phone call was made on the ~~first business day following study enrollment~~ ***following business day*** to confirm completion of training. The CRA will submit the completed ~~Intervention~~ ***Day 29*** Documentation Form within 1 week of the Day 29 clinic visit to Dr. Smita Bhatia at the study coordinating center (City of Hope) via ***email,*** mail***,*** or fax (contact information below) ~~or via *EMAIL:* AdherenceStudy@coh.org~~.

Section 4.2 (page 24)

- A Day 141 Lab form has been added to record CBC and LFT values from Day 141:

  For each patient registered on ACCL1033, a Disease History and Therapeutic Summary, ~~and~~ five Maintenance Report Worksheets***, and a Day 141 Lab form,*** will be…
- Added the following to the second to last sentence in the first paragraph: ***, measurement of 6TGN levels***
- Revised the last 3 sentences of the second paragraph:

  ~~Maintenance Report Worksheets may be completed and submitted together in batches, to correspond with therapeutic protocol-specified reporting periods.~~ Please ***e***mail or fax **within 4 weeks** of completion of each ~~therapeutic protocol-specified reporting *period*~~ ***timepoint*** ~~Please mail completed worksheets~~ to the coordinating ***center*** ~~institution at the address below~~:

Table 5 (previously Table 4) (page 25)

- ~~UC~~ changed to **EDU**
- EDU arm will view MIPE and complete MIPE Rating Questionnaire

Added Section 4.5: (page 26)

4.5 Follow-Up Data Collection

Study participants will be followed closely at their respective treating institutions for the following events: i) death, ii) relapse and iii) second malignancy. For patients that relapse or develop a second malignancy, institutions will need to submit documentation of the event (clinic note, pathology report, etc.). The Coordinating Center will contact participating institutions to obtain an update on the vital and disease status of the study participants. A follow-up form will be requested every 6 months for 5 years from the time of completion of the study, and then annually until 10 years from diagnosis.

Section 5

Several changes were made to the Statistical Considerations section since the study design changed. The Usual Care arm is now the EDU arm and will also watch the MIPE program on Day 29. The EDU arm will be compared to the IP arm, as well as a historical control (HC). The target accrual is now 570 patients; younger patients and patients of all race/ethnicities will now be included in the study. All changes are shown below:

5.1 Statistical Design (page 26)

This is a randomized clinical trial. ***This trial determines the impact of interventions proposed in IP vs. that proposed in EDU on adherence to oral 6MP in children with ALL; it also determines the impact of the education program on adherence to 6MP by comparing patients on EDU to an un-intervened historical control (HC) group drawn from the COG AALL03N1 study. In general, we anticipate the effect of Edu to be intermediate between that of IP and HC.*** ~~The study will test the hypothesis that use of an intervention that comprehensively addresses barriers to adherence, and consists of a Multimedia-based Interactive Patient Education program (MIPE) and a web-based system for creating customized 6MP schedules and automated electronic reminders (REM) in the patients' preferred language, in the setting of directly observed therapy (DOT), will significantly enhance adherence to oral 6MP.~~ At the time of enrollment, participants will be automatically randomized via the eRDE system. Eligible participants will be randomized to a study assignment (IP or ~~UC~~***EDU***) using a blocked stratified randomization with ethnic background (~~3~~ ***5*** strata) ***and age at the time of randomization (2 strata)*** as stratification factors and a block size of 6 to balance the number of participants in each arm. ~~A total of 340 evaluable subjects are needed to address the aims of the study: 170 each in IP and UC arms; and within each arm, 68 each from non-Hispanic white and Hispanic backgrounds and 34 from African-Americans.~~

Table 6. ACCL1033 Patient Accrual

	<12 years at study participation	***≥12 years at study participation***	***Total number needed for trial***	***Total number to be enrolled to account for attrition***
Non-Hispanic whites	***80***	***58***	***138***	***170***
Hispanics	***80***	***58***	***138***	***170***
African American	***34***	***24***	***58***	***75***
Asians	***34***	***24***	***58***	***75***
Other	***38***	***26***	***64***	***80***
Total	***266***	***190***	***456***	***570***
See Section 3.1.1 for the race/ethnicity definitions				

5.2 Accrual (and Expected Duration of Accrual) (page 27)

We plan to enroll a maximum of ~~400~~ ***570*** subjects, ~~200~~ ***285*** each in IP and ~~UC~~***EDU*** arms, and within each arm ~~75 each from non-Hispanic white and Hispanic backgrounds and 50 from African-Americans~~ ***the numbers to be enrolled are shown by race/ ethnicity and age in Table 6***, such that, after accounting for attrition due to study withdrawal there will be ~~170~~ ***228*** evaluable patients in the IP arm and ~~170~~ ***228*** patients in the ~~UC~~***EDU*** arm, for a total of ~~340~~ ***456*** evaluable patients. The expected duration of accrual is 48 months. Based on the 136 institutions that have participated in AALL03N1, these institutions diagnose 1,784 children with ALL each year (non-Hispanic whites: n=1,123; Hispanics: n=381, African-Americans: n=118***, Asians: n=77; Others: n=45***). Thus, over the 48-month accrual period, these sites will see approximately 4,500 newly diagnosed non-Hispanic whites, 1,525 Hispanics, ~~and~~ 475 African-Americans, ~~and~~ ***300 Asians, and 180 Others*** with ALL; ***providing an ample sized pool for recruitment.*** ~~based on prior accruals, we project that approximately 22% will be over age 10 at diagnosis (and therefore over age 12 at time of enrollment). We thus anticipate that approximately 1,000 non-Hispanic white, 350 Hispanic patients, and 105~~

~~African-American children will meet eligibility criteria over the 48 month accrual period, providing ample patients available for recruitment.~~

5.3 Analytic Plan (pages 26 and 28)

We will treat ~~adherence to therapy measured by RBC 6TGN levels as a continuous variable,~~ adherence measured by MEMS as ~~both~~ a binomial ~~and a binary~~ outcome ***in each patient, which will then be used to classify him/her as adherent or not (binary outcome),*** ~~, and self-report as a binomial variable.~~ The binomial variable~~s are~~ ***is*** expressed as proportion, rate, or (by multiplying by 100) percent adherence. ~~Measurements will be obtained or calculated at 5 time points (Table 5), each occasion constituting a 28-day period (t1, t2, t3, t4, and t5, for days 1-28, 29-56, 57-84, 85-112, and 113-140, respectively). Intervention begins after t1 on Day 29.~~

~~Table 5: Time periods for data collection and adherence assessment by study day~~

	~~Observation only~~	~~Adherence Intervention* vs. Usual Care~~								
~~Study Days~~	~~Days 1-28~~	~~Day 29~~	~~Days 29-56~~	~~Day 57~~	~~Days 57-84~~	~~Day 85~~	~~Days 85-112~~	~~Day 113~~	~~Days 113-140~~	~~Day 141**~~
~~Study activity~~	~~Break-in period~~	~~Intervention vs. usual care~~								
~~Study time point~~	~~T1~~		~~T2~~		~~T3~~		~~T4~~		~~T5~~	
~~Adherence assessment~~		~~Baseline~~		~~Month 1~~		~~Month 2~~		~~Month 3~~		~~Month 4~~

~~* The intervention begins on Day 29 and ends on Day 140~~
~~**Day 141 is the end of study visit, which is the day that the final blood is drawn, the final questionnaires are completed, and the MEMS® TrackCap™ and cell phones are collected.~~

Our analysis plans ***are*** described below.

~~Aim~~ ***Primary Aim*** ~~1~~: ***Determine the impact of interventions proposed in IP vs. EDU on adherence to oral 6MP in children with ALL. Adherence will be measured by: i) MEMS (primary measure of adherence to oral 6MP, providing real-time data; ii) red cell TGN levels (providing data on chronic, systemic 6MP exposure)*** ~~Determine the impact of the intervention program on adherence to 6MP using the following adherence assessments: *(1) Frequency of 6MP dosing using an electronic pill monitoring system (**MEMS**) (Primary measure of adherence); (2) RBC 6MP **6TGN levels** (dosage- and TPMT-normalized); and (3) Self-report of adherence*~~

The primary outcome of this trial is the ***proportion of patients with*** adherence rate ***≥95%*** to 6MP ***over the 4 month study period. Adherence rate is*** defined as the ratio of MEMS pill container openings to the number of days that 6MP doses were prescribed during ***the 4*** ~~each study~~ month~~s~~ ***study period***. It is also referred to as percent adherence by multiplying the rate by 100. ***Patients whose adherence rates are ≥95 are designated as "adherent." Because of*** ~~Since participants are~~ randomi***zation***~~zed~~, we ***expect the participants in each group (IP and EDU) to be similar*** ~~do not expect the adherence rate to differ by treatment status~~ at baseline. ~~Nevertheless,~~ ***W***~~W~~e will ***verify this*** ~~evaluate the success of randomization~~ by comparing group differences in demographic and clinical characteristics (i.e. socioeconomic status, gender, age at enrollment, and number of months on maintenance prior to enrollment) and adherence measures at baseline. If imbalance is evident, analysis ***(described below)*** will take into account the covariates with significant imbalance.

Using an intention-to-treat analysis, the effectiveness of the Intervention Program in improving ***the proportion of*** adheren***t***~~ce~~ ***patients*** will be determined by comparing the ***proportion of adherent*** ~~primary outcome, i.e. the adherence rate measured by MEMS® *TrackCap*™~~ ***patients*** ~~between patients~~ in the IP and ~~UC~~***EDU*** group***s using logistic regression***~~, applying the Generalized Estimation Equation (GEE) method for longitudinal binomial outcomes~~. Adheren***t patients will be designated as 1 and non-adherent patients as 0. An intervention group indicator (1 for IP, 0 for EDU) will be included in logistic regression and its significance tested.*** ~~ce rate will be modeled as linear function of time (shown as sufficient in our ALL data), with an interaction of time by treatment group indicator (0 for UC, 1 for IP) to allow for treatment differences. A significant group by time interaction, with a larger positive slope for the IP group compared to UC, will indicate effectiveness of the IP in improving adherence. We will use the compound symmetry covariance structure which has been shown to fit the data well.~~ Since this ***test will be conducted for <12 and ≥ 12 years old (Hypothesis 1.2) as well as with all ages combined (Hypothesis 1.1) for a total of 3 tests, we*** will ~~be the test of our primary hypothesis,~~ ***use*** a ***2-sided*** Type I error rate of 0.0***175*** ~~5~~ ***for each test*** ~~and 2-sided test~~***, for***

an overall Type I error rate of 0.05. ~~will be used. If the linear model is significant, we will test for non-linear trends. This will be done by including an interaction of the group by quadratic time interaction. We will also explore non-linearity by fitting an unstructured mean model, using 4 indicator variables representing 5 time points. Interactions of time with treatment group will be included to allow for treatment difference from t2 to t5 and their significance tested using a 4-df chi-square test.~~

~~We have identified < 95% as a clinically relevant adherence rate associated with increased risk of relapse (Bhatia et al, JCO, in press). Therefore, to determine if the intervention reduces the percent of participants who are nonadherent, we will conduct a secondary analysis by dichotomizing percent adherence based on the MEMS data, classifying an individual as adherent (1) if the MEMS pill container was opened 95% or more of the time in which 6MP was prescribed in a study month and non-adherent (0) otherwise. The binary outcome will be analyzed using the GEE method for longitudinal binary outcome.~~ We will also model the level of 6TGN as a function of time to examine longitudinal changes in 6TGN levels between the treatment groups using the GEE method for longitudinal normally distributed data. ~~Finally, the self-reported number of doses missed out of the prescribed days in each 28-day study period will be treated as a binomial variable and modeled longitudinally using the GEE methods to determine if self-reported longitudinal adherence rate differs between the treatment groups.~~

Secondary Aim 1: Determine the impact of the education program on adherence to 6MP by comparing patients on EDU to an un-intervened historical control (HC) group drawn from the COG AALL03N1 study

To determine whether EDU increases the proportion of adherent patients compared to HC (Hypothesis 2), we will first calculate the adherence rates over the 4 month study period in EDU patients and in HC patients using data from the initial 4 months. Each patient will then be classified as adherent or not. Logistic regression with a group indicator (1 for EDU and 0 for HC), adjusted for sociodemographic and clinical variables, will be used to test if the proportion of adherent patients in EDU is higher than that in HC. A 2-sided Type I error of .05 will be used. In this analysis, patients of "Other" race will be excluded from EDU since they are not part of HC.

Secondary Aim 2 ~~**Aim 2**~~***: Examine the modifying effect of sociodemographic characteristics, health beliefs/ knowledge, and degree of acculturation on changes in adherence with the IP (IP vs. EDU; and EDU vs. HC)***.~~Examine the modifying effect of **sociodemographic** and **clinical** characteristics and **health beliefs/ knowledge** on changes in adherence with the intervention program.~~

Sociodemographic data (ethnicity, parental education, annual household income, family structure, household living conditions) will be obtained from the ~~parent-completed~~ Demographic Questionnaire. Clinical characteristics will be obtained from the data capture forms completed by the institutional clinical research assistants and variables relevant to health beliefs/knowledge will be obtained from the patient and parent/caregiver Adherence Questionnaires***, and degree of acculturation will be determined from the Acculturation Questionnaire***. The relationship between ***the proportion of adherent patients*** ~~changes in adherence by~~ ***and intervention*** ~~treatment~~ group ***(IP vs. EDU and EDU vs. HC)*** will be evaluated by lo***gistic regression*** ~~ngitudinal methods~~ as described above. The interactions of ***group*** ~~treatment~~ by ~~time by~~ modifying covariate*s* (categorical or continuous) will be included in the ***logistic regression*** model ~~for adherence rate per MEMS data~~ and their significance evaluated. ***We hypothesize that intervention effects will be significantly modified by age at study participation, race/ethnicity, and number of adult caregivers in household as well as perceived severity of illness, knowledge regarding purpose of 6MP, and perception of self-efficacy to overcome barriers.*** The relationship between ***the proportion of*** ~~changes in~~ adheren*t*~~ce~~ ***patients*** by treatment group, stratified by race/ethnicity, sociodemographic and clinical characteristics, ~~and~~ health beliefs/knowledge-related factors***, and degree of acculturation*** will also be evaluated analytically by lo***gistic*** ~~ngitudinal~~ regression methods. Since power for examining interaction effects are expected to be low, these analyses will be considered exploratory.

5.4 Power and Sample Size (page 28)

The primary outcome of this trial is the proportion of patients (P) who are ≥ 95% adherent at taking the oral 6MP over the 4 month intervention period as measured by MEMS. The effectiveness of the intervention in IP over that in EDU will be determined by comparing (over the study period) P_{IP} ***(the proportion of patients with adherence rate ≥95% in IP) to*** P_{Edu} ***(the corresponding rate in EDU), by testing Ho:*** $P_{IP} = P_{Edu}$ ***in age <12 years and age ≥ 12 years, as well as with all ages combined. To account for the 3 independent tests, a 2-sided Bonferroni-adjusted Type I error=0.017 (for an overall Type I error=0.05) will be used for each test. Under Hypothesis 1.2, for age < 12 years, 133 patients per arm will provide 80% power to detect a difference between*** P_{IP}***=0.90 and*** P_{Edu}***=0.75 (i.e., 90% of patients in IP vs. 75% of patients in Edu have adherence rates ≥95%), or an Odds Ratio (OR) of 3.0. For age ≥12 years, 95 patients per arm will provide 80% power to detect a difference between*** P_{IP}***=0.85 and*** P_{Edu}***=0.65 (i.e., 85% of patients in IP vs. 65% of patients in Edu have adherence rates ≥95%), or an OR=3.0. When samples from both age groups are combined (for Hypothesis 1.1), the total sample size of 456 (228 per arm) has 98% power to detect a difference between*** P_{IP}***=.0.88 and*** P_{Edu}***=0.71 (i.e., 88% of patients of all ages in IP have adherence rates ≥95% vs. 71% of patients of all ages in Edu), or an OR=3.0. An overall OR=1.85 could be detected at 80% power at this sample size.***

For Hypothesis 2 (Secondary Aim) in which EDU is compared HC, we cannot evaluate patients of other race since they are not available in HC. HC comprises 373 patients aged <12 years in whom 60% have adherence rate ≥95%, and 89 patients aged ≥ 12 years in whom 42% have adherence rate ≥ 95%; 56% of patients of all ages have adherence rate ≥95%. Two-sided Bonferroni-adjusted Type I error=.017 is also used for each of 3 tests. For comparing Edu with HC for age <12 years, 116 patients in Edu will provide 73% power to detect a difference between P_{Edu}=0.75 and P_{HC}=0.60 (i.e., 75% of patients in Edu have adherence rate ≥95% vs. 60% of the patients in HC), or an OR=2.0. For patients ≥12 years, 83 patients in Edu will provide 76% power to detect a difference between P_{Edu}=0.65 and P_{HC}=0.42 (i.e., 65% of patients in Edu have adherence rates ≥95% vs. 42% in HC), or an OR=2.6. When all ages are combined, 199 patients in Edu will provide 88% power to detect a difference between P_{Edu}=0.71 vs. P_{HC}=0.56, or an OR=1.9.

~~The primary endpoint of this trial is the adherence rate, defined as the ratio of MEMS pill container openings to number of days 6MP doses were prescribed in a study month. The effectiveness of the Intervention Program will be determined by comparing the adherence rate in the IP (P_{IP}) and UC (P_{UC}) groups over the study months. Because of randomization and since intervention begins 1 month after randomization, P_{IP} and P_{UC} at baseline (Day 29) are expected not to differ. Based on the COG AALL03N1 data (Section 2.4), among those ≥ 12 years at study participation, the mean adherence rate at month 1 is 0.94 (sd=.10) in non-Hispanic whites, 0.91 (sd=0.14) in Hispanics, and 0.80 (sd=0.22) in African-Americans. The corresponding means at month 5 are 0.92 (sd=0.16), 0.82 (sd=0.26), and 0.63 (sd=0.42) for the respective race/ethnicity groups. The expected weighted mean proportional adherence is 0.90 at the baseline in both treatment groups. At the end of the 5 month study period, P_{UC} (controls) is expected to have decreased to 0.82 (weighted average of 0.92, 0.82, and 0.63) with expected sd=0.15. At Type I error=0.05 and 2-sided alternatives, assuming a linear trend, intra-person correlation of 0.5, compound symmetry covariance structure, and an attrition rate of 25% (estimated from the COG AALL03N1 data for non-Hispanic white, Hispanic, and African-American participants age 12 or older), 170 patients per arm will provide 80% power to detect an effect size (treatment difference/common sd) of at least 0.32 at month 5, i.e. a difference between P_{IP} = 0.87 and P_{UC} = 0.82 (difference of +0.05 between the IP and UC groups at the end of the intervention period, common sd=.15 assumed). This calculation was performed using RMASS2.[38] Power for examining interaction effects (Aim 2) will be low (< 50%); therefore, analysis of the modifiers of intervention effects will be conducted as an exploratory analysis.~~

<u>Section 5.5 (page 29)</u>
The gender and minority accrual estimates were updated with the new accrual estimates.

APPENDIX I: YOUTH AND PARENT INFORMATION SHEET CHANGES (PAGE 30)

- The Youth Information Sheet (for children who are 12 years of age) is now for children aged 7-11 years. The following revisions were made:
    - Revised sub-title: (for children who are ~~12~~ ***7 to 11*** years of age)
    - Section 3 Changes:
        - 6th sentence: ~~You and y~~our parent will also be asked to answer some questions about your family and about how you are doing with taking your 6MP.
        - 7th sentence: Y~~ou and y~~***our parent will also watch a video on the Internet or on a DVD during one of your clinic visits. In the video, people talk about taking 6MP.***
        - Deleted the 9th and 10th sentences: ~~These children and their parents will watch a video on the Internet or on a DVD during one of their clinic visits. In the video, people talk about taking 6MP.~~
        - Revised 11th sentence: ~~These children and their~~ ***P***arents ***of these children*** will also receive text messages on their cell phones reminding them to ***give***~~take~~ 6MP each time it is due, and the parent will ~~watch~~ ***make sure that*** the child take***s*** each dose of 6MP during the study.
        - 12th sentence: deleted …~~children and~~ parents do not have cell phones...
        - 14th sentence: You have the same chance of being asked to take part in the ~~6MP video and~~ cell phone ***text messaging*** reminder program or not.

- Revised 1st sentence of section4:
  As part of this study, you will have some blood taken ***every month for a total of*** ~~at~~ 6 times.

- The Youth Information Sheet (for teens who are 13 through 17 years of age) is now for children/teens aged 12-17 years. The following revisions were made (page 32):
  - Revised sub-title: (for teens from ~~13~~ ***12*** through 17 years of age)
  - Section 3 Changes:
    - Added sentence #5: ***You and your parent will also be asked to watch an interactive video program on the Internet or on a DVD during one of their clinic visits. In the interactive video, people talk about taking 6MP.***
    - Deleted sentence #7: ~~These teens and their parents will be asked to watch an interactive video program on the Internet or on a DVD during one of their clinic visits. In the interactive video, people talk about taking 6MP.~~
    - Revised last part of sentence #8: … and the parent will ~~watch~~ ***make sure that*** the teen take***s*** each dose of 6MP during the study.
    - Revised last part of sentence #11: … take part in the ~~video and~~ cell phone reminder program.
    - Revised last sentence: That means there is also a 1 in 2 (50-50) chance that you will not be asked to take part in the ~~video and~~ cell phone ***text messaging*** reminder program.

- The following changes were made to the Parent/Caregiver Information Sheet (pages 34 and 35)
  - Revised 1st sentence of section 3: You <u>and</u> your child ***(if ≥ 12 years old)*** will be asked to complete ~~3~~ ***8*** to ~~5~~ ***10*** questionnaires over the course of the study.
  - Added section 4 and revised numbering of subsequent sections:
    4. You and your child (if > 12 years old) will view an educational video (available in English and Spanish) on the Internet or on a DVD during one of the child's clinic visits. In the video, patients and their parents talk about their experiences with 6MP.
  - Revised Intervention Program (Arm IP) Section (now #5)
    - Deleted 2nd Sentence: ~~These children and their parents will watch a video (available in English and Spanish) on the Internet or on a DVD during one of the child's clinic visits. In the video, children and teens and their parents talk about their experiences with 6MP.~~
    - Revised 3rd sentence: Children and teens taking part in the intervention program will ~~also~~ receive ~~a customized medication schedule at each monthly clinic visit and~~ text messages sent to their cell phone ***(if the child is ≥12 years old)*** and…
    - Revised 4th and 5th sentences: The parent will ~~watch~~ ***make sure that*** the child take***s*** each dose of 6MP during the study. If ***the*** child (***if***~~ren~~ ***≥12 years old)*** and/***or their*** parent~~s~~ do***es*** not have ***a*** cell phone~~s~~, or…
    - Revised 7th sentence: Parents will receive a short training session about how to prompt and ~~watch~~ ***supervise*** their child ***to*** take their 6MP dose…
    - The following was added in the last two sentences: ***(if ≥ 12 years old)***
  - Revised Education Only (Arm EDU) section (now #6)
    ~~**Usual Care**~~ ***Education Only*** **(Arm *EDU*~~UC~~)**: During this research study, some children will have their clinic visits as usual. Your child has the same chance of being asked to

take part in the ~~6MP video and~~ cell phone ***text messaging*** reminder program or not. You, your child, and your child's doctor will not decide this.

- Revised 4th sentence of section 7: At the second clinic visit since entering the study, a blood test (TPMT phenotyping) will be done to test for ~~certain types of genetic changes that relate to~~ how your child's body handles 6MP…

APPENDIX II: DATA SUBMISSION REQUIREMENTS FOR ACCL1033 CHANGES (PAGE 37)

- The data submission requirements table was updated to reflect the changes in the amendment. The Day 141 lab form and MIPE rating questionnaire were added to the table. Other changes were minor and included specifying the ages of the patients completing the questionnaire and revising when the forms are due.

INFORMED CONSENT CHANGES

<u>Section: Why is this study being done? (page 40)</u>

- Last part of first sentence: … with ~~usual care~~ ***the effects of an educational program***.
- Second sentence: ~~usual care~~ changed to ***only education***.
- Third sentence: In this study, you will get either the multi-component intervention or you will ~~receive care as usual~~ ***be asked to watch an educational video***.

<u>Section: What will happen on this study that is research? (page 41)</u>

- The descriptions of the study arms were revised:

<u>Arm IP</u>: Children/teens will receive multimedia interactive patient education ***(parents and patients ≥age 12 years)***, a customized medication schedule, medication reminders (cell phone text messages – ***parents and patients ≥ age 12 years***) and parent/caregiver training to supervise the child or teen's taking of medication ***in addition to usual care (whatever is normally done at their clinic to help people with taking their medications as directed)***

<u>Arm ~~UC~~***EDU***</u>: Children/teens will receive ***multimedia interactive patient education (parents and patients ≥ age 12 years) in addition to*** usual care (whatever is normally done at their clinic to help people with taking their medications as directed)

Some children and teens will be randomized to receive the education and reminder intervention (IP); others will be randomized to receive ~~usual care~~ ***education only*** (~~UC~~***EDU***).

<u>Study Diagram (page 42)</u>

- The study diagram was updated.

<u>Study activities for all participants section (page 42)</u>

- This section as revised accordingly: At various times during this study, you will be asked to complete ~~3~~ ***8 to*** ~~5-~~***10*** ~~different~~ questionnaires (how many depends on your ethnicity and which study arm you are on). We would also like to collect blood samples ***every month for a total of 6 samples*** ~~on 6 days~~ during the study.

<u>Study activities for participants on Arm IP section (page 43):</u>

- Added ***(if 12 years of age or older)*** after "You" throughout section
- In "Clinic Visit #3" section, changed:
- The program will take about ~~10~~***20***~~-15~~ ***25*** minutes to watch

- Deleted last sentence of 3rd paragraph: ~~If you live at more than one house, we will ask for a parent/caregiver at each house to supervise your medication-taking while at their house.~~
- In "Clinic Visits #4 through #6" section, revised the second sentence: You ***(if 12 years of age or older) and/or your parent/caregiver*** will continue…
- In "Clinic Visit #7" section, added the following: … you ***and/or your parent*** were given to use during the study.

Study activities for participants on Arm ~~UC~~***EDU***," (page 43)

- ~~UC~~ was changed to **EDU**. Other changes include:

Added "Clinic Visit # 3" section (page 43):

> ***Clinic visit #3***
> ***You (if 12 years of age or older) and your parent/caregiver will be asked to watch an interactive program (DVD or on the Internet) during your regularly scheduled clinic visit. The program will take about 20-25 minutes to watch, and can be viewed in English or Spanish. The program discusses what Maintenance therapy is and why it is important, the reasons for taking 6MP, problems people face in taking medications as directed, and provides video clips of patients and parents talking about their experiences with taking 6MP. If your parent/caregiver cannot be at this clinic visit, a packet will be sent home with you that contains the DVD.***

- Changed the next section title: Clinic visit #~~3~~ ***4*** through #7 (page 44)

Under the "Blood tests for TPMT phenotyping and 6MP levels" section, (page 44, in "Clinic Visit #2") ,

- The following was deleted: to test for ~~a certain type of genetic change that relates to~~ how your body handles 6MP (TPMT phenotyping) (page 44)

Questionnaire descriptions (pages 44 and 45):

- In the "Full Medication Questionnaire", "Short Medication Questionnaire", and "Additional Questionnaire for participants who are Hispanic" sections, "***(if 12 years of age or older)"*** was added since only patients ages 12 years and older will be completing these questionnaires:
- Added the Education Program Questionnaire for patients on the EDU arm:

<u>Additional Questionnaire for participants on the EDU arm ONLY</u>:
We will also ask you (if 12 years of age or older) and your parent/caregiver to complete the following at clinic visit #7 for the study:
<u>Education Program Questionnaire</u>: We will ask questions about the education program and how helpful you felt it was. This questionnaire will take about 5 minutes to complete.

- In the "<u>Additional Questionnaire for participants on the IP arm ONLY</u>" section, the following changes were made:
- The first sentence was revised: We will also ask you ***(if 12 years of age or older)*** and your parent/caregiver to complete the following ***at clinic visit #7 for the study***:
- Deleted the last sentence: ~~We will ask you to complete this questionnaire at your last clinic visit for this study (visit #7).~~

<u>Section: What side effects or risks can I expect from being in the study? (page 45)</u>

- Added the following risk:

<u>Additional Risks for participants on the EDU arm</u>:
In addition to the risks above, the risks associated with viewing the educational video are considered minor, and may include discomfort in taking part in this activity.

- Added "***and***" in the third paragraph

<u>Section: Are there benefits to taking part in the study? (page 46)</u>

- The following was added to the second paragraph: While study doctors hope the ***educational video and***…

<u>Section: How many people will take part in the study? (page 46)</u>

- Increased total number of persons enrolled from ~~400~~ to ***570***

<u>Section: How long is the study? (page 46)</u>

- Follow-up forms will now be requested after patients complete the study. The following sentences were added to the end of the first paragraph:

However, we would like to continue to collect some information about how you are doing for as long as you are willing to let us. The researchers handling this study at your hospital will collect this information by looking at your medical record, or if you have not visited the hospital recently, by calling you on the telephone once a year to see how you are doing. If they need to call you on the telephone, the call would last about 5 minutes.

<u>Minor corrections:</u>

- In the first paragraph of the "What are the costs?" section, changed ~~you~~ to ***your (page 47)***
- In the "Where can I get more information?" section, corrected the following link (removed a space): <u>http://www.ClinicalTrials.gov</u> (page 48)

Questionnaire Changes

MIPE Rating Questionnaire - parent AND patient versions	• Newly created questionnaires for EDU participants to rate the MIPE program (completed by designated parent/caregiver and patients ≥ 12 years)
Acculturation questionnaire	• Added a row so that CRA can document who is completing the questionnaire
Demographics Questionnaire	• Added "Unemployed" as an option in Q12
Intervention Rating Questionnaire – parent and patient versions	• Minor changes to formatting<br>• Parent Version: Revised title of questionnaire to "Intervention Rating Questionnaire for Parents/Caregivers (IP arm)"<br>• Patient Version: Revised title of questionnaire to " Intervention Rating Questionnaire for Patients - IP arm; (To be completed by patients aged 12 years and older)"
Abbreviated Adherence Questionnaire – parent AND patient versions	• Minor changes to formatting<br>• Added questions 4-10 to see how patients are taking 6MP
Comprehensive Adherence Questionnaire – parent AND patient versions	• Minor changes to formatting<br>• Added questions 4-10 to see how patients are taking 6MP<br>• Added "Not Applicable – just received a MEMS cap today" as a choice for Q12<br>• Added "None of the above" as a choice for Q19<br>• Revised wording and answer choices for Q20<br>• Revised wording of Q21<br>• Q27: changed "self" to "Myself" in patient version and "My child" in parent version

Case Report Form Changes

Day 29 Documentation Form	• Previously the "Intervention Documentation" form<br>• Added section for CRAs to document the MIPE viewing for EDU participants<br>• Added questions to document which version of the MIPE program was viewed
Day 141 Lab Form	• Newly created form to document the labs performed on Day 141 (CBC and LFTs)
Follow-up Worksheet	• Newly created form since participants will now be followed after they complete the study for death, relapse, or the development of a SMN
Disease History and Therapeutic Summary	• Revised Q2 to include additional race/ethnicities<br>• Updated values for Q13 and Q14 to be consistent with current terminology<br>• Q25-27 re-numbered as Q25a-Q27a<br>• Added questions to document Days 8, 15, and 29 MRD (Q25b-Q27b)<br>• Added additional choices for usual care practices for medication adherence
Maintenance Report Worksheet	• Removed "not applicable" as an answer choice from Q34
Off-Study Form	• Minor changes to the formatting<br>• Added SMN option with date of SMN and SMN diagnosis
Study Participation Worksheet	• Minor changes to the formatting<br>• Added Asian and Other race/ethnicity categories
Study Refusal Form	• Changed MRN to Institution<br>• Added Asian and Other race/ethnicity categories<br>• Added "(if ≥ 12 years)" to Preferred language of patient
Eligibility and OnStudy eRDE forms	• These will be revised to be consistent with the changes in the amendment

Information Sheets

The following instruction sheets have been created:

- Intervention Instructions for Healthcare Providers
- MIPE EDU Training for Parents Unable to Attend Day 29 Clinic Visit
- Parent/Caregiver Intervention Instructions

The following instruction sheets have been revised:

- Intervention Training for Parents Unable to Attend Day 29 Clinic Visit
- Pharmacy Instructions
- Track Cap Patient Instructions

The eligibility and On Study CRFs will be posted <u>next week</u> with a separate memo.

CHILDREN'S ONCOLOGY GROUP

The world's childhood cancer experts

Group Chair
Peter C. Adamson, M.D.
adamson@email.chop.edu

Group Vice Chair
Susan Blaney, M.D.
smblaney@txch.org

Chief Operating Officer
Elizabeth O'Connor, M.P.H.
econnor@childrensoncology group.org

Chief Administrative Officer
Maria Hendricks, M.S.N, R.N. C.C.R.P.
hendricksm@email.chop.edu

Group Statistician
Meenakshi Devidas, Ph.D.
mdevidas@cog.ufl.edu

Associate Group Statistician
Todd Alonzo, Ph.D.
talonzo@childrensoncology group.org

Group Chair's Office
The Children's Hospital of Philadelphia
3501 Civic Center Blvd
CTRB 10060
Philadelphia, PA 19104

P 215 590 6359
F 215 590 7544

Group Operations Center
222 E. Huntington Drive
Suite 100
Monrovia, CA 91016

P 626 447 0064
F 626 445 4334

Statistics & Data Center Headquarters
222 E. Huntington Drive
Suite 100
Monrovia, CA 91016

P 626 447 0064
P 626 445 4334

Gainesville Office
6011 NW 1st Place
Gainesville, FL 32607

P 352 273 0556
F 352 392 8162

A National Cancer Institute supported clinical cooperative group and Equal Opportunity Affirmative Action Institutions

Memo

To: Principal Investigators and Clinical Research Associates

From: Lanie Lindenfeld, Protocol Coordinator

Re: **ACCL1033**, *A Comprehensive Approach to Improve Medication Adherence in Pediatric ALL*

Date: Monday, July 7, 2014

X	**AMENDMENT (#2)**	____	**STATUS CHANGE**
X	Change of Participants/Coordinator(s)	____	Closure
X	Editorial or Administrative Changes	____	Partial Closure
X	Scientific Changes*	____	Temporary Closure
____	Therapy Changes*	____	Reactivation
X	Eligibility Changes*		
X	Informed Consent Changes*		

***FOR THE PURPOSE OF INSTITUTIONAL PERFORMANCE ASSESSMENT, THIS AMENDMENT WILL REQUIRE SUBMISSION TO AND APPROVAL BY IRBs WITHIN 90 DAYS.**

The main goals of amendment #2 are summarized below, followed by details of all the changes.

1. Participants will be asked to complete Depression and Parental Anxiety questionnaires; Asian participants will complete an Asian Acculturation questionnaire.

Depressive symptoms (and not anxiety) are associated with poor adherence; depressive symptoms may impair cognitive focus, energy, motivation, and affect the patients'/parents' ability to follow instructions. In addition, parental distress may play a role in adherence. We will now measure depression and parental distress one time during the study, using standardized inventories, as described in detail below.

Acculturation refers to the extent to which people assume traits of mainstream culture over their traditional culture. Level of acculturation may help in enhancing adherence. We will now measure acculturation in Asian participants.

NOTE to CRAs and Study Staff regarding the Depression Questionnaires: The BDI-II and CDI 2 (patient self-report version, patients 12-17 years) contain items assessing suicidal ideation. The CRAs at the participating sites will review the responses to these questions at the time of questionnaire completion, and will alert the patient's clinician immediately should the patient and/or parent's response indicate active suicidal ideation (i.e., BDI-II, Item #9 - response #2 or #3 or CDI 2 self-report, Item # 8 - response #3). The clinician will then further evaluate the patient/parent and initiate an emergent referral to psychosocial services as indicated. (See details in Protocol Section 4.1.3.6).

2. **This study received funding from the NCI and requires a Data Safety Monitoring Plan.** As this is a low risk behavioral intervention not falling under the purview of the Children's Oncology Group Data Safety Monitoring Committee (DSMC), the DSMC of record will be located at the study coordinating center (City of Hope).

3. **The secondary aims of the study were revised.**
The secondary aim comparing the EDU arm to an un-intervened historical control (HC) group drawn from the COG AALL03N1 study was removed since the efficacy of the education intervention alone (EDU) will already be determined in the two-arm randomized controlled trial (IP vs. EDU). In addition, we are now looking at the modifying effect of psychosocial factors on adherence as depressive symptoms and parental stress may make parents and or patients less likely to respond to the intervention.

4. **With the revision of the study aims, the statistical analysis section and sample size have been modified. The accrual has increased from 570 to 608 participants.** The detailed analytic plan is described below and in Section 5 of the protocol.

5. **Other minor changes to protocol and related study documents as noted below.**

PROTOCOL REVISION LIST

*The specific changes to the protocol are shown below; text that has been deleted appears with ~~strikethrough~~, and text that has been inserted appears in **bold**.*

Header

- Updated the header from the ~~CureSearch~~ logo to **Children's Oncology Group** logo.

Title Page (Page 1)

- The version date and amendment number have been updated.
- Deleted the statistics and data center contact website.

Table of Contents (Page 2)

- The table of contents has been updated to reflect changes to the amended document.

Study Committee (Pages 3-4)

- The study committee contact information has been updated as needed.

Abstract (Page 6)

- Deleted ~~(Bhatia S. personal communication)~~ from the 3rd paragraph and added reference number ***8***.

- Deleted ~~along with socioeconomic status,~~ from the 1st sentence of the last paragraph

Experimental Design Schema (Page 7)

- The schema has been updated to reflect the changes in this amendment. See the tracked changes version of the amendment for greater detail.

Section 1.0 (Pages 8-9)

The secondary aims and Table 1 have been updated to reflect the revised study design. The new sections are shown below:

Secondary Aims

Secondary Aim 1: ***Examine the modifying effect of sociodemographic and psychosocial variables, and the mediating effect of health beliefs/ knowledge on change in adherence with intervention***

Hypothesis S1: ***Certain subgroups will derive more benefit from IP (Table 1: Modifiers): adolescents; racial/ ethnic minority patients; patients from single parent households; parents/patients with absence of depressive symptoms; parents with low levels of distress; and families with low acculturation levels. Changes in adherence with MIPE will be mediated (for both IP and Edu) by changes in health knowledge/ beliefs (Table 1: Mediators): perceived severity of illness, knowledge regarding purpose of 6MP, perception of self-efficacy to overcome barriers. The impact of MIPE as a mediator will be stronger in IP arm because of the additional behavioral reinforcement by text message reminders and DST***

Table 1. Modifiers and mediators of intervention on change in adherence

Modifiers	***Rationale***
≥12 years of age	***Lack of parental supervision contributes to non-adherence, more likely to forget; more likely to respond to intervention***
Racial/ ethnic minorities	***SES/ linguistic issues create barriers to access; influence understanding of treatment; more likely to respond to intervention***
Single parent household	***Lack of supervision contributes to non-adherence, more likely to forget; more likely to respond to intervention***
Depressive symptoms in parent/ child	***Depressive symptoms impair cognitive focus, energy, motivation; less likely to respond to intervention***
Parental distress	***Parental distress may impair focus, and affect patients'/ parents' ability to follow instructions less likely to respond to intervention***
Low levels of acculturation	***Impaired understanding re role of 6MP; lack of communication with healthcare system; more likely to respond to intervention***
Mediators	***Rationale***
Underestimation of severity of illness	***Change in knowledge re severity of illness/ likelihood of relapse if 6MP not taken as prescribed will help improve adherence***
Lack of knowledge re purpose of 6MP	***Change in understanding regarding how 6MP works in preventing relapses will help improve adherence***
Low self-efficacy to overcome barriers	***Improvement in confidence in the ability to carry out instructions will help improve adherence***

Secondary Aim 2: Determine impact of IP vs. Edu on risk of relapse of ALL

Hypothesis S2: ***IP will result in lower risk of relapse compared with Edu – upon mature follow-up of the two arms***

Section 2.5.1 (Page 12)

- Added ***Asians*** throughout section since Asian patients will also be completing an acculturation questionnaire

New Section 2.5.2 (Page 12)

- Added the below section:
  2.5.2 Depression and Parental Distress
  Depressive symptoms (and not anxiety) are associated with poor adherence.[25] Depressive symptoms may impair cognitive focus, energy, and motivation, and affect the patients' and/or parents' ability to follow instructions. Given the burden of responsibility borne by the parents in taking care of a child with a potentially life-threatening illness, parental distress may also play a role in adherence.[26] Depression and parental distress will be included in the analysis to determine the impact of these conditions on adherence.

Section 3.1.3(Page 15)

- Added the following sentence to reinforce when patients should be enrolled: ***Patients must NOT be enrolled until they have their 6MP dispensed in the MEMS® medication bottle (with a standard child resistant cap fitted to the MEMs bottle).***

Section 3.2.1 (Page 15)

- Inclusion #2 was deleted and subsequent criteria were renumbered: ~~Age ≤ 25 years at the time of study enrollment~~
- The following criteria were revised:
  - ***At the time of enrollment, patient must have completed*** ~~Has completed~~ at least 24 weeks ~~(6 months)~~ of maintenance chemotherapy, and is scheduled to receive at least ~~6~~ ***24*** more ~~months~~ ***weeks*** of maintenance chemotherapy
  - Receiving ***continuous*** oral 6MP during the maintenance phase of therapy for ALL ***(held only for toxicity or illness), and will be returning to the clinic every 4 weeks for scheduled appointments while enrolled on COG AALL1033 (between Days 1 and 141).***

Section 4.1.1 (Pages 16-17)

- Added the following to the end of the second paragraph: ***The coordinating center will also mail the BDI-II and CDI-II depression questionnaires that will be completed on study Day 1.***
- Revised the following sentence to provide more instruction: CRAs will need to enroll the patient in the correct stratum, using the self-reported race/ethnicity of the patient (African-American, Asian, Caucasian, Hispanic, and Other***; as reported on the Demographics Questionnaire***) and age of the patient (< 12 years, ≥ 12 years ***as of day of enrollment***).
- Revised the description of the Other race/ethnicity:

Other: Patients of who do not self-report race/ethnicity in the categories listed above ***or who select two of the race/ethnicity categories listed above*** (multi-ethnic/multi-racial, Native American, etc.)

- Revised the first sentence of the third from last paragraph: On the next business day following enrollment***, the CRA will contact the designated parent/caregiver to confirm the use of the TrackCap. If the CRA is unable to contact the designated parent/caregiver the following day, the CRA will continue calling daily until contact is made.***

Section 4.1.3 (Page 18)

- Deleted the last two sentences and added: ***See Table 3 for the questionnaire collection schedule.***

Section 4.1.3.5 (Page 19)

- The section title was renamed: Acculturation Questionnaire (Hispanic ***and Asian*** participants only).
- Added this sentence to the beginning of the first paragraph: ***Acculturation questionnaires will be collected from Hispanic and Asian participants.***
- In the first paragraph, added ***Hispanic*** when describing the acculturation questionnaire
- Added the second paragraph to describe the Asian acculturation questionnaire: ***Asian patients (if ≥ 12 years) and their parents/caregivers will be asked to complete the Suinn-Lew Asian Self Identity (SL-Asia) Acculturation Questionnaire on Day 1 in order to determine the level of acculturation of Asian participants. The SL-Asia Acculturation Questionnaire[24] consists of 26 items (multiple choice and 5-point Likert format) that ask about the participant's historical background as well as recent behaviors that may be related to cultural identity. A score of 1 indicates a low level of acculturation and 5 a high level of acculturation. Estimated completion time is 10 minutes.***

Sections 4.1.3.6 (Page 19) and 4.1.3.7 (Pages 19-20)

- Newly added sections to describe the Depression and Parental Distress Questionnaires:

4.1.3.6 Depression Questionnaires

The designated parent/caregiver and patient (if ≥ 12 years) will be asked to complete a questionnaire to measure depression during their regularly scheduled clinic visit on Study Day 1. Depressive symptoms (and not anxiety) are associated with poor adherence;[25] depressive symptoms may impair cognitive focus, energy, motivation, and affect the patients'/parents' ability to follow instructions. The designated parent/caregiver and patients 18 years and older will complete the Beck Depression Inventory-II (BDI-II).[41,42] Adolescent patients (12-17 years) will complete the Children's Depression Inventory-2 (CDI-II) self-report version; and for younger patients (<12 years), the designated parent/caregiver will complete the CDI-II parent-report version.[43] English and Spanish versions are available. Estimated completion time is 5 minutes.

Additionally, the BDI-II and CDI-II (patient self-report version, patients 12-17 years) contain items assessing suicidal ideation. The CRAs at the participating sites will review the responses to these questions at the time of questionnaire completion, and will alert the patient's clinician immediately should the patient and/or parent's response indicate active suicidal ideation (i.e., BDI-II, Item #9 - response #2 or #3 or CDI-II self-report, Item # 8 - response #3). The clinician will then further evaluate the patient/parent and initiate an emergent referral to psychosocial services as indicated.

The BDI-II and CDI-II questionnaires are licensed and may not be posted in the questionnaire packet on the COG website. The coordinating center will mail these questionnaires when the MEMS®TrackCap ™ is requested (at the time of consent).

4.1.3.7 Parental Distress Questionnaires

The designated parent/caregiver will be asked to complete a questionnaire to measure parental distress during their regularly scheduled clinic visit on Study Day 85. Parental distress may play a role in adherence. We will measure parental distress using the Pediatric Inventory for Parents (PIP)[26] – a parenting burden measure for parents of children with cancer/other chronic illnesses. It captures parental distress in 4 domains (Communication, Emotional Distress, Medical Care, Role Function), and has also been validated in Spanish.[44] Estimated completion time is 10 minutes.

Table 3 (Page 20)

- Deleted ~~Data~~ from the title and added ***Questionnaire.***
- Added additional instructions regarding the Demographics questionnaire.
- Added new questionnaires introduced in this amendment.

Section 4.1.4 (Page 21)

- Added additional instruction in the first paragraph: The CRA will follow-up by phone with the parent/caregiver on the next business day following enrollment, to confirm that the *TrackCap*™ is being used or if not, to identify obstacles to *TrackCap*™ use and determine solutions ***(if CRA is unable to reach the parent/caregiver on the next business day, the CRA will continue calling daily until contact is made).***

Sections 4.1.5.1.1(Page 23) and 4.1.5.2.4 (Page 25)

- Added the following sentence to the end of the section: ***If the CRA is unable to reach the designated parent/caregiver on Day 29 and/or for the second follow-up call, the CRA will continue calling daily until contact is made.***

Section 4.2 (Page 25)

- Deleted the mailing address. All study forms should be faxed or emailed to the Coordinating Center:

  ~~Dr. Smita Bhatia~~
  ~~City of Hope~~
  ~~1500 East Duarte Rd., DPS-173~~
  ~~Duarte, CA 91010~~
  ~~(626) 256-HOPE (4673) ext. 61189~~

- Deleted ~~Or~~ and added ***Fax:*** and ***Email:*** descriptors

Table 5 (Page 26)

- Added the new questionnaires to the table.

Section 5.1 (Page 28)

- Deleted the following from the first sentence: ~~; it also determines the impact of the education program on adherence to 6MP by comparing patients on EDU to an un-intervened historical control~~

~~(HC) group drawn from the COG AALL03N1 study. In general, we anticipate the effect of EDU to be intermediate between that of IP and HC~~

- Revised Table 6 with new accrual numbers (as describe in Section 5.2)

Section 5.2 (Page 28)

- Updated the accrual from ~~570~~ to ***608*** subjects, from ~~285~~ to ***304*** in each arm.

Section 5.3 (Pages 28 and 29)

- Revised the analytic plan to account for the changes to the specific aims and sample size.
- Added the following

Eligible participants will be randomized to Edu or IP using blocked stratified randomization (10 strata) with racial/ethnic background (NHW, Hispanics, AA, Asians, mixed/other) and age at study (<12y, ≥12years) as stratification factors and a block size of 6 to balance participant number/ type in each arm.

Adherence measured by MEMS will be treated as a binomial variable, and that measured by TGN levels as a continuous variable. MEMS-based adherence rate will be defined as the ratio of days with MEMS cap openings (N) to days 6MP was prescribed (D) for each patient, reported as a percent (N/D*100). Days when 6MP was held by prescriber will be removed from the denominator. Adherence measurements will be calculated for 5 time points (Days 29, 57, 85, 113, 141), each reflecting adherence over the preceding 28 days. Intervention begins on Day 29. Since participants are randomized, baseline adherence rate (Day 29) is not expected to differ between Edu and IP. Nevertheless, we will evaluate the success of randomization by comparing baseline group differences in adherence, demographic, clinical and psychosocial variables. We will conduct propensity score analysis to adjust for imbalance, to obtain unbiased estimates of intervention effects. We will use multiple imputations for missing data before conducting propensity analyses.

Primary Aim 1: ***Determine impact of intervention package (IP) vs. education (Edu) on adherence to oral 6MP in children with ALL. Adherence measured by: i) MEMS (primary measure); and ii) red cell TGN levels***
Hypothesis 1.1: IP will result in a higher proportion of patients with adherence rates ≥95% compared with Edu. Hypothesis 1.2: IP will result in a higher proportion of ≥12years-old patients with adherence rates ≥95% compared with Edu; IP will result in a higher proportion of <12y-old patients with adherence rates ≥95% compared with Edu, allowing evaluation of the developmentally-tailored intervention

The primary outcome of this trial is the proportion of patients with adherence rate ≥95% to 6MP over the duration of the intervention. Using an intention-to-treat analysis, the effectiveness of IP will be determined by comparing the proportion of adherent patients in the IP and Edu groups using logistic regression. This test will be conducted for all ages combined (Hypothesis 1.1) and separately for <12y and ≥12years olds (Hypothesis 1.2). We will also examine longitudinal changes in red cell TGN levels between the two arms using GEE for longitudinal normally distributed data to corroborate results from the MEMS data analysis. The TGN analysis will be limited to patients with normal TPMT activity and TGN will be adjusted for 6MP dose-intensity.

Exploratory Aim 1: Examine the modifying effect of sociodemographic and psychosocial variables, and the mediating effect of health beliefs/ knowledge on change in adherence with intervention

Hypothesis E1: Certain subgroups will derive more benefit from IP (modifiers: Table 1). Changes in adherence with MIPE will be mediated by change in health knowledge/beliefs (Table 1). Impact of MIPE as a mediator will be stronger in IP because of additional behavioral reinforcement by customized printed schedules, text message reminders and DST.

Interactions of group by modifying and mediating covariates will be included in the logistic regression model and their significance tested. To better understand the effects of mediators, individuals' adherence rates (as binomial variable) will be modeled longitudinally using GEE methods (with compound symmetry as working correlation matrix) by including the interactions of treatment group by time and treatment group by time by mediator variable. This will enable examination of whether changes in proportion of adherent patients in each group resulted from changes in individuals' adherence rates as a consequence of variation in mediator variables. As power for examining interaction is expected to be low, these analyses will be exploratory.

Exploratory Aim 2: Establish the infrastructure to determine the impact of IP vs. Edu on risk of relapse in children with ALL

Hypothesis E2: IP will result in lower risk of relapse compared with Edu – upon mature follow-up of the two groups

An intention-to-treat analysis will be used to compare the effectiveness of Edu and IP interventions in decreasing the risk of relapse. Cox proportional hazards regression models will be used to examine the impact of intervention on relapse. Covariates in the analysis will include clinical and sociodemographic predictors, and the intervention arm (IP vs. Edu).

Section 5.4 (Page 30)

- Revised the sample and power section to account for the increased target accrual
- The section has been revised accordingly:

Sample size and power determination was based on binomial distribution, classifying each patient as "adherent" (adherence rate ≥95%) or "non-adherent" (adherence rate <95%). Sample size calculation was performed using NCSS PASS. Aim 1: The effectiveness of IP over Edu will be determined by comparing P_{IP} (proportion of patients with adherence rate ≥95% in IP) to P_{Edu} (the corresponding rate in Edu), by testing Ho: $P_{IP} = P_{Edu}$ in all ages combined as well as in <12y and ≥12years old. To account for 3 independent tests, a 2-sided Bonferroni-adjusted Type I error=0.017 (for an overall Type I error=0.05) will be used for each test. Since the value for P_{Edu} is unknown, we assumed its value to be at least that observed under usual care in AALL03N1 and increased it up by +5% and +10%. Thus, for <12y, we let P_{Edu} =0.60 (observed in AALL03N1), 0.65, 0.70. For ≥12years, we let P_{Edu} =0.42, 0.47, 0.52. For both age groups combined, we let P_{Edu} =0.56, 0.61, 0.66. Of note, these values included patients whose estimated adherence rates were 100% in AALL03N1. When both age groups are combined (Hypothesis 1.1), the sample size of 456 (228 per arm) has 80% power to detect a difference between P_{Edu} =0.56 to 0.66 and P_{IP} =0.70 to 0.79 (i.e., 70%-79% of IP patients have adherence rates ≥95% vs. 56%-66% Edu patients), or an Odds Ratio (OR) =1.9-2.0. Under Hypothesis 1.2, for <12y, 133 patients/arm will provide 80% power to detect a difference between P_{IP} =0.78-0.86 and P_{Edu} =0.60-0.70, or an OR =2.4-2.7. For ≥12years, 95 patients/arm will provide 80% power to detect

a difference between P_{IP} =0.65-0.74 and P_{Edu} =0.42-0.52, or an OR =1.9-2.0. Thus, this study is powered to detect a minimum improvement of 17 to 23 percentage points in the proportion of adherent patients with the intervention. This effect size is commensurate with published intervention studies that demonstrate an effect size range of 17.8%[46] to 46%.[34]

Exploratory Aim 1: Adequate power for detecting a modifier/mediator of treatment effect requires a larger sample size; analysis will therefore be considered exploratory. Exploratory Aim 2: At 2-sided Type I error of 0.05, assuming a 10% relapse rate over 5y of follow-up, there is 80% power to detect a hazard ratio of 2.7.

Feasibility of achieving target enrollment: In the observational trial (AALL03N1) the attrition rate was 16%. We have inflated the target enrollment from 456 (evaluable patients needed for Aim 1) to 608 (accounting for a higher 25% attrition anticipated in an intervention trial). Table 6 demonstrates the number of patients needed by race/ethnicity within each age group (based on AALL03N1 age distribution).

Section 5.5 (Page 31)

- The gender and minority accrual estimates were updated based on the increased target accrual.

Section 6.0 (Page 31)

- Added this section, Data Safety and Monitoring, since the City of Hope Data Safety and Monitoring Committee will be responsible for monitoring the conduct of the study:

6.0 DATA SAFETY AND MONITORING

As this is a low risk behavioral intervention not falling under the purview of the Children's Oncology Group Data Safety Monitoring Committee (DSMC), the DSMC of record will be located at the study coordinating center (City of Hope). The Study PI (Smita Bhatia) will be responsible for monitoring protocol conduct and reporting any deviations, unanticipated problems, adverse events and/or serious adverse events related to protocol intervention and/or research procedures performed on this study to the City of Hope DSMC. If a patient experiences any adverse event, serious adverse event, or unanticipated problem related to their participation in ACCL1033, you must notify the Coordinating Center (AdherenceStudy@coh.org, (626) 256-4673 x61189) within the reporting timeframes described in Appendix IV. Please note that adverse events, serious adverse events and unanticipated problems related to the patient's ALL treatment protocol do not have to be reported to the ACCL1033 Coordinating Center.

The coordinating center will report study deviations from all COG participating institutions, including missing or overdue data, to the City of Hope DSMC. Due dates for all study requirements are listed in Appendices II and III.

Appendix I (Pages 32-36)

- Added ***",how you are feeling,"*** to number 3 on the information sheets for children 7-11 years and 12-17 years of age
- Revised number 3 of the parent/caregiver information sheet:

You and your child (if ≥ 12 years old) will be asked to complete ~~8~~ ***9*** to ~~10~~ ***11***questionnaires over the course of the study. These questionnaires ask about your family (sociodemographic information, such as the number of people in your household, your educational level, and income)***, how you and your child are feeling,*** and how your child is doing with taking their 6MP. If you/your child is Hispanic ***or Asian***, you will also be asked to complete a questionnaire about language preferences.

Appendix II (Page 37 and 38)

- Added the new questionnaires (Asian Acculturation, BDI-II/CDI 2 Depression, Parental Distress).
- Added the Follow-up forms (this form was added in Amendment 1, but not updated on this table).
- Revised the due dates.
- Added the following footer:
  **** For the purposes of data submission, the due dates are based upon the ACCL1033 study timepoints, which do not necessarily correspond to actual calendar days. For example, it is possible to have a Study Day 29 that is 35 days from the Day 1 appointment (if patient rescheduled or delayed their appointment one week). The coordinating center will also report study deviations from all COG institutions, including missing or overdue data, based upon the due dates provided in this table. Patient and/or parent refusal of any study requirement will not be reported as a deviation.***

Appendix III (Page 39)

- Corrected the phone number for the Clinical Pharmokinetics Laboratory at St. Jude.
- Added the Coordinating Center email as an option to email the completed lab form.
- Added the following footer:
  **** If the Day 1 TPMT sample is missed or within 90 days of a RBC transfusion, it may be obtained at anytime during the study. If the sample is not obtained for any other timepoint, the sample may be collected within one week of the corresponding study timepoint. Otherwise it will be reported as a deviation. Patient and/or parent refusal of any study blood draw will not be reported as a deviation.***

Appendix IV (Pages 40 and 41)

- Added this section to provide definitions and reporting guidelines for reporting Adverse Events, Serious Adverse Events and Unanticipated Problems to the Data Safety Monitoring Committee (reported to the Coordinating Center, who will report to the COH DSMC as specified in Section 6).

Reference Section (Pages 42-44)

- The reference section was revised to reflect the new references added with this amendment (added references 8, 24, 25, 26, 34, 41, 42, 43 47).

INFORMED CONSENT REVISION LIST

What will happen on this study that is research? (Study activities for all participants section) Page 47

- Clinic visit # 2: revised first sentence of second paragraph: At various times during this study, you will be asked to complete ***9***~~8~~ to ***11***~~10~~ questionnaires (how many depends on your ethnicity ~~and which study arm you are on~~).

What will happen on this study that is research? (Research Measurements Section) (Pages 49 and 50)

- Added How You Are Feeling Questionnaire (Depression questionnaire):
***How You Are Feeling Questionnaire*: We will ask you (if 12 years of age or older) and your parent/caregiver questions about how you are feeling at clinic visit #2 for the study. If you are under age 18, your parent/caregiver will complete this questionnaire. The questionnaire will take about 5 minutes to complete.**
- Added additional questionnaire for parent/caregivers (Parental Stress Questionnaire):
Additional Questionnaire for parent/caregivers ONLY:
We will also ask your parent/caregiver to complete a parental stress questionnaire during clinic visit #5. This questionnaire will ask about take about 10 minutes to complete.
- Added ***Hispanic*** to Acculturation Questionnaire
- Added Asian Acculturation Questionnaire
Additional Questionnaire for participants who are Asian:
We will also ask you (if 12 years of age or older) and your parent/caregiver to complete the following at clinic visit #2 for the study:
Asian Acculturation Questionnaire: We will ask you and your parent/caregiver questions such as what language you usually use to read, speak and think in; in what language you prefer to watch television and listen to the radio; and what language your friends use. This questionnaire will take about 5 minutes to complete.

What side effects or risks can I expect from being in the study? (Page 51)

- Deleted second sentence: ~~You may have some discomfort answering questions about your medication-taking behavior~~
- Added the following 3 sentences: ***Some of the questions in the questionnaires are of a personal nature and may make you feel uncomfortable. The questionnaires that are being used for this research study have been used many times by many hospitals and clinics. To the best of our knowledge they have not caused anyone serious problems. You do not have to answer any question that you don't want to.***
- Separated the risks from blood draws
  - Revised first sentence: **~~Also, b~~ *B*lood drawing may cause pain…**
  - Revised the second sentence: ***The study-related blood tests will be in the form of an extra tube, with no addition need sticks,*** and there are no additional risks…

How many people will take part in the study? (Page 51)

- Revised the total number from ~~570~~ to ***608.***

CRFs, Questionnaires and Instructions

The following forms are new:

The following forms have been revised due to amendment #2:

1. Eligibility CRF
2. On Study CRF
3. Disease History and Therapeutic Summary CRF
4. Lab CRF
5. Maintenance Report Form CRF
6. Eligible Patient Participation Worksheet CRF
7. Study Chair/Central Reviewer Case Report forms (above CRF packet)
8. Hispanic Acculturation Questionnaire
9. Intervention Rating-Parent Questionnaire
10. Intervention Rating-Patient Questionnaire
11. MIPE Rating-Parent Questionnaire
12. MIPE Rating-Patient Questionnaire
13. Intervention Training for Parents Unable to Attend Day 29 Clinic Visit (Instructions)
14. MIPE EDU Training for Parents Unable to Attend Day 29 Clinic Visit (Instructions)
15. CRA Study Checklist (Instructions)

The following forms have not changed:

1. Day 29 Documentation CRF
2. Follow up Worksheet CRF
3. Day 141 Lab
4. Off Study CRF
5. Study Refusal CRF
6. Demographics Questionnaire
7. Abbreviated Adherence-Parent Questionnaire
8. Abbreviated Adherence-Patient Questionnaire
9. Comprehensive Adherence-Parent Questionnaire
10. Comprehensive Adherence-Patient Questionnaire
11. Trackcap patient Visit (Instructions)
12. CRA MIPE (Instructions)
13. Accessing the MIPE Website (Instructions)
14. Day 29 Education Checklist (Instructions)
15. Day 29 Intervention Checklist (Instructions)
16. Intervention Instructions for Healthcare Provider (Instructions)
17. Pharmacy(Instructions)

The following Spanish forms are currently unavailable until the translations can be finalized:

1. Intervention Rating-Parent Questionnaire
2. Intervention Rating-Patient Questionnaire
3. MIPE Rating-Parent
4. MIPE Rating-Patient
5. Intervention Training for Parents Unable to Attending Day 29 Clinic Visit (Instructions)
6. Intervention Training for Parents Unable to Attend Day 29 Clinic Visit (Instructions)
7. ParentCaregiver Intervention Instructions Under 12 (Instructions)
8. ParentCaregiver Intervention Instructions Under 12-17 (Instructions)
9. ParentCaregiver Intervention Instructions Under 18+ (Instructions)

CHILDREN'S ONCOLOGY GROUP

The world's childhood cancer experts

Group Chair
Peter C. Adamson, M.D.
adamson@email.chop.edu

Group Vice Chair
Susan Blaney, M.D.
smblaney@txch.org

Chief Operating Officer
Elizabeth O'Connor, M.P.H.
econnor@childrensoncology group.org

Chief Administrative Officer
Maria Hendricks, M.S.N, R.N. C.C.R.P.
hendricksm@email.chop.edu

Group Statistician
Meenakshi Devidas, Ph.D.
mdevidas@cog.ufl.edu

Associate Group Statistician
Todd Alonzo, Ph.D.
talonzo@childrensoncology group.org

Group Chair's Office
The Children's Hospital of Philadelphia
3501 Civic Center Blvd
CTRB 10060
Philadelphia, PA 19104

P 215 590 6359
F 215 590 7544

Group Operations Center
222 E. Huntington Drive
Suite 100
Monrovia, CA 91016

P 626 447 0064
F 626 445 4334

Statistics & Data Center Headquarters
222 E. Huntington Drive
Suite 100
Monrovia, CA 91016

P 626 447 0064
F 626 445 4334

Gainesville Office
6011 NW 1st Place
Gainesville, FL 32607

P 352 273 0556
F 352 392 8162

A National Cancer Institute supported clinical cooperative group and Equal Opportunity Affirmative Action Institutions

Memo

To: Principal Investigators and Clinical Research Associates

From: Meg Stahlman, Protocol Coordinator

Re: **ACCL1033**, *A Comprehensive Approach to Improve Medication Adherence in Pediatric ALL*

Date: Monday, June 15, 2015

X	**AMENDMENT #3**	____	**STATUS CHANGE**
X	Change of Participants/Coordinator(s)	____	Closure
X	Editorial or Administrative Changes	____	Partial Closure
X	Scientific Changes*	____	Temporary Closure
____	Therapy Changes*	____	Partial Reactivation
____	Eligibility Changes*		
X	Informed Consent Changes*		

***FOR THE PURPOSE OF INSTITUTIONAL PERFORMANCE ASSESSMENT, THIS AMENDMENT WILL REQUIRE SUBMISSION TO AND APPROVAL BY IRBs WITHIN 90 DAYS.**

The main goals of this amendment are summarized below:

1. The study chair, Dr. Smita Bhatia, has relocated from City of Hope to Children's of Alabama at the University of Alabama Birmingham (UAB). As a result, the ACCL1033 coordinating center is transferring from City of Hope to Children's of Alabama/UAB.

2. We have revised the target accrual distribution by combining the Asian and Other strata into one stratum. The decision to combine these strata was made in response to the following two observations: i) The significant under-enrollment by both the Asian and Other categories; ii) the significant heterogeneity observed among the Asians in the predecessor study (AALL03N1) precluding any meaningful interpretation of data when considering "Asians" as a single entity. Therefore, to reach accrual for the study within the 48 month timeframe, we are combining the Asian and Other strata into one stratum, Asian/Other, with a target accrual of 80. To maintain the target accrual of 608 participants, for which the study is powered, we are increasing the target accrual of the non-Hispanic white and Hispanic strata from 184 to 224 participants.

Please refer to the Summary of Changes list provided below for a description of all amendment updates.

Note: Additions are indicated in **boldface** and deletions are indicated in ~~strikethrough~~ font.

PROTOCOL CHANGES

The following contact information for the coordinating center was updated throughout the protocol as described in the table below. All references to City of Hope were deleted or updated to UAB. **PLEASE MAKE NOTE OF THIS NEW CONTACT INFORMATION.** There will be a transition period where staff will be available at both COH and UAB. However, please start contacting the coordinating staff at UAB with any questions or supply requests.

	Previous Version	Amended Version
Coordinating Center	~~City of Hope (COH)~~	**Children's of Alabama/(UAB)**
Phone	~~(626) 256-4673 x61189~~	**(205) 638-2129**
Email	~~AdherenceStudy@coh.org~~	**AdherenceStudy@peds.uab.edu**
Fax	~~(626) 930-5387~~	**(205) 212-3004**
Mailing Address	~~City of Hope~~<br>~~1500 E. Duarte Rd, Building 173~~<br>~~Duarte, CA 91010~~	**UAB Division of Peds Hem/Onc**<br>**1600 7th Ave S, Lowder 500**<br>**Birmingham, AL 35233-1711**

Header and Footer

- The version date of the protocol was updated

Title Page (Page 1)

- The version date and amendment number have been updated
- The study chair contact information has been updated

Table of Contents (Page 2)

- The table of contents has been updated to reflect changes to the amended protocol.

Study Committee (Pages 3-4)

- The study committee contact information has been updated as needed.

Section 2.2.2: Electronic Medication Monitoring (Page 9)

- Updated the company information for the MEMS cap from ~~Aprex Corp, Fremont, CA~~ to **MWV Healthcare, Switzerland**.

Section 2.6: Adherence to Oral 6MP and Survival (Page 12)

- Updated the reference at the end of the section from ~~(Bhatia S. personal communication)~~ to **8** since the information has since been published.

Section 3.1.4: Randomization (Page 15)

- Revised the strata: Randomization, stratified by ethnic background (**4**~~5~~ strata: Hispanic, non-Hispanic white, African-American, **and** Asian/ ~~, and~~ Other)

Section 4.1.1: Randomization (Page 17)

- Revised race/ethnicity strata:
  - CRAs will need to enroll the patient in the correct stratum, using the self-reported race/ethnicity of the patient (African-American, ~~Asian,~~ Caucasian, Hispanic, **or Asian**/Other; as reported on the Demographics Questionnaire)…
  - Combined the Asian and Other race/ethnicity definitions

- In the last paragraph on page 17, revised the wording of calling the patient and completing the OnStudy form: After **calling to** confirm~~ing~~ use of the TrackCap, the CRA will complete the OnStudy eRDE form…

Section 5.1: Statistical Design (Page 28)

- Revised Table 6 with new patient accrual numbers:

	**<12 years (accounting for attrition)**	**≥12 years (accounting for attrition)**	**Total number needed for trial**	**Total number to be enrolled to account for attrition**
Non-Hispanic white	~~108~~ **132**	~~76~~ **92**	~~138~~ **170**	~~184~~ **224**
Hispanic	~~108~~ **132**	~~76~~ **92**	~~138~~ **170**	~~184~~ **224**
African American	48	32	58	80
Asian/**Other**	48	32	58	80
~~Other~~	~~48~~	~~32~~	~~64~~	~~80~~
Total	360	248	456	608
See Section 3.1.1 for the race/ethnicity definitions				

Section 5.3: Analytic Plan (Page 28)

- Updated first sentence: Eligible participants will be randomized to EDU or IP using blocked stratified randomization (~~10~~ **8** strata) with racial/ethnic background (NHW, Hispanics, AA, Asians ~~,mixed~~ /other)…

Section 5.5: Gender and Minority Accrual Estimates (Page 31)

- Revised the table with new patient accrual numbers, to account for the combined Asian and Other racial categories, and to increase accrual for Hispanic and Non-Hispanic whites

Section 6.0: Data Safety and Monitoring (Page 31)

- Since the coordinating center is moving to Children's of Alabama/UAB, the DSMC of record will also move. The data safety and monitoring section has been updated accordingly:
  As this is a low risk behavioral intervention not falling under the purview of the Children's Oncology Group Data Safety Monitoring Committee (DSMC), the DSMC of record will be located at the study coordinating center (~~City of Hope~~ **Children's of Alabama/UAB**). The Study PI (Smita Bhatia) will be responsible for monitoring protocol conduct and reporting any deviations, unanticipated problems, adverse events and/or serious adverse events related to protocol intervention and/or research procedures performed on this study to the ~~City of Hope~~ **Children's of Alabama/UAB** DSMC. If a patient experiences ~~any adverse event,~~ **a** serious adverse event, or unanticipated problem related to their participation in ACCL1033, you must notify the coordinating center (~~AdherenceStudy@coh.org~~ AdherenceStudy@peds.uab.edu, (~~626) 256-4673 x61189~~ (205) 638-2129) within ~~the reporting timeframes described in Appendix IV~~ **5 calendar days**. **Adverse events related to ACCL1033 participation that do not meet the criteria of serious OR are not**

unanticipated problems should be reported to the study coordinating center as soon as possible and no later than 4 weeks following the event. Please note that adverse events, serious adverse events and unanticipated problems related to the patient's ALL treatment protocol do not have to be reported to the ACCL1033 coordinating center.
~~The coordinating center will report study deviations from all COG participating institutions, including missing or overdue data, to the City of Hope DSMC. Due dates for all study requirements are listed in Appendices II and III.~~

APPENDICES

- Appendix II
  - Updated contact information for the coordinating center
  - Updated the due date for the adherence and intervention rating questionnaires: As soon as possible and no later than 2 weeks ~~of~~ **from the time** the questionnaire ~~completion date~~ **is completed and returned**
  - Deleted part of the last sentence from the footer of Appendix II: The coordinating center will also report study deviations from all COG institutions~~, including missing or overdue data, based upon the due dates provided in this table. Patient and/or parent refusal of any study requirement will not be reported as a deviation~~.

- Appendix III
  - Updated contact information for the coordinating center
  - Deleted the last two sentences from the footer of Appendix III: ~~Otherwise it will be reported as a deviation. Patient and/or parent refusal of any study blood draw will not be reported as a deviation.~~

- Appendix IV was deleted as it was specific to City of Hope Data Safety and Monitoring

INFORMED CONSENT CHANGES

- In the "Required Research Tests and Measurements" section, the age was corrected for the "How You Are Feeling Questionnaire": We will ask you (if 12 years of age or older) and your parent/caregiver questions about how you are feeling at clinic visit #2 for the study. If you are under age ~~18~~ **12**, your parent/caregiver will complete this questionnaire.

- In the "What about privacy?" section, updated the IRB and coordinating center from City of Hope to Children's of Alabama/UAB

- The Institutional Review Board of ~~the City of Hope~~ **Children's of Alabama/University of Alabama at Birmingham** ~~in Duarte, CA~~ (the location of the study coordinating center)

- ~~City of Hope~~ **Children's of Alabama/University of Alabama at Birmingham (UAB),** as the coordinating center, will receive the research information including personal information

PATIENT QUESTIONNAIRE CHANGES

- For the parent and patient adherence questionnaires (abbreviated and comprehensive versions) and the Pediatric Inventory for Parents, we added the following instructions below the first page of the questionnaire. These questionnaires are scanned and read by the computer, and should be completed in black ink. No changes were made to the questions in the questionnaires.

  Instructions for Completing the Questionnaire

  Because this is a computer read form, please follow these rules in completing this questionnaire.

  1. **Please use a pen with BLACK ink. Refrain from using a felt-tip or roller-ball pen. These may cause smudging.**
  2. **When marking boxes, make an x inside the box.**
  3. **Make no stray marks of any kind. Please keep the form as clean as possible.**

INSTRUCTIONS/CRF CHANGES

<u>Eligibility form</u>

- The stratum for both Asian and Other were combined into one.

<u>CRA Study Checklist</u>

- The contact information for the coordinating center was updated

<u>ACCL1033 Lab Requisition Form</u>

- The contact information for the coordinating center was updated

<u>ACCL1033 Follow Up Worksheet</u>

- Since this is a computer read form, added instructions to complete in black ink

<u>Pharmacy Instructions</u>

- Updated the contact information for Smita Bhatia and Wendy Landier
- Removed the information for the study pager

INTERVENTION TRAINING MODULE (POWERPOINT) CHANGES

The Powerpoint presentation will be updated for this amendment next week.
