# Supplementary material for: Effect of a Daily Text Messaging and Directly Supervised Therapy Intervention on Oral Mercaptopurine Adherence in Children With Acute Lymphoblastic Leukemia: A Randomized Clinical Trial
Source: JAMA Netw Open. 2020 Aug 27;3(8):e2014205. doi: 10.1001/jamanetworkopen.2020.14205 (PMC7453312; doi:10.1001/jamanetworkopen.2020.14205)
Supplement: Supplement 2. — eAppendix 1. Participating Children’s Oncology Group Institutions eAppendix 2. Supplemental Methods eTable 1. Median Pre- to Post-Intervention Adherence Rates by Study Arm eTable 2. Sensitivity Analysis [file jamanetwopen-3-e2014205-s002.pdf]

Supplementary Online Content

Bhatia S, Hageman L, Chen Y, et al. Effect of a daily text messaging and directly supervised therapy intervention on oral mercaptopurine adherence in children with acute lymphoblastic leukemia: a randomized clinical trial. *JAMA Netw Open*. 2020;3(8):e2014205. doi:10.1001/jamanetworkopen.2020.14205

eAppendix 1. Participating Children's Oncology Group Institutions

eAppendix 2. Supplemental Methods

eTable 1. Median Pre- to Post-Intervention Adherence Rates by Study Arm

eTable 2. Sensitivity Analysis

This supplementary material has been provided by the authors to give readers additional information about their work.

©2020. Bhatia S et al. *JAMA Network Open*.

eAppendix 1. Participating Children’s Oncology Group Institutions

#	Institution	Location
1	Alfred I duPont Hospital for Children	Wilmington, Delaware
2	Ann and Robert H. Lurie Children's Hospital of Chicago	Chicago, Illinois
3	Ascension Saint John Hospital	Gross Point Woods, Michigan
4	Baylor College of Medicine	Houston, Texas
5	C S Mott Children's Hospital	Ann Arbor, Michigan
6	Carilion Clinic Children's Hospital	Roanoke, Virginia
7	Children's Healthcare of Atlanta - Egleston	Atlanta, Georgia
8	Children's Hospital Medical Center of Akron	Akron, Ohio
9	Children's Hospital of Los Angeles	Los Angeles, California
10	Children's Hospital of Philadelphia	Philadelphia, Pennsylvania
11	Children's Hospital of Pittsburgh of UPMC	Pittsburgh, Pennsylvania
12	Children's Hospitals and Clinics of Minnesota - Minneapolis	Minneapolis, Minnesota
13	Children's National Medical Center	Washington, D.C.
14	City of Hope	Duarte, California
15	Columbia University Medical Center	New York, New York
16	Cook Children's Medical Center	Fort Worth, Texas
17	David Geffen School of Medicine at UCLA	Los Angeles, California
18	Dell Children's Medical Center of Central Texas	Austin, Texas
19	Driscoll Children's Hospital	Corpus Christi, Texas
20	Duke University Medical Center	Durham, North Carolina
21	Geisinger Medical Center	Danville, Pennsylvania
22	Helen DeVos Children's Hospital at Spectrum Health	Grand Rapids, Michigan
23	Lee Memorial Health System	Fort Myers, Florida
24	Mayo Clinic	Rochester, Minnesota
25	Memorial Healthcare System - Joe DiMaggio Children's Hospital	Hollywood, Florida
26	Mercy Hospital Saint Louis	St. Louis, Missouri
27	Miller Children's Hospital	Long Beach, California
28	Nationwide Children's Hospital	Columbus, Ohio
29	Nemours Children's Clinic - Jacksonville	Jacksonville, Florida
30	Nemours Children's Clinic - Pensacola	Pensacola, Florida
31	Nevada Cancer Research Foundation CCOP	Las Vegas, Nevada
32	New York University Langone Medical Center	New York, New York
33	Novant Health Presbyterian Medical Center	Charlotte, North Carolina
34	Ochsner Clinic Foundation	New Orleans, Louisiana

©2020. Bhatia S et al. *JAMA Network Open*.

#	Institution	Location
35	Palmetto Health Richland	Columbia, South Carolina
36	Providence Sacred Heart Medical Center and Children's Hospital	Spokane, Washington
37	Rady Children's Hospital - San Diego	San Diego, California
38	Raymond Blank Children's Hospital	Des Moines, Iowa
39	Riley Hospital for Children	Indianapolis, Indiana
40	Saint Mary's Hospital	West Palm Beach, Florida
41	Saint Vincent Hospital Cancer Center Green Bay	Green Bay, Wisconsin
42	Seattle Children's Hospital	Seattle, Washington
43	Southern California Permanente Medical Group	Los Angeles, California
44	The Children's Medical Center of Dayton	Dayton, Ohio
45	The Toledo Hospital/Toledo Children's Hospital	Toldeo, Ohio
46	Tulane University Health Sciences Center	New Orleans, Louisiana
47	University of Alabama at Birmingham	Birmingham, Alabama
48	University of Hawaii	Honolulu, Hawaii
49	University of Illinois	Chicago, Illinois
50	University of Minnesota Medical Center-Fairview	Minneapolis, Minnesota
51	University of Mississippi Medical Center	Jackson, Mississippi
52	University of New Mexico	Albuquerque, New Mexico
53	University of Oklahoma Health Sciences Center	Oklahoma City, Oklahoma
54	University of Texas Health Science Center	San Antonio, Texas
55	University of Texas Southwestern Medical Center	Dallas, Texas
56	Vannie Cook Children's Clinic	McAllen, Texas
57	Wake Forest University Health Sciences	Winston-Salem, North Carolina
58	Washington University School of Medicine	St. Louis, Missouri
59	Wayne State University	Detroit, Michigan

©2020. Bhatia S et al. *JAMA Network Open*.

eAppendix 2. Supplemental Methods

INTERVENTION COMPONENTS

Theoretical Framework

The theoretical framework for the intervention is the Extended Health Belief Model (Rosenstock, 1988). Within this framework, the likelihood of adherence to therapy is dependent upon the balance between perceived benefits of and barriers to the therapy, weighed against perceived susceptibility to and severity of the illness being treated, within the context of perceived self-efficacy of the patient/parent to adhere to the treatment. The constructs addressed by the intervention program include perceived benefits, perceived barriers, perceived susceptibility, perceived severity, and self-efficacy.

Multimedia Interactive Patient/Parent Educational Program

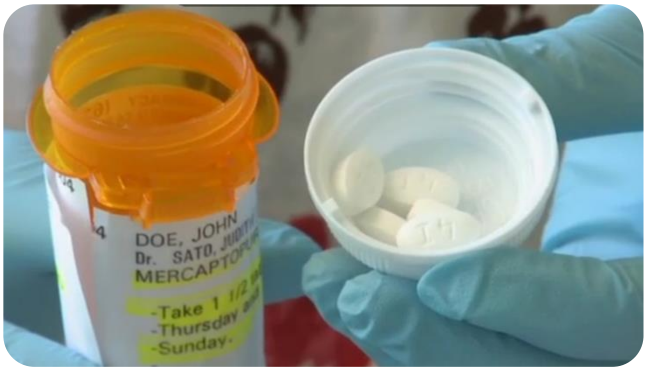

The educational program, available in both English and Spanish, featured video vignettes of patients with ALL of various ages and racial/ethnic backgrounds and their parents, and addressed relevant health beliefs including perceived susceptibility to and severity of illness (i.e., leukemia), the purpose of 6MP, the perceived benefits and barriers to 6MP ingestion, and examples of how patients and parents had overcome such barriers (i.e., self-efficacy). The 20-minute interactive video was viewed in the clinic by parents and patients. Viewers selected vignettes featuring the patient/parent with whom they most closely identified in order to customize their learning experience. Animations and video clips featuring explanations from healthcare professionals were also integrated throughout the program. Technologic restrictions prevented viewers from skipping over required portions. The site research staff documented successfully viewing of the education program by patients/parents.

Web-based Text Message Reminders

A secure, HIPAA-compliant web-based application, *MEDACTIONPLAN/CT*, allowed healthcare providers to create customized 6MP schedules during routine clinic visits, specifying 6MP dose, time of administration, start date and duration. The healthcare provider used a streamlined interface within *MEDACTIONPLAN/CT* to activate automated SMS text messaging reminders to be delivered via cellular phone. Schedules were modified by healthcare providers as needed; reminders were updated concurrently. These customized reminders were delivered in the preferred language (English/Spanish) at the scheduled time of each 6MP dose to the 12-21-year-old patient <u>and</u> designated parent, and to the designated parent alone of the <12-year-old patient. The text messages included the drug name (6MP), prescribed dose (e.g., “take 2 tablets”), time due (e.g., “at 9:30 pm”), and name of healthcare provider (e.g., “Dr. Smith”). The entire process of creating the schedule and activating reminders was accomplished in the clinic. Parents/patients who did not have a cellular phone, or who had limited/no text-messaging capability on their personal plan, were issued cellular phones with unlimited text messaging capabilities for the duration of intervention.

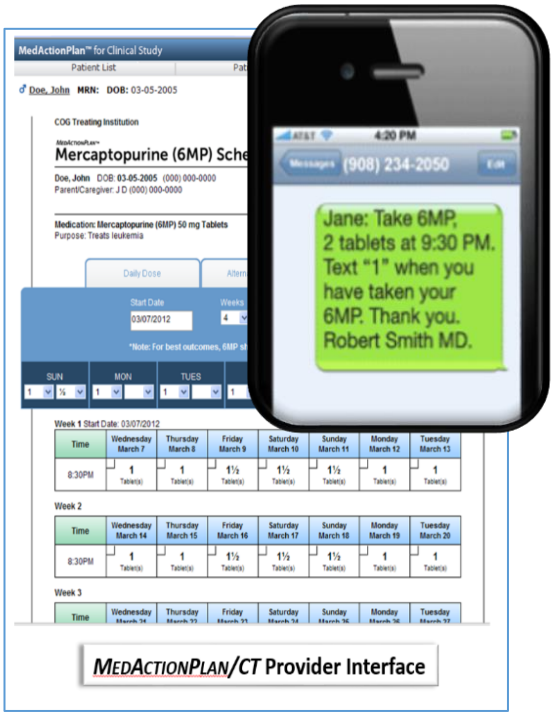

MEDACTIONPLAN/CT Provider Interface

©2020. Bhatia S et al. *JAMA Network Open*.

Directly supervised therapy (DST)

Once the patient had taken the 6MP, the ≥12-year-old patient and their parent, or the parent alone of the <12-year-old patient responded to the text message with a reply function on the cellular phone, providing central record of DST execution.

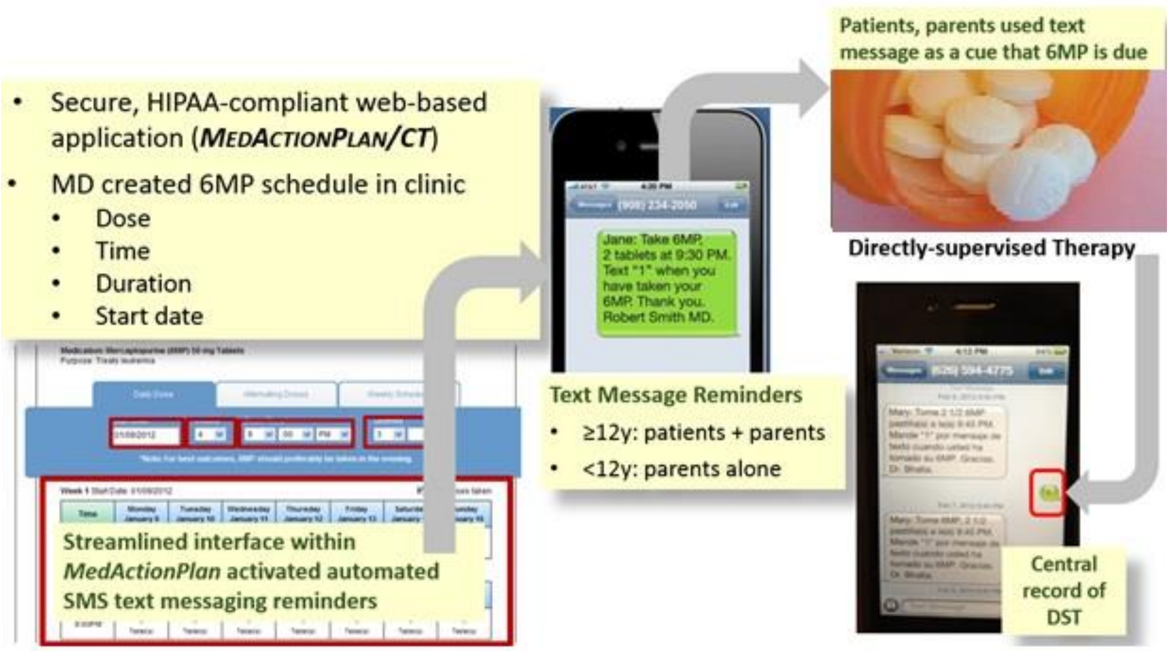

<u>**ADHERENCE MONITORING**</u>

MEMS® Adherence Monitoring Device

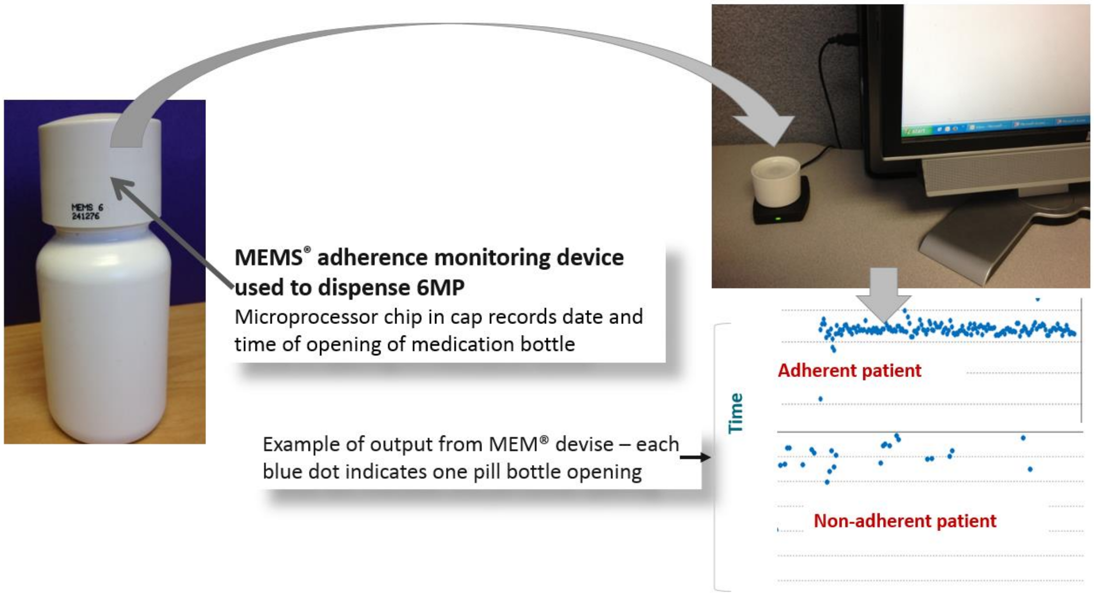

<u>**TRIAL PROCEDURES**</u>

Randomization occurred at the COG Statistics and Data Center on Day 1. For the first 28 days, all patients (IP, EDU) received 6MP from a MEMS® device without intervention; this period was used to calculate the baseline adherence

©2020. Bhatia S et al. *JAMA Network Open*.

rates. The intervention lasted for 16 weeks. Patients and designated parents on both arms viewed the educational video during their scheduled clinic visit on Day 29. For patients on the IP arm, the patient's healthcare provider activated automated text message reminders on study Days 29, 57, 85, 113 (days when patients returned to clinic for vincristine/steroid pulses), using *MedActionPlan/CT*. Participating sites submitted monthly reports (Maintenance Worksheets) for each patient, detailing prescribed 6MP dose for each day of the preceding month and dates when the prescriber held the 6MP dose for toxicity or illness. The study schema is outlined here.

Study Schema

Study Procedures	Timeline					
	Day 1	Day 29	Day 57	Day 85	Day 113	Day 141
Demographic questionnaire (IP, EDU)	X					
Maintenance worksheets (IP, EDU)		X	X	X	X	X
MIPE education (IP, EDU)		X				
Daily personalized text message reminders (IP)		→	→	→	→	
Daily DST (IP)		→	→	→	→	
MEMS monitoring of 6MP (IP, EDU)	→	→	→	→	→	
Return MEMS Caps (IP, EDU)						**X**

IP=Intervention Package: Education and daily personalized text message reminders prompting directly supervised therapy; EDU=Education alone; MIPE=Multimedia interactive patient education; DST=Directly supervised therapy; MEMS=Medication Event Monitoring System; 6MP=6-mercaptopurine

©2020. Bhatia S et al. *JAMA Network Open*.

eTable 1. Median Pre- to Post-Intervention Adherence Rates by Study Arm

Age Group	EDUCATION				INTERVENTION				
	N	Median	Q1	Q3	N	Median	Q1	Q3	P-value*
All	207	-1.0%	-5.4%	0.0%	226	0.0%	-2.6%	2.6%	< .001
≥12	71	-1.8%	-7.1%	0.0%	82	0.0%	-1.9%	3.6%	.004
<12	136	-0.9%	-4.5%	0.0%	144	0.0%	-2.7%	2.5%	.06

*Wilcoxon

©2020. Bhatia S et al. *JAMA Network Open*.

eTable 2. Sensitivity Analysis

**Sensitivity analysis***	**Comparison**	**OR**	**95% CI**		**p-value**
All patients- time points with missing adherence were considered non-adherent (<95%)	IP vs. EDU	1.3	0.9	1.797	0.1
All patients- time points with missing adherence were considered adherent (>=95%)	IP vs. EDU	1.4	1.0	1.928	0.03
All patients with missing paternal/maternal education were considered low education	IP vs. EDU	1.3	0.97	1.841	0.07
All patients with missing paternal/maternal education were considered higher education	IP vs. EDU	1.3	0.97	1.833	0.08

*The sensitivity analyses made the following assumptions: i) all patients with missing MEMs data were non-adherent (adherence rate <95%); all patients with missing MEMs data were adherent (adherence rate ≥95%); iii) all patients with missing parental education came from low-education families; iv) all patients with missing parent education came from high-education families.

OR=Odds Ratio; CI=Confidence Interval; IP=Intervention Package: Education and daily personalized text message reminders prompting directly supervised therapy; EDU=Education alone; MEMS=Medication Event Monitoring System

©2020. Bhatia S et al. *JAMA Network Open*.
