# Supplementary material for: Effect of a Daily Text Messaging and Directly Supervised Therapy Intervention on Oral Mercaptopurine Adherence in Children With Acute Lymphoblastic Leukemia: A Randomized Clinical Trial
Source: JAMA Netw Open. 2020 Aug 27;3(8):e2014205. doi: 10.1001/jamanetworkopen.2020.14205 (PMC7453312; doi:10.1001/jamanetworkopen.2020.14205)
Supplement: Supplement 3. — Data Sharing Statement [file jamanetwopen-3-e2014205-s003.pdf]

Data Sharing Statement

Bhatia. Effect of a Daily Text Messaging and Directly Supervised Therapy Intervention on Oral Mercaptopurine Adherence in Children With Acute Lymphoblastic Leukemia. *JAMA Netw Open*. Published August 27, 2020. 10.1001/jamanetworkopen.2020.14205

Data
Data available: No
